# Improved synthesis and polymerase recognition of 7-deaza-7-modified α-l-threofuranosyl guanosine analogs†‡

**DOI:** 10.1039/d4ra03029j

**Published:** 2024-06-19

**Authors:** Bhawna Barpuzary, Sergey Negria, John C. Chaput

**Affiliations:** a Department of Pharmaceutical Sciences, University of California Irvine CA 92697-3958 USA jchaput@uci.edu; b Department of Chemistry, University of California Irvine CA 92697-3958 USA; c Department of Molecular Biology and Biochemistry, University of California CA 92697-3958 USA; d Department of Chemical and Biomolecular Engineering, University of California Irvine CA 92697-3958 USA

## Abstract

Threofuranosyl nucleic acid (TNA), an artificial genetic polymer known for its nuclease resistance and acid stability, has grown in popularity as a genetically-encoded material for applications in synthetic biology and biomedicine. TNA oligonucleotide synthesis requires enzymatic or solid phase synthesis pathways that rely on monomer building blocks that are not commercially available and can only be obtained by chemical synthesis. Here we present a synthetic route to 7-deaza-7-modified tGTP and phosphoramidite analogs that is operationally simpler than our previously described strategy. The new methodology offers an HPLC-free route to tGTP analogs that are recognized by engineered TNA polymerases and can be incorporated with continued TNA synthesis.

## Abstract 2

Threofuranosyl nucleic acid (TNA), an artificial genetic polymer known for its nuclease resistance and acid stability, has grown in popularity as a genetically-encoded material for applications in synthetic biology and biomedicine.

## Graphical abstract

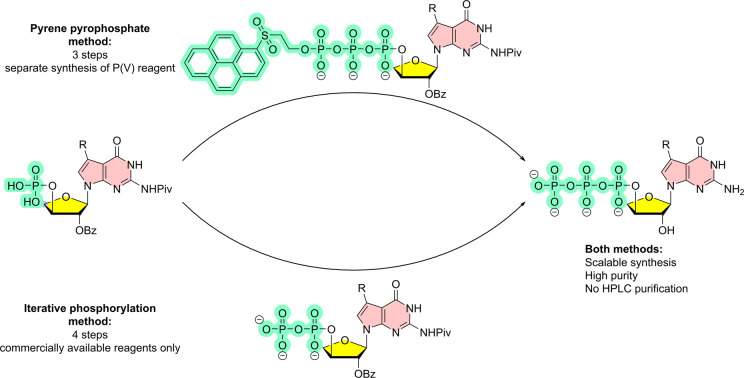

## Introduction

**Fig. 1:**
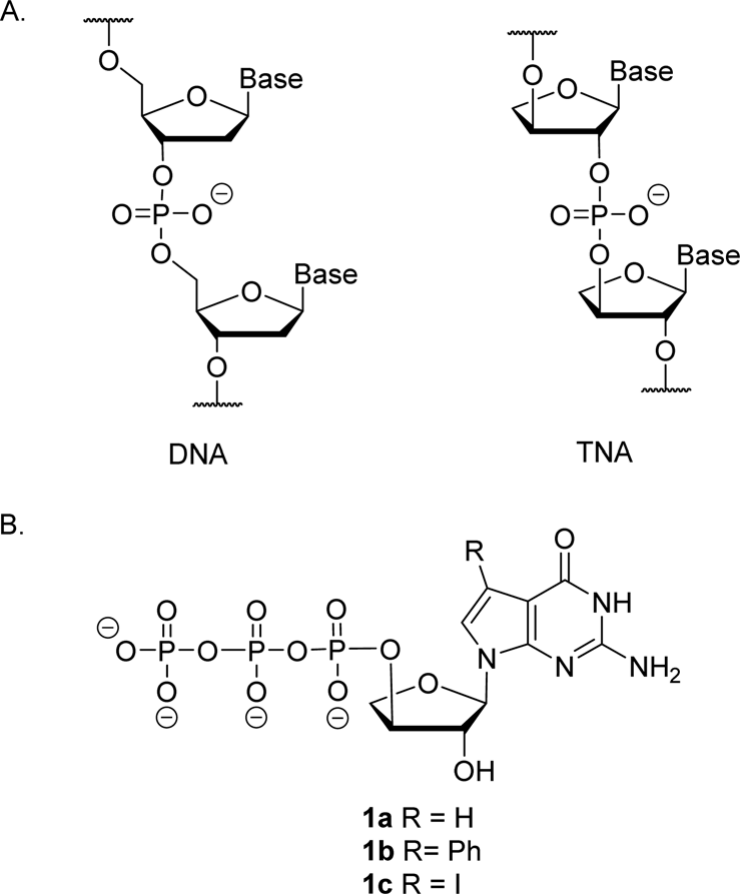
Molecular structures. (A) Constitutional structure for the linearized backbone of DNA (left) and (3′,2′)-α-l-threofuranosyl-nucleic acid, TNA (right). (B) Molecular structure of 7-deaza-7-modified guanosine TNA triphosphate.

Artificial genetic polymers (XNA), nucleic acids with distinct backbone structures, have gained significant popularity as viable materials for diagnostic and therapeutic applications.^1^ Ongoing efforts continue to focus on the development of XNA systems that can replicate by copying genetic information back and forth between DNA and XNA.^2–4^ α-l-Threofuranosyl nucleic acid (TNA)—a type of XNA that contains one less carbon atom in its backbone repeat unit than DNA and RNA—has received significant interest due to its ability to base pair with DNA and RNA (Fig. 1).^5–8^ TNA is evolvable,^9,10^ nuclease resistant,^11^ and acid stable.^12^ However, despite these interesting properties, TNA monomers are not commercially available, and require efficient routes for chemical synthesis.^13–16^

The synthesis of TNA phosphoramidite and triphosphate building blocks requires a multi-step synthetic strategy that is difficult and time consuming.^15^ In some cases, the synthetic strategy is substrate specific, warranting careful optimization. Other steps require expensive reagents, anhydrous conditions, and laborious purification techniques.^17^ Even the separation of the polar nucleoside triphosphates from polar impurities using high performance liquid chromatography (HPLC) is challenging, as it limits the scale of synthesis to tens of milligrams.^18^

**Fig. 2:**
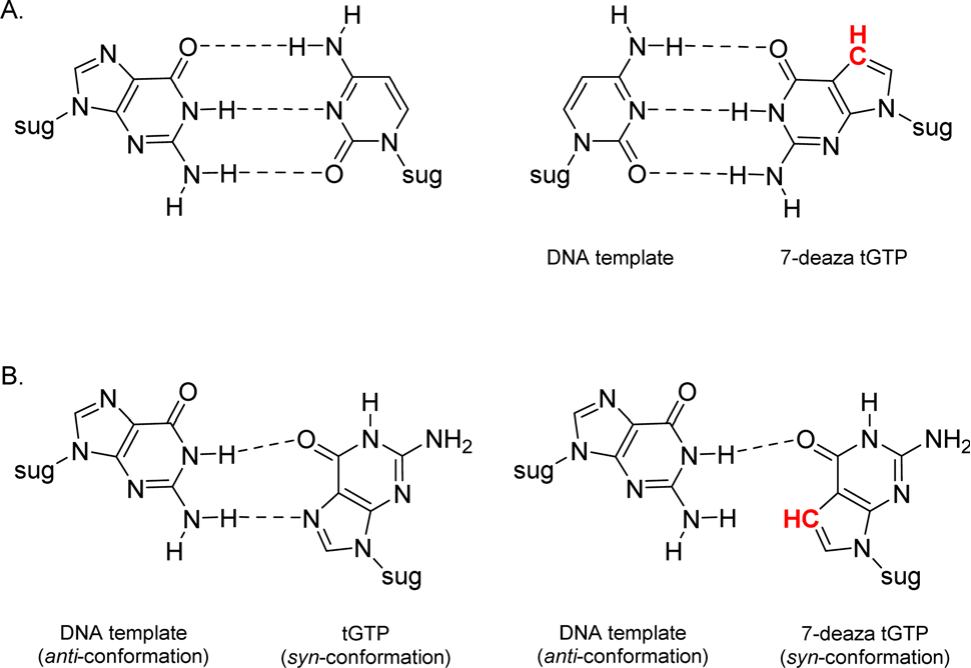
Molecular structures of (A) Watson–Crick base pairs and (B) G–G Hoogsteen base pairs with guanine and 7-deaza guanine.

Relative to other nucleobases, guanine nucleosides poses significant challenges due to their propensity for gel formation *via* G-quadruplex structures and low solubility due to the amphoteric nature of the N7 and N9 isomers that warrant suitable protection.^19^ The unnatural 7-deaza guanine (7dG), where the N-7 nitrogen atom of guanine is replaced by a carbon atom, forms Watson–Crick base pairs similar to a natural G:C base pair, but is incapable of Hoogsteen pairing in the major groove (Fig. 2). The substitution of tGTP with C^7^ tGTP improved the fidelity of older-generation TNA polymerases by avoiding the propensity for G–G mispairing during TNA synthesis.^20^ Recognizing the importance of 7-deaza-7-modified α-l-threofuranosyl guanosine 3′-triphosphates (Fig. 1) in enzymatic TNA synthesis, our lab reported the synthesis of these compounds from 6-chloro-7-iodo-pivaloyl-2-amino-7-deazaguanine nucleoside.^21^ This route utilizes typical inorganic tributylammonium pyrophosphate for the conversion of activated monophosphate to triphosphate and necessitates HPLC separation from highly polar side products, eventually reducing the amounts of desired triphosphates to milligrams. Moreover, the inorganic pyrophosphate is highly hygroscopic, making it difficult to use if it gets exposed to air, thus necessitating utilization of a fresh solution of reagent prior to each synthesis.

Here, we report an alternative approach for the synthesis of C^7^ tGTP 1a and C^7^-Ph tGTP 1b by investigating two distinct routes, one involving pyrene pyrophosphate and the other using an iterative phosphorylation method. Both routes circumvent the HPLC purification step typically performed to isolate polar triphosphates that are essential for engineered polymerase-mediated TNA synthesis. We utilized dibenzyl *N*,*N*-diisopropylphosphoramidite for the phosphorylation of 3′-hydroxyl group, ensuring preservation of the pivaloyl group on the exocyclic amine of monophosphate and 2′-hydroxyl of threose sugar. This benefits the iterative phosphorylation method by enabling the purification of phosphate intermediates using normal phase silica gel chromatography. This phosphorylating reagent also reduces the number of steps required for the synthesis of 7d7H tGTP when employing pyrene pyrophosphate strategy. Although significant amounts of 7d7H tGTP and 7d7Ph tGTP are produced in high purity by both routes, the pyrene pyrophosphate method is preferred owing to fewer number of steps. Additionally, we synthesized sufficient amounts of 7-deaza-7-modified α-l-threofuranosyl guanine 2′-phosphoramidites 14a and 14b for their potential utility in future investigations of solid-phase synthesis. The synthesis of the 7dG TNA nucleoside monomers on larger scale presents an opportunity to investigate their potential for enhancing the binding affinity of aptamers.

## Results and discussion

In prior works, we showed that replacing tGTP with 7-deaza-7-modified tGTPs significantly enhanced the fidelity of TNA replication up to 99% by reducing the number of G–G mispairings in older generation TNA polymerases.^20,21^ Noting the importance of 7-deaza-7-modified tGTPs, the synthesis was initiated with the conventional Vorbrüggen reaction between 6-chloro-7-iodo-2-pivaloylamino-7-deazaguanine and a protected threose sugar.^21^ Interestingly, the sugars containing 2′-hydroxy group in anti-configuration with respect to glycosidic bond proceed with high regio- and stereoselectivity.^22^ Subsequent deprotection of TBDPS group using TBAF produced 3′-hydroxyl precursor 2c, whereas for 7-phenyl derivative, additional Suzuki–Miyaura cross-coupling reaction was carried out before TBDPS deprotection to obtain 2b.^21^

**Scheme 1:**
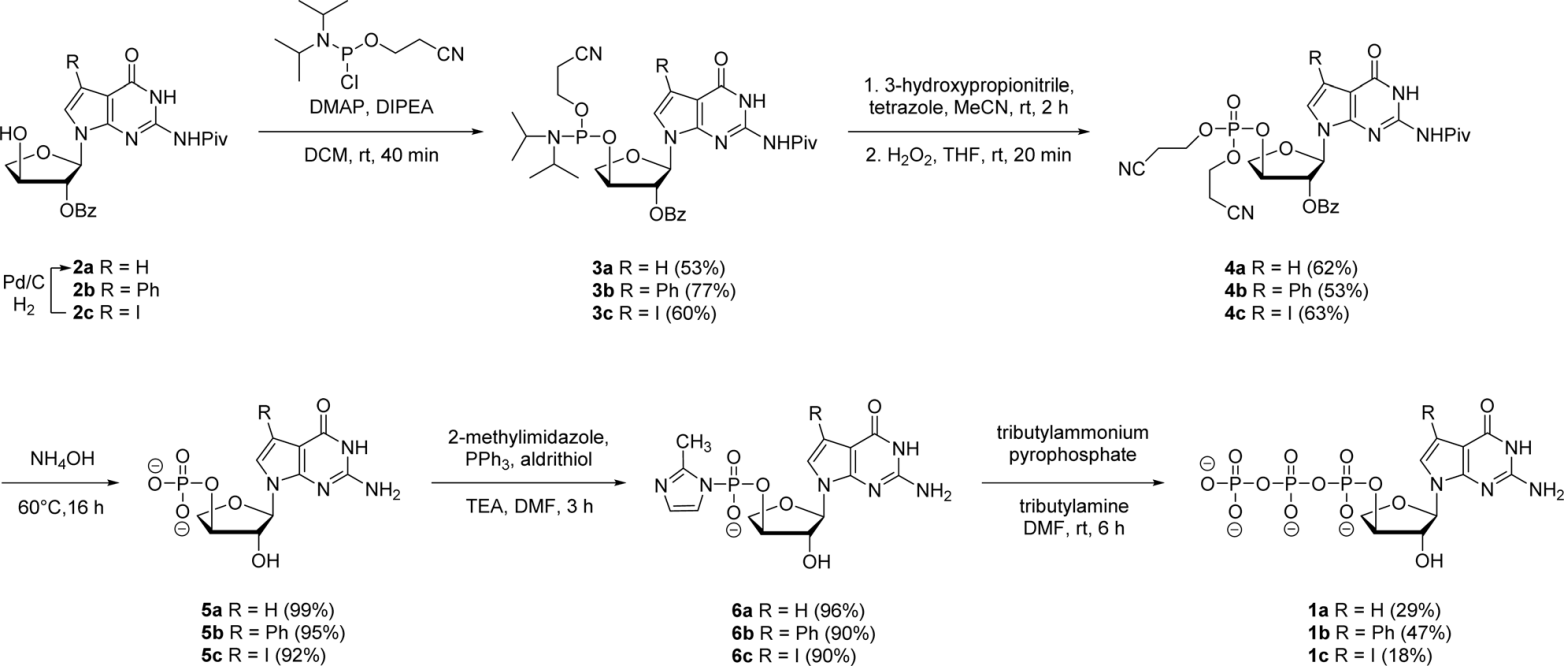
Reported synthesis of 7-deaza-7-modified guanosine TNA triphosphates.

**Scheme 2:**
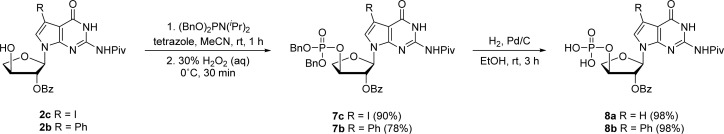
Synthesis of 7-deaza-7-modified guanosine TNA monophosphates.

Our synthetic study focused on improving the synthesis of 7d7Ph tGTP and 7d7H tGTP commenced with the 3′-hydroxyl nucleoside precursors 2b and 2c synthesized as per original protocol.^21^ The phosphorylation of 3′-hydroxyl in TNA is challenging due to its steric hindrance and reduced nucleophilicity compared to 5′-hydroxyl in DNA and RNA.^23^ Furthermore, phosphorylation using either Yoshikawa^24^ or Ludwig–Eckstein^25^ method results in a mixture of phosphorylated compounds where the desired compound is produced as a minor product. In our original protocol, 3′-hydroxyl compound 2 was first reacted with 2-cyanoethoxy-*N*,*N*-diisopropylchlorophosphoramidite to obtain the corresponding P(iii) phosphoramidite intermediate 3, which was converted to P(v) phosphotriester compound 4 using 30% H_2_O_2_ (Scheme 1).^21^ To obtain monophosphate 5, P(v) phosphotriester compound 4 was further treated with 30–33% aq NH_4_OH. However, this resulted in the removal of protecting groups on the exocyclic amine and 2′-hydroxyl of threose sugar thereby generating highly polar 7dG nucleoside monomers requiring tedious purification. Herein for the synthesis of P(v) phosphotriester, we utilized dibenzyl *N*,*N*-diisopropylphosphoramidite instead of 2-cyanoethoxy-*N*,*N*-diisopropylchlorophosphoramidite for phosphorylation because it allowed us to bypass the conversion of intermediates 3 to their bis-cyanoethyl phosphates 4. By eliminating the need for ammonium hydroxide deprotection of these cyanoethyl groups, subsequent purification of phosphate intermediates using normal phase silica gel chromatography was made more facile because of the preservation of protecting groups on the exocyclic amine and 2′-hydroxyl of sugar. The 3′-hydroxyl nucleosides 2b and 2c were treated with dibenzyl *N*,*N*-diisopropylphosphoramidite in the presence of tetrazole as an activator for 1 h at room temperature followed by the oxidation with 30% H_2_O_2_ for another 30 min to give corresponding P(v) phosphotriesters 7b and 7c with 99% and 78% yields, respectively (Scheme 2). Further hydrogenation with 10% Pd/C removed the benzyl groups and provided monophosphates 8a and 8b. Remarkably, this condition was also efficient for the conversion of 7-iodo guanosine to its 7-H derivative for 8a. The one-pot deprotection and hydrogenation of 7c resulted in the desired 7d7H guanosine monophosphate 8a, reducing the number of steps for 7d7H tGTP synthesis compared to the previously reported method where 2c was initially converted to 2a.

**Scheme 3:**
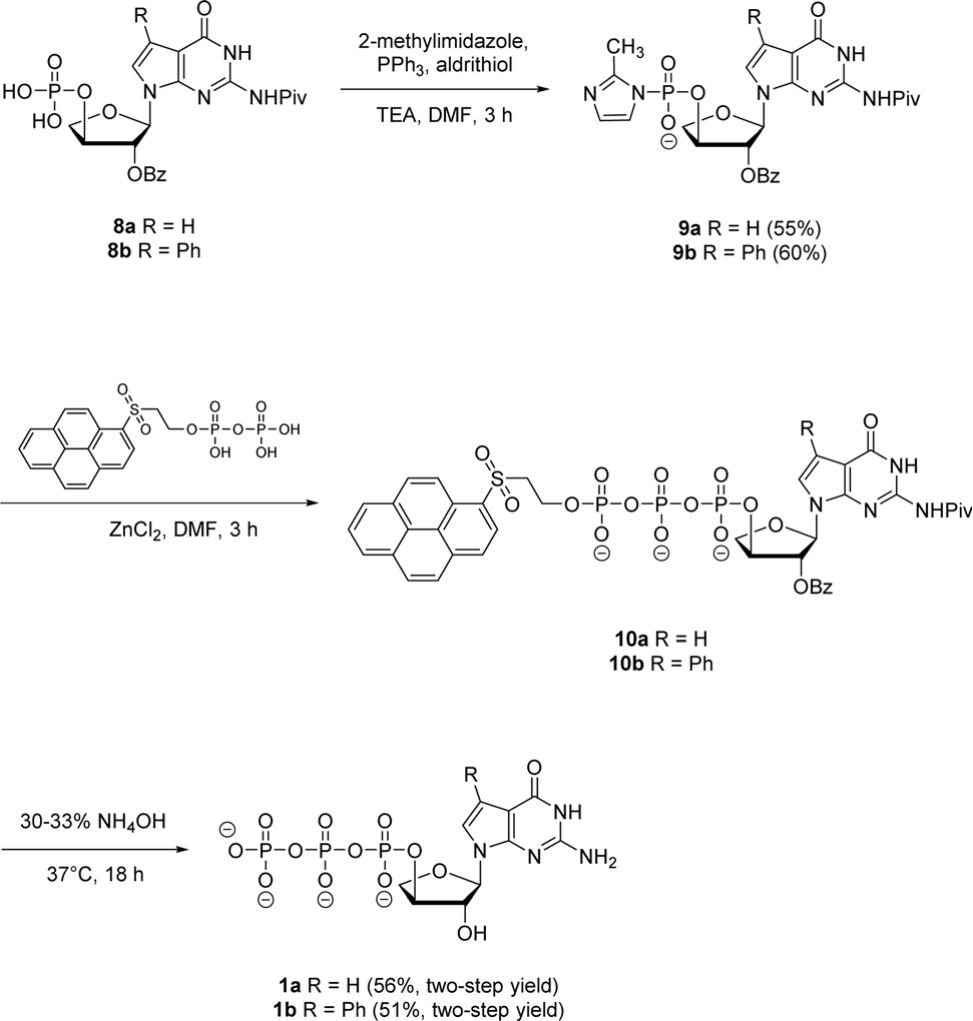
Synthesis of 7-deaza-7-modified guanosine TNA triphosphates by pyrene pyrophosphate method.

**Scheme 4:**
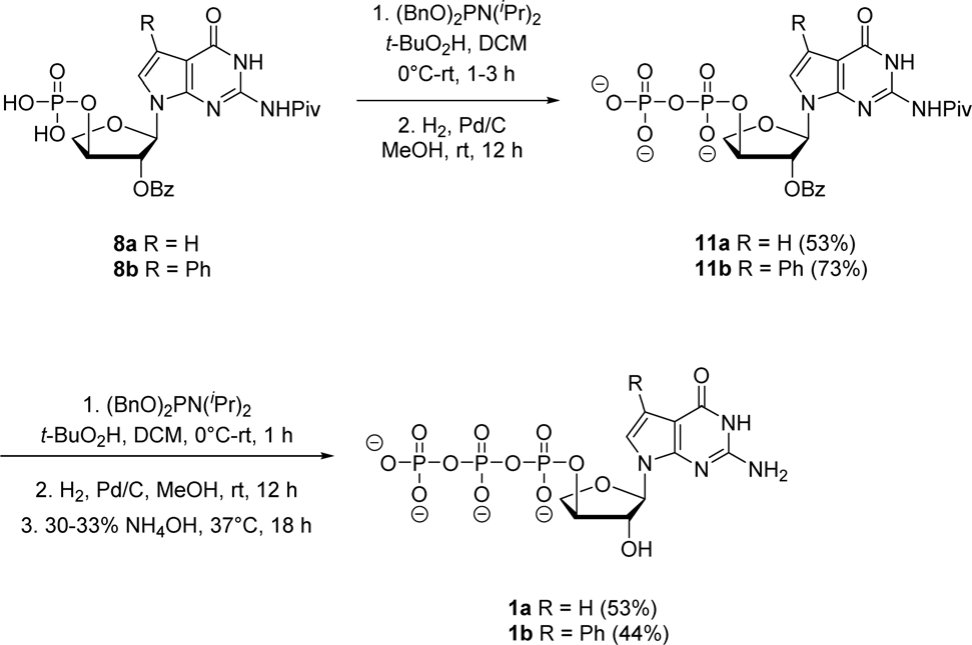
Synthesis of 7-deaza-7-modified guanosine TNA triphosphates by iterative phosphorylation method.

Next, both the pyrene pyrophosphate (Scheme 3) and iterative (Scheme 4) phosphorylation strategies were investigated for the synthesis of significant amounts of 7-deaza-7-modified tGTPs starting from monophosphates 2a and 2b. We evaluated an established approach for synthesizing nucleoside triphosphates that required P(v)-based organic pyrene pyrophosphate reagent to construct P–O bond formation between α- and β-phosphate positions.^26^ On coupling with imidazole-activated nucleoside monophosphate, this reagent produces protected nucleoside triphosphate that is sufficiently hydrophobic to be purified by normal phase silica gel chromatography. Following the similar protocol, nucleoside monophosphates 8a and 8b were activated with 2-methylimidazole in the presence of triphenylphosphine and aldrithiol. Then, the methylimidazole-activated monophosphates 9a and 9b were precipitated with sodium perchlorate and used in the coupling reaction with P(v) pyrene pyrophosphate without further purification to produce fully protected triphosphates 10a and 10b. The presence of protecting groups on the 2′-hydroxyl of sugar (2′-*O*-Bz) and the exocyclic amine of nucleobase also played a role in further decreasing the polarity of fully protected triphosphate, which facilitated column purification. Indeed, this strategy completely avoids the use of tedious and low-yielding HPLC separation of highly polar side products that is typically associated with the use of inorganic tributylammonium pyrophosphates for triphosphate synthesis. One-pot global deprotection of the protecting groups of 10a and 10b was achieved using 30–33% aq NH_4_OH to obtain large quantities (100–150 mg) of triphosphates 1a and 1b as sodium salts in high purity.

Jessen and co-workers developed an iterative strategy in which bisflurorenylmethyl-protected (bis-Fm) phosphoramidite is coupled to 5′-monophosphate to produce P(iii)–P(v) anhydride that is subsequently oxidized to a P(v)–P(v) anhydride to generate 5′-diphosphate.^27^ Repeating the same process produces 5′-triphosphates in large quantities that do not require HPLC purification. However, the bis-Fm phosphoramidite reagent is not readily available and to avoid HPLC the reaction should undergo complete conversion. In contrast to bis-Fm phosphoramidite reagent, we utilized the commercially available dibenzyl *N*,*N*-diisopropylphosphoramidite reagent to investigate the iterative phosphorylation strategy. We assumed that benzyl protection would make the phosphate intermediate sufficiently non-polar, facilitating its purification by normal phase silica gel chromatography. Therefore, for the second phosphorylation, the monophosphates 8a and 8b were reacted with dibenzyl *N*,*N*-diisopropylphosphoramidite in dry CH_2_Cl_2_ without an activator to generate P(iii)–P(v) anhydride adducts. *In situ* oxidation of the adducts with 5.5 M solution of *tert*-butyl hydroperoxide in decane (TBHP) for 30 min to 1 h produced the protected P(v)–P(v) diphosphate anhydride. After purification using normal phase silica gel chromatography, benzyl deprotection with H_2_ gas and 10% Pd/C catalyst generated free diphosphates 11a and 11b in 53% and 73% yields, respectively. The second iteration for introducing third phosphate group was accomplished with the same series of steps of phosphorylation, oxidation and deprotection with final 2′-*O* benzoyl deprotection in 30–33% aq NH_4_OH to give the corresponding triphosphates 1a and 1b. The triphosphates were isolated as sodium salts by precipitating with sodium perchlorate in acetone.

Both the pyrene pyrophosphate and iterative phosphorylation strategies could generate 7-deaza-7-modified tGTPs in significant amounts, high purity, and with operational ease. In case of pyrene phosphate strategy, the chemical synthesis of P(v) pyrene pyrophosphate is simple, inexpensive and scalable up to multigram (>100 mg), and the reagent can be stored for long time (∼up to six months to a year) at −20 °C for future utilization. The critical thing is the development of triphosphate substrate specific purification techniques including intricate optimization of gradients, thin-layer chromatography, careful loading on the column due to insoluble side products (*e.g.*, zinc salts), *etc.* However, once optimized, this pyrene pyrophosphate method can be utilized to synthesize nucleoside triphosphates on a gram scale without the need of additional HPLC. On the other hand, the iterative phosphorylation strategy, involving an additional step and purification, utilizes commercially available dibenzyl *N*,*N*-diisopropyl phosphoramidite as the only source of phosphorylating agent. Both strategies were able to produce triphosphates in similar overall yields but pyrene pyrophosphate is preferred due to its fewer number of steps and reduced number of purifications. Most important is that both strategies avoid HPLC purification and can provide sufficient amounts of triphosphates for *in vitro* studies (see ESI Table 1‡ for the benefits and drawbacks of previously reported tNTP synthesis methods).^10,21^

**Fig. 3:**
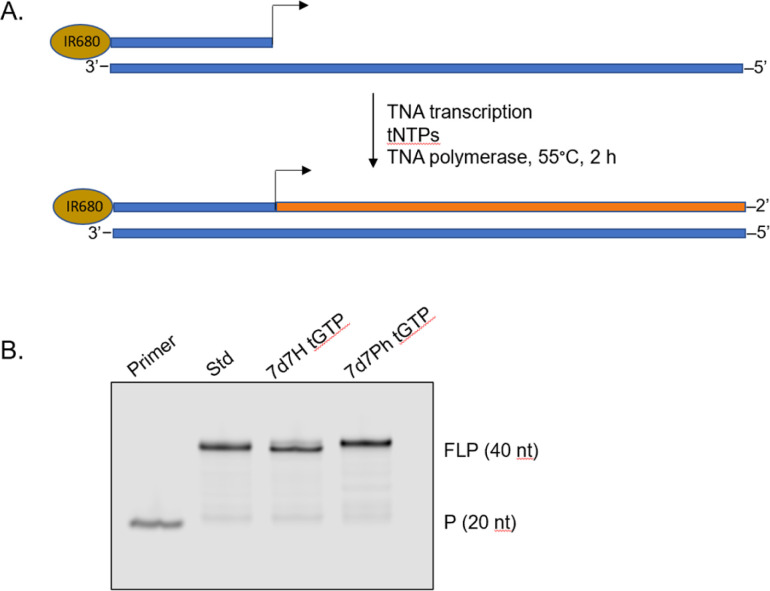
Polymerase-mediated synthesis of TNA with 7-deaza-7-modified guanosine TNA triphosphates. (A) Schematic representation of primer extension assay (primer (P) and DNA template in blue and full-length product (FLP) in orange). (B) Denaturing PAGE analysis.

To demonstrate that the 7-deaza-7-modified tGTP molecules were viable substrates for enzymatic TNA synthesis, a primer extension assay was performed using a laboratory-engineered TNA polymerase (Fig. 3).^28,29^ Accordingly, a 5′-IR680-labeled DNA primer annealed to DNA template containing 20 nucleotides (nt) unpaired region was incubated with tNTPs and the laboratory-engineered TNA polymerase for 2 h at 55 °C. In these reactions, tNTPs were either standard bases only or a mixture of tNTPs in which tGTP substrate was replaced with either 7d7H tGTP or 7d7Ph tGTP. The primer extension reactions were analysed by 12% denaturing polyacrylamide gel electrophoresis (PAGE) and showed full-length product in each case, which indicated that 7-deaza-7-modified tGTPs were efficiently recognized by engineered TNA polymerase.

**Scheme 5:**
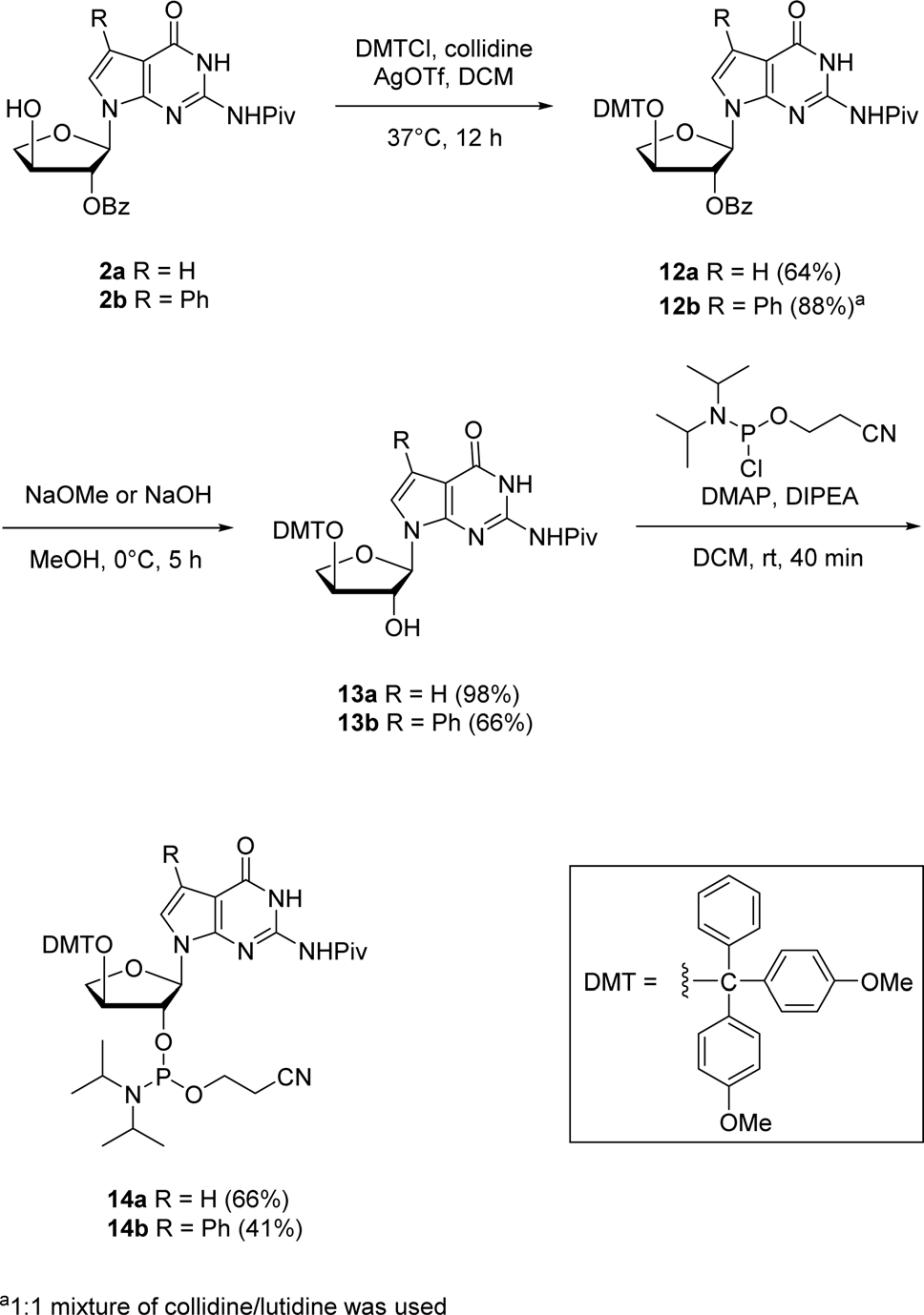
Synthesis of 7-deaza-7-modified guanosine TNA phosphoramidites.

The corresponding 7-deaza-7-modified TNA guanosine phosphoramidites were also synthesized for their possible incorporation into oligonucleotides *via* solid-phase synthesis in future investigations (Scheme 5). The 2′-protected nucleosides 2a and 2b were used as the starting materials for synthesizing the phosphoramidites 14a and 14b. The initial step involved the protection of 3′-hydroxyl position with 4,4-dimethoxytrityl (DMT) group. Since refluxing with DMT chloride in pyridine (p*K*_a_ ∼ 5.25) did not give any conversion of the starting material, we thought of utilizing a stronger bases like collidine and lutidine having p*K*_a_s of ∼7.48 and ∼6.75, respectively. Therefore, an alternative method which involved silver nitrate and collidine as a stronger base was employed. For 7-phenyl guanosine 2b, a 1 : 1 mixture of collidine/lutidine gave the DMT-protected product with moderate yield. The selective benzoyl deprotection of DMT-protected guanosine using methanol solutions of sodium methoxide for 12a and sodium hydroxide for 12b produced 2′-hydroxyl derivatives 13a and 13b while preserving the pivaloyl group on the nucleobase amine. The phosphorylation of 2′-hydroxy group using 2-cyanoethoxy-*N*,*N*-diisopropylchlorophosphoramidite in the presence of 4-dimethylaminopyridine (DMAP) and *N*,*N*-diisopropylethylamine (DIPEA) produced the corresponding 2′-phosphoramidites 14a and 14b in good yields.

## Conclusions

In summary, we describe an improved route for synthesizing 7-deaza-7-modified guanine nucleotide building blocks of TNA. The triphosphate monomers were prepared using a convergent and iterative phosphorylation strategy, both of which represent an improvement over our previous methodology and should be suitable for preparing hundreds of milligrams of substrate. We also demonstrated a more direct route to the phosphoramidite monomer for solid-phase synthesis.

## Author contributions

All authors conceived the project and designed the experiments. BB and SN performed the experiments. All authors analysed the data and contributed to manuscript preparation and revision.

## Conflicts of interest

There are no conflicts to declare.

## Supplementary Material

RA-014-D4RA03029J-s001
