# Supplementary material for: Improved synthesis and polymerase recognition of 7-deaza-7-modified α-l-threofuranosyl guanosine analogs
Source: RSC Adv. 2024 Jun 19;14(28):19701–6. doi: 10.1039/d4ra03029j (PMC11188673; doi:10.1039/d4ra03029j)
Supplement: RA-014-D4RA03029J-s001 [file RA-014-D4RA03029J-s001.pdf]

Electronic Supplementary Material (ESI) for RSC Advances.
This journal is © The Royal Society of Chemistry 2024

Supplementary Information

Improved Synthesis and Polymerase Recognition of 7-Deaza-7-Modified α-L-Threofuranosyl Guanosine Analogs

Bhawna Barpuzary,‡[a] Sergey Negria ‡[a] and John C. Chaput[a-d]*

[a]Department of Pharmaceutical Sciences, University of California, Irvine, CA 92697-3958 USA
[b]Department of Chemistry, University of California, Irvine, CA 92697-3958 USA
[c]Department of Molecular Biology and Biochemistry, University of California, CA 92697-3958 USA
[d]Department of Chemical and Biomolecular Engineering, University of California, Irvine, CA 92697-3958 USA

Contents

Table S1

Experimental Methods

Compound Characterization

References

Spectra Images

Methods	Advantages	Drawbacks
Extensions of classic Ludwig-Eckstein Method[1-4]	▪ Avoids $POCl_3$ (Yoshikawa method for 5′-nucleotides[5])	▪ Moisture-sensitive<br>▪ Poor regioselectivity of 2′- and 3′-DMT in some cases<br>▪ Require sensitive inorganic pyrophosphate<br>▪ Complex HPLC chromatograms
NCS Method[6]	▪ One-pot method<br>▪ Does not require strict anhydrous solvents<br>▪ Easier HPLC purification than previously reported methods	▪ Stoichiometry of water should be controlled<br>▪ Require sensitive inorganic pyrophosphate<br>▪ Major side products with low desired product<br>▪ Require HPLC purification<br>▪ Scalability issue
Extensions of Hoard and Ott Method[7-9]	▪ Higher yields of 3′-monophosphate intermediates	▪ Sensitive inorganic pyrophosphate<br>▪ Require HPLC purification<br>▪ Scalability issue
Pyrene Pyrophosphate Method[10]	▪ Avoids HPLC purification<br>▪ High yielding<br>▪ Scalable<br>▪ High Purity<br>▪ Reagent storage at -20°C	▪ Reagent synthesis<br>▪ Intricate optimization of purification gradient<br>▪ Insoluble Zn salts
Extension of Jessen Iterative Phosphorylation[11,12]	▪ Avoids HPLC<br>▪ Scalable<br>▪ High Purity<br>▪ Commercially available reagent	▪ More number of steps than pyrene pyrophosphate method<br>▪ Additional purifications required

Table S1 Summary of previous methods for α-L-threofuranosyl (TNA) triphosphate synthesis.

Experimental Methods

General Information

All moisture sensitive reactions were performed in anhydrous solvents under an argon or nitrogen atmosphere. All commercial reagents, solvents and anhydrous solvents were used without further purification. Reaction progresses were monitored by thin layer chromatography using glass-backed analytical Silica Plate with UV active F254 indicator. Flash column chromatography was performed with Silica Flash® P60 silica gel (40-63 µm particle size) for most of the crude reaction mixture. Yields are reported as isolated yields of pure compounds. ^{1}H, ^{13}C, and ^{31}P NMR spectra were analysed on 400 MHz NMR spectrometer (Bruker DRX) at the University of California, Irvine NMR Facility. ^{1}H values are reported in parts per million relative to Me_4Si or corresponding deuterium solvents used as an internal standard. ^{13}C values are reported in parts per million relative to corresponding deuterium solvents used as an internal standards. ^{31}P NMR values are reported in parts per million relative to an external standard of 85% H3PO4. Splitting patterns are designated as follows: s, singlet; d, doublet; dd, doublet of doublets; t, triplet; m, multiplet. High resolution mass spectrometry (HRMS) data were acquired using the electrospray ionization time-of-flight (ESI-TOF) method at the University of California, Irvine Mass Spectrometry Core Facility.

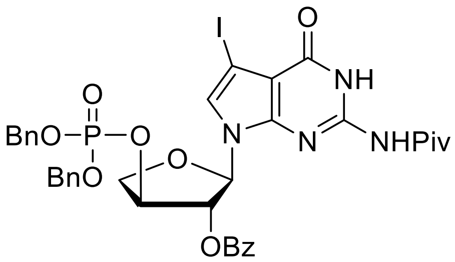

7-Deaza-7-iodo-N^2-pivaloyl-9-(2′-*O*-benzoyl-α-L-threofuranosyl) guanine 3′-dibenzylmonophosphate (7c): To a mixture containing **2c** (1.10 g, 1.9422 mmol, 1 equiv) and tetrazole (245 mg, 3.4956 mmol, 1.8 equiv) in an anhydrous solvent 1:1 MeCN/DCM (13 mL) was added 1.3 equiv of $(BnO)_2PN(^iPr)_2$ (850 µL, 2.5246 mmol) under a nitrogen atmosphere. The mixture was stirred at room temperature for 3 h (monitored by TLC). To the reaction mixture was added 30% aqueous H_2O_2 (0.45 mL, 3.953 mmol, 2.5 equiv) at 0°C, and the resulting mixture was stirred at room temperature for 20 min. At which the reaction was concentrated under reduced pressure, the crude was diluted with 15-20 times volume of DCM and the organic layer was sequentially washed with saturated $NaHCO_3$ (aq), brine, and water. The combined organic layer was dried with Na_2SO_4, and evaporated under diminished pressure to obtain crude. The crude residue was purified by liquid loading onto silica with eluents (0%-5% MeOH/DCM) to give pure product (1.45 g, 1.7542 mmol, 90%) as white solid.

R_f 0.7 (TLC MeOH/DCM 1:10); ^{1}H NMR (400 MHz, $CDCl_3$) δ 11.75 (s, 1H), 8.29 (s, 1H), 8.02 – 7.97 (m, 2H), 7.66 – 7.59 (m, 1H), 7.48 (t, J = 7.8 Hz, 2H), 7.37 – 7.27 (m, 10H), 7.05 (s, 1H), 6.03 (d, J = 2.1 Hz, 1H), 5.82 (dt, J = 2.2, 1.2 Hz, 1H), 5.12 – 5.05 (m, 4H), 5.05 – 4.98 (m, 1H), 4.32 (dt, J = 10.9, 1.5 Hz, 1H), 4.16 (ddd, J = 10.9, 4.3, 1.4 Hz, 1H), 1.27 (s, 9H); ^{13}C NMR (101 MHz, $CDCl_3$) δ 179.90, 164.70,

156.81, 147.29, 146.72, 135.27, 135.22, 135.21, 135.16, 134.01, 129.90, 128.91, 128.89, 128.76, 128.76, 128.72, 128.56, 128.20, 128.15, 125.26, 105.94, 88.89, 80.84, 80.77, 79.43, 79.38, 77.30, 72.89, 72.84, 70.15, 70.09, 55.90, 40.24, 26.97; ^{31}P NMR (162 MHz, $CDCl_3$) δ -2.55; HRMS (ESI-TOF) calcd. for $C_{36}H_{37}IN_4O_9P$ $[M+H]^+$ 827.1349, observed 827.1343.

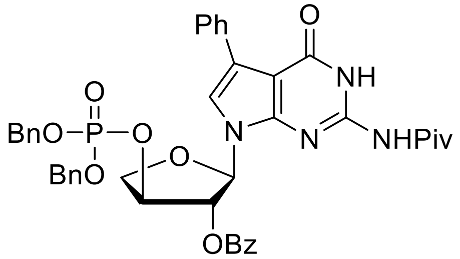

7-Deaza-7-Phenyl-N^2-pivaloyl-9-(2′-*O*-benzoyl-α-L-threofuranosyl) guanine 3′-dibenzylmonophosphate (7b): Prepared according to the procedure for **7c** with 1.00 g **2b** (1.94 mmol, 1 equiv), 267 mg tetrazole (2.88 mmol, 2 equiv), 0.91 mL $(BnO)_2PN(^iPr)_2$ (2.9 mmol, 1.5 equiv), 20 mL MeCN/DCM 1:1, 0.6 mL 30% H_2O_2 (3 equiv). Column chromatography (EtOAc/Hexane 1:2 to 1:1). Yield: 1.18 g (1.52 mmol, 78%) as a white solid.

R_f 0.5 (TLC MeOH/DCM 1:10); ^{1}H NMR (400 MHz, CD_3CN) δ 11.82 (s, 1H), 9.16 (s, 1H), 8.05 – 7.98 (m, 2H), 7.80 (dd, *J* = 7.4, 1.8 Hz, 2H), 7.64 (ddt, *J* = 8.6, 7.6, 1.5 Hz, 1H), 7.49 (t, *J* = 7.8 Hz, 2H), 7.33 – 7.19 (m, 14H), 6.16 (d, *J* = 2.5 Hz, 1H), 6.02 – 5.99 (m, 1H), 5.14 (ddt, *J* = 6.4, 4.0, 1.9 Hz, 1H), 5.05 – 4.99 (m, 4H), 4.38 (dt, *J* = 11.0, 1.6 Hz, 1H), 4.23 (ddd, *J* = 10.9, 4.3, 2.0 Hz, 1H), 1.22 (s, 9H); ^{13}C NMR (101 MHz, CD_3CN) δ 181.14, 165.00, 157.29, 148.94, 147.42, 135.91, 135.83, 135.76, 133.92, 133.57, 129.78, 128.99, 128.78, 128.60, 128.22, 128.13, 128.01, 127.97, 126.56, 121.61, 118.33, 102.20, 88.81, 80.64, 80.58, 79.50, 79.45, 72.51, 72.45, 69.67, 69.61, 40.10, 25.99; ^{31}P NMR (162 MHz, CD_3CN) δ -3.02. HRMS (ESI-TOF) calcd. for $C_{42}H_{41}IN_4O_9PNa$ $[M+Na]^+$ 799.2509, observed 799.2535.

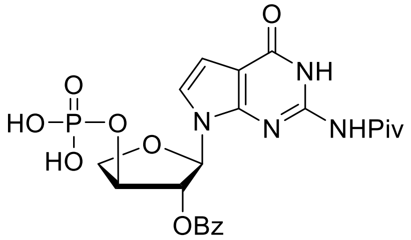

7-Deaza-N^2-pivaloyl-9-(2′-*O*-benzoyl-α-L-threofuranosyl) guanine 3′-monophosphate (8a). The mixture of **7c** (700 mg, 0.8468 mmol, 1 equiv) and 10% Pd/C (210 mg) was dried under vacuum for 30 min, and to it anhydrous MeOH (70 mL) and TEA (175 μL) were added followed by adding a H_2 gas balloon. The mixture was stirred at room temperature for 3 h with monitoring by TLC. Then the solution was purged with nitrogen, filtered over a pad of celite, and washed with 100 mL MeOH two to three times. The filtrate was collected, evaporated under reduced pressure to afford a white TEA salt of product (600 mg, 0.8301 mmol, 98%).

R_f 0.2 (TLC MeOH/DCM 1:10 (1% TEA)); ^{1}H NMR (400 MHz, CD_3OD) δ 8.05 – 7.99 (m, 2H), 7.64 – 7.58 (m, 1H), 7.51 – 7.44 (m, 3H), 6.61 (d, *J* = 3.7 Hz, 1H), 6.33 (d, *J* = 1.9 Hz, 1H), 5.70 (q, *J* = 1.1 Hz, 1H),

5.01 (ddt, J = 7.8, 3.4, 1.7 Hz, 1H), 4.46 – 4.40 (m, 1H), 4.27 (dd, J = 10.3, 4.2 Hz, 1H), 3.14 (q, J = 7.3 Hz, 9H, TEA), 1.26 (t, J = 7.3 Hz, 14H, TEA), 1.23 (s, 9H); ^{13}C NMR (101 MHz, CD_3OD) δ 181.55, 165.29, 158.84, 148.80, 146.92, 133.55, 129.45, 128.96, 128.40, 121.90, 103.96, 102.96, 88.09, 82.78, 82.72, 77.19, 77.14, 73.58, 73.54, 46.33 (TEA), 39.97, 25.64, 7.88 (TEA); ^{31}P NMR (162 MHz, CD_3OD) δ 0.03; HRMS (ESI-TOF) calcd. for $C_{28}H_{41}N_5O_9P$ $[M+H+N(CH_2CH_3)_3]^+$ 622.2642, observed 622.2658.

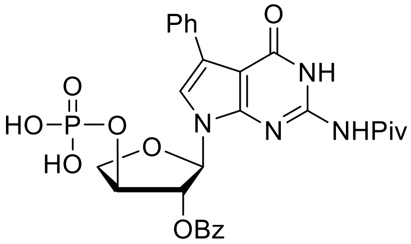

7-Deaza-7-Phenyl-N^2-pivaloyl-9-(2′-*O*-benzoyl-α-L-threofuranosyl) guanine 3′-monophosphate (8b): Prepared according to the procedure for **8a** with 700 mg **7b** (0.9 mmol, 1 equiv), 140 mg 10% Pd/C (0.2 mass equiv), 50 mL EtOH. Yield: 525 mg (0.88 mmol, 98% as free acid) as a white solid.
R_f 0.3 (TLC 1:4 MeOH/DCM (1% TEA)). ^{1}H NMR (400 MHz, CD_3OD) δ 8.01 (d, J = 7.7 Hz, 2H), 7.84 (d, J = 7.6 Hz, 2H), 7.60 (t, J = 7.4 Hz, 1H), 7.51 – 7.40 (m, 3H), 7.31 (t, J = 7.5 Hz, 2H), 7.20 (t, J = 7.3 Hz, 1H), 6.42 (s, 1H), 5.77 (s, 1H), 5.08 (s, 1H), 4.48 (d, J = 10.6 Hz, 1H), 4.29 (d, J = 9.2 Hz, 1H), 1.21 (s, 9H); ^{13}C NMR (101 MHz, CD_3OD) δ 181.45, 165.10, 158.51, 149.74, 147.18, 133.61, 133.49, 129.51, 128.76, 128.40, 128.13, 127.73, 126.22, 122.31, 118.29, 101.47, 87.95, 81.94, 78.29, 72.87, 46.99, 39.97, 25.62; ^{31}P NMR (162 MHz, CD_3OD) δ -1.14; HRMS (ESI-TOF) calcd. for $C_{28}H_{29}N_4O_9PNa$ $[M+Na]^+$ 619.1570, observed 619.1586.

Pyrene Pyrophosphate Method

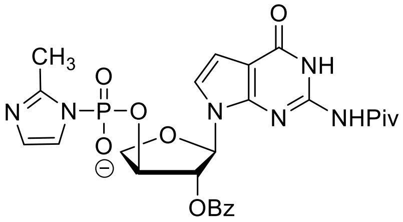

7-Deaza-N^2-pivaloyl-9-(2′-*O*-benzoyl-α-L-threofuranosyl) guanine 3′-*O*-monophosphor-2-methylimidazolide (9a): To a solution containing the **8a** (600 mg, 0.8301 mmol, 1 equiv) in anhydrous DMF (10 mL) under a nitrogen atmosphere was slowly added anhydrous TEA (0.99 mL, 7.102 mmol, 8.5 equiv) at 0 °C. After 5 min of stirring, 2-methylimidazole (473 mg, 5.761 mmol, 6.9 equiv) was added followed by triphenylphosphine (756 mg, 2.882 mmol, 3.5 equiv). After 10 min of stirring at room temperature, 2,2′-dipyridyl disulfide (635 mg, 2.882 mmol, 3.5 equiv) was added and stirring was continued for an additional 3 h at room temperature with monitoring by analytical HPLC (mobile phase: MeCN/0.05 M TEAA buffer). After consumption of the starting material, the product was precipitated by adding the reaction mixture dropwise with stirring to 300 mL of diethyl ether. The precipitate was collected by centrifuging at 4400 rpm for 5 min at room temperature. The supernatant was discarded, and the pellet

was resuspended with minimal amount of DCM. The solution was added dropwise to a premade solution of diethyl ether (300 mL)/TEA (15 mL) containing sodium perchlorate (1.17 g, 9.555 mmol, 11.5 equiv) for a second precipitation. The suspended solid was centrifuged at 4400 rpm for 5 min at room temperature, the supernatant was discarded, and the pellet was washed twice with mixed solvent (20 mL ether) and dried under high vacuum to afford the product **9a** (280 mg, 0.4616 mmol, 55%).

^{31}P NMR (162 MHz, CD_3OD) δ -10.21; HRMS (ESI-TOF) calcd. for $C_{26}H_{28}N_6O_8PNa_2$ [M-H+2Na]$^+$ 629.1501, observed 629.1500.

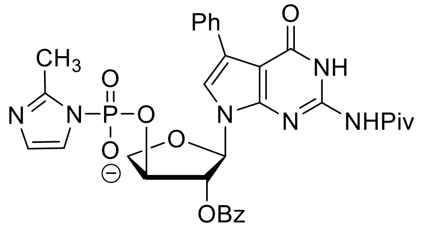

7-Deaza-7-Phenyl-N^2-pivaloyl-9-(2′-*O*-benzoyl-α-L-threofuranosyl) guanine 3′-*O*-monophosphor-2-methylimidazolide (9b): Prepared according to the procedure for **9a** with 250 mg **8b** (0.42 mmol, 1 equiv), 5 mL DMF, 0.35 mL TEA (2.52 mmol, 6 equiv), 172 mg 2-methylimidazole (2.1 mmol, 5 equiv), 275 mg PPh_3 (1.05 mmol, 2.5 equiv), 231 mg 2,2′-dipyridyl disulfide (1.05 mmol, 2.5 equiv); 200 mL diethyl ether for first precipitation, 200 mL of diethyl ether/EtOAc/TEA (5:10:1) containing 8 equivalents of sodium perchlorate for second precipitation. Yield: 166 mg (60%) as a white solid.

^{31}P NMR (162 MHz, $CDCl_3$) δ -7.81; HRMS (ESI-TOF) calcd. for $C_{32}H_{33}N_6O_8PNa$ [M+Na]$^+$ 683.1995, observed 683.1991.

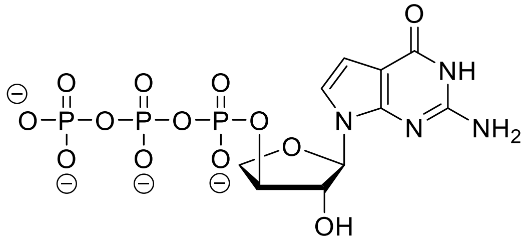

7-Deaza-(α-L-threofuranosyl) guanine 3′-triphosphate (1a):

Step 1: To a mixture containing the activated nucleoside monophosphate **9a** (280 mg, 0.4616 mmol, 1 equiv) and 1.2 equiv of 1-(2-(pyrenesulfonyl)ethyl)pyrophosphate (260 mg, 0.5539 mmol) was added along with 8 equiv of a premade solution of $ZnCl_2$ (503 mg, 3.693 mmol) in 2.4 mL anhydrous DMF under a nitrogen atmosphere. The mixture was stirred at room temperature for 5 h and the reaction progress was monitored by HPLC (MeCN/0.05 M TEAB buffer, from 0% to 70% over 42 min). After consumption of the starting material, the product was precipitated by adding the reaction mixture dropwise to stirred diethyl ether (170 mL). The precipitate was collected by centrifuging at 4400 rpm for 5 min at room temperature. The pellet was resuspended in 30 mL 20% H_2O in MeCN containing 2% *N*,*N*-diisopropylethylamine (DIPEA) and centrifuged at 4400 rpm for 5 min and the supernatant was collected. The above process was repeated two times and the combined supernatants were evaporated under

diminished pressure. The crude was loaded on a silica gel column (packed with 2% H_2O/isopropanol containing 1% DIPEA) by liquid loading (2 mL of 2% H_2O/isopropanol containing 1% DIPEA) with eluents 5% H_2O/isopropanol containing 1% DIPEA and then 2% to 7% H_2O in (isopropanol/MeCN 1:1) containing 1% DIPEA. The fractions containing the product were collected and evaporated under diminished pressure at 30-40 °C to afford pure 450 mg fully protected nucleoside triphosphate. The purity was confirmed by its ^{31}P NMR (see the attached NMR below).

Step 2: A solution of fully protected nucleoside triphosphate 450 mg in 30-33% aqueous NH_4OH was stirred for 18 h at 37°C in a sealed tube. After the reaction, the solvent was evaporated under diminished pressure. The solid was resuspended with MilliQ water (5 mL) and the aqueous solution was washed with DCM (10 mL) and EtOAc (10 mL). The organic portion was discarded and the aqueous extract was collected, and evaporated under diminished pressure. The crude solid was resuspended with 2 mL of RNAse free water, filtrated by 0.22 μm syringe filter and dropwise added to the forty times volume of acetone (80 mL) at room temperature containing $NaClO_4$ (850 mg, 6.924 mmol, 15 equiv). The resulting suspension was centrifuged at 4400 rpm for 5 min at room temperature. The supernatant was discarded and the pellet was washed with organic solution (acetone/DCM, 10:1, 2 x 30 mL) to afford the **1a** (150 mg, 0.2585 mmol, 56% two-step yield).

^{1}H NMR (400 MHz, D_2O) δ 7.06 (d, *J* = 3.8 Hz, 1H), 6.48 (t, *J* = 3.9 Hz, 1H), 5.95 (d, *J* = 2.7 Hz, 1H), 4.92 (d, *J* = 8.7 Hz, 1H), 4.26 (d, *J* = 2.8 Hz, 2H); ^{31}P NMR (162 MHz, D_2O) δ -5.71, -11.56 (d, *J* = 17.7 Hz), -20.31; HRMS (ESI-TOF) calcd. for $C_{10}H_{11}N_4O_{13}P_3Na_4H$ $[M-3H+4Na]^+$ 580.9205, observed 580.9201.

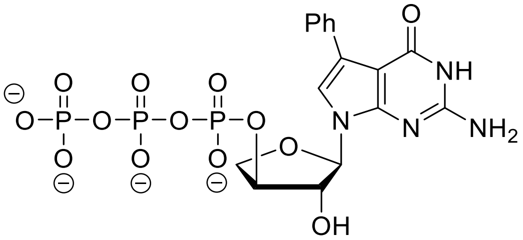

7-Deaza-7-Phenyl-(α-L-threofuranosyl) guanine 3′-triphosphate (1b):

Step 1: Prepared according to the procedure for **1a** with 204 mg **9b** (0.31 mmol, 1 equiv), 173 mg 1-(2-(pyrenesulfonyl)ethyl)pyrophosphate (0.37 mmol, 1.2 equiv), 506 mg $ZnCl_2$ (3.7 mmol, 12 equiv) in 2.5 mL anhydrous DMF; 200 mL diethyl ether for first precipitation, 50 mL 20% H_2O in MeCN containing 2% DIPEA for resuspension. Column chromatography (dry-packing loading, Acetone/MeCN/H_2O 5:4.5:0.5, 2% DIPEA added).

Step 2: Prepared according to the procedure for **1a** with 253 mg **10b** (0.176 mmol, 1 equiv), 5 mL 30-33% aqeous NH_4OH; 5 mL MilliQ water, 10 mL DCM, 10 mL EtOAc for first washing; 2 mL RNAse free water, 80 mL acetone containing 323 mg $NaClO_4$ (2.64 mmol, 15 equiv) for precipitation; 2x30 mL Acetone/DCM 10:1 for second washing. Yield 104 mg (0.158 mmol, 51%, two-step yield).

^{1}H NMR (400 MHz, D_2O) δ 7.69 – 7.65 (m, 2H), 7.43 – 7.36 (m, 2H), 7.33 – 7.28 (m, 1H), 7.19 (s, 1H), 6.01 (d, J = 2.8 Hz, 1H), 4.98 (d, J = 8.8 Hz, 1H), 4.81 (s, 1H), 4.30 (d, J = 3.0 Hz, 2H); ^{31}P NMR (162

MHz, D_2O) δ -5.66, -11.45 (d, J = 15.5 Hz), -18.98; HRMS (ESI-TOF) calcd for $C_{16}H_{16}N_4O_{13}P_3Na_4$ [M-3H+4Na]$^+$ 656.9518; observed 656.9501.

Iterative Phosphorylation Method

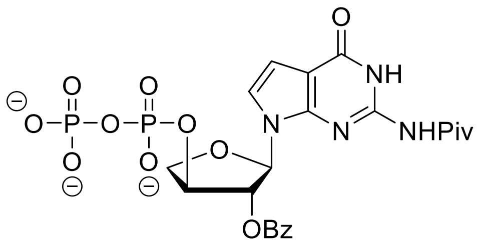

7-Deaza-(α-L-threofuranosyl) guanine 3′-diphosphate (11a):

Step 1: To a solution of **8a** (200 mg, 0.2767 mmol, 1 equiv) in 0.8 mL of anhydrous DCM was added $(BnO)_2PN(^iPr)_2$ (106 µL, 0.3320 mmol, 1.2 equiv) and stirred the reaction for 1-3 h at room temperature under nitrogen atmosphere. After the starting material was consumed, t-BuO_2H (5.5 M in $C_{10}H_{22}$) (125 µL, 0.6917 mmol, 2.5 equiv) was added at 0°C and continued the reaction for another 30 min. The solution was then diluted with 10-20 times volume of DCM and the organic layer was sequentially washed with brine, and water (2x50 mL). The combined organic extracts were dried with Na_2SO_4, and evaporated under diminished pressure. The crude residue was purified by wet loading over silica gel with eluents MeOH/DCM (1% to 5% containing 1% DIPEA) and the fractions containing the product were collected and evaporated to afford as white solid as DIPEA salt of product (137 mg, 0.1505 mmol, 54%).

Step 2: To the above obtained solid in a round bottom flask was added 50 mg of 10% Pd/C which was evacuated for 20 min and then added 2 mL of anhydrous MeOH. The reaction mixture was stirred in presence of H_2 balloon for 12 h at room temperature. The suspension was filtered through celite, washed with MeOH (100 mL) and concentrated to afford the product **11a** as white DIPEA salt of diphosphate (110 mg, 0.1505 mmol, 53% two-step yield).

^{1}H NMR (400 MHz, CD_3OD) δ 7.99 (d, J = 7.2 Hz, 2H), 7.59 (t, J = 6.8 Hz, 1H), 7.51 – 7.39 (m, 3H), 6.64 – 6.55 (m, 1H), 6.31 (s, 1H), 5.71 (s, 1H), 5.19 (s, 1H), 4.50 (s, 1H), 4.28 (s, 1H), 1.21 (s, 9H); ^{13}C NMR (101 MHz, CD_3OD) δ 181.52, 165.27, 158.79, 148.77, 146.86, 133.50, 129.50, 128.93, 128.35, 121.90, 103.97, 103.04, 88.18, 82.65, 78.07, 73.34, 54.31, 42.35, 39.96, 25.65; ^{31}P NMR (162 MHz, CD_3OD) δ -10.55, -13.10; HRMS (ESI-TOF) calcd. for $C_{22}H_{26}N_4O_{12}P_2H$ [M+H]$^+$ 601.11; observed 601.1107.

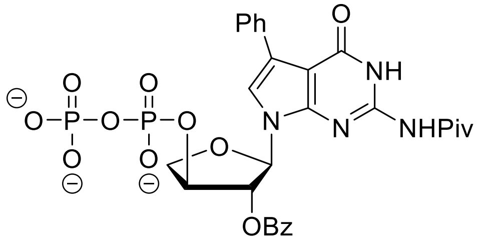

7-Deaza-7-Phenyl-(α-L-threofuranosyl) guanine 3′-diphosphate (11b):

Step 1: Prepared according to the procedure for **11a** with 156 mg (0.2 mmol, 1 equiv), 0.316 mL $(BnO)_2PN(^iPr)_2$ (1 mmol, 5 equiv), 5 mL dry DCM, 0.364 mL t-BuO_2H (5.5 M in $C_{10}H_{22}$, 2 mmol, 10 equiv);

20 mL DCM, 2x20 mL brine, 2x20 mL water for washing. Column chromatography (DCM/Acetone 1:1 to 1:9, 2% DIPEA added). Yield: 146 mg (0.148 mmol, 74%, DIPEA salt).

Step 2: Prepared according to the procedure for **11a** with 146 mg dibenzyl-protected diphosphate DIPEA salt (0.148 mmol, 1 equiv), 30 mg 10% Pd/C (0.2 mass equiv), 10 ml dry EtOH; 50 mL MeOH for washing. Yield: 155 mg (147 mmol, 99%, DIPEA salt, 73% two-step yield).

^{1}H NMR (400 MHz, CD_3OD) δ 8.02 (d, *J* = 7.4 Hz, 2H), 7.90 (d, *J* = 7.4 Hz, 2H), 7.61 (d, *J* = 14.5 Hz, 2H), 7.45 (t, *J* = 7.5 Hz, 3H), 7.32 (t, *J* = 7.6 Hz, 2H), 7.20 (t, *J* = 7.3 Hz, 1H), 6.39 (s, 1H), 5.79 (s, 1H), 5.25 (s, 1H), 4.57 (s, 1H), 4.31 (s, 1H), 1.22 (s, 9H); ^{13}C NMR (101 MHz, CD_3OD) δ 181.53, 165.41, 158.53, 149.79, 147.11, 133.59, 133.50, 129.56, 129.01, 128.35, 128.30, 127.79, 126.15, 122.10, 118.91, 101.34, 88.16, 46.19, 39.98, 29.38, 25.64, 7.81; ^{31}P NMR (162 MHz, CD_3OD) δ -9.67, -12.20; HRMS (ESI-TOF) calcd. for $C_{28}H_{29}N_4O_{12}P_2$ [M-H]$^-$ 675.1257; observed 675.1262.

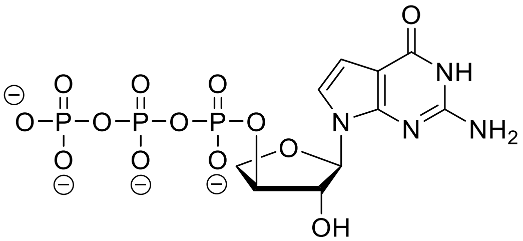

7-Deaza-(α-L-threofuranosyl) guanine 3′-triphosphate (1a): To a solution of **11a** (92 mg, 0.1261 mmol, 1 equiv) in 0.4 mL of anhydrous DCM was added $(BnO)_2PN(^{i}Pr)_2$ (48 µL, 0.1505 mmol, 1.2 equiv) and stirred the reaction for 3 h at room temperature under nitrogen atmosphere. After the starting material was consumed, t-BuO_2H (5.5 M in $C_{10}H_{22}$) (57 µL, 0.3152 mmol, 2.5 equiv) was added at 0°C and continued the reaction for another 30 min. The solution was then diluted with 10-20 times volume of DCM and the organic layer was sequentially washed with brine, and water (2 x 50 mL). The combined organic extracts were dried with Na_2SO_4 and evaporated under diminished pressure. The crude residue was purified by wet loading over silica gel with eluents MeOH/DCM (0%-5%-10%, containing 1% DIPEA). The fractions containing the product were collected and evaporated to afford as white solid (68 mg, 0.0687 mmol, 54%). To the above obtained solid (50 mg, 0.0508 mmol) in a round bottom flask was added of 10% Pd/C (20 mg, 0.4 mass equiv) and evacuated for 15-20 minutes and then added 2 mL of anhydrous MeOH. The reaction mixture was stirred in the presence of H_2 balloon for 12 h at room temperature. The resulting suspension was filtered through celite, washed with MeOH (100 mL) and concentrated under reduce pressure, dried under high vacuum to afford the product as white solid. The resulting solids were subsequently dissolved in 30-33% aqueous NH_4OH (5.0 mL) and stirred for 18 h at 37°C in a sealed tube. After concentrating the solution under reduced pressure in rotavapor, the resulting solid was resuspended in minimum volume of MilliQ water (5 mL) and the solution extracted with DCM (20 mL). The aqueous layer was concentrated under diminished pressure. To the concentrated aqueous extract was added $NaClO_4$ (92 mg, 0.762 mmol, 15 equiv) in acetone (100 mL) drop wise at room temperature. The resulting suspension was centrifuged at 4400 rpm for 5 min at room temperature, the supernatant discarded, and the pellet washed with organic solution (10:1 acetone/DCM, 20 mL), and then dried under

vacuum to afford the desired product **1a** as white solid (29 mg, 0.0503 mmol, 53% yield over three steps, Na salt).

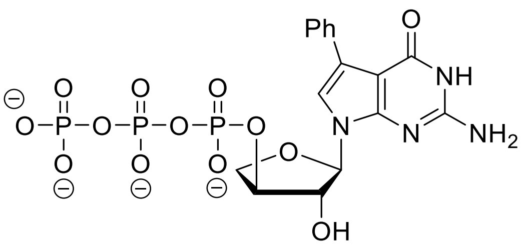

7-Deaza-(α-L-threofuranosyl) guanine 3′-triphosphate (1b):

Step 1: Prepared according to the procedure for **1a** with 130 mg **11b** (0.122 mmol), 5 mL dry DCM, 0.2 mL $(BnO)_2PN(^iPr)_2$ (0.61 mmol, 5 equiv), 0.223 mL t-BuO_2H (5.5 M in $C_{10}H_{22}$, 1.22 mmol, 10 equiv); 20 mL DCM, 2x20 mL brine and water for washing. Column chromatography (DCM/MeOH from 100:0 to 4:1, 2% DIPEA added). Yield: 67 mg (0.056 mmol, 46%, DIPEA salt)

Step 2: Prepared according to the procedure for **1a** with 67 mg of the above obtained solid (0.056 mmol, 1 equiv), 14 mg 10% Pd/C (0.2 mass equiv), 5 mL dry EtOH; 40 mL MeOH for washing. Obtained solid was used in the next step without measuring the yield.

Step 3: Prepared according to the procedure for **1a** with the above **obtained** solid, 5 mL 30-33% aqueous NH_4OH; 5 ml MilliQ water, 20 mL DCM for first washing; 100 mL acetone containing 103 mg $NaClO_4$ (0.84 mmol, 15 equiv) for precipitation; 20 mL Acetone/DCM 10:1 for second washing. Yield: 35 mg (0.053 mmol, 44% over three steps, Na salt).

Phosphoramidite Synthesis

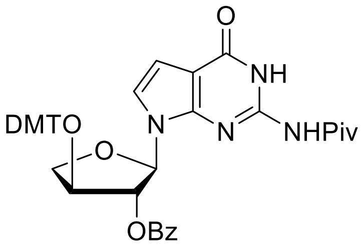

7-Deaza-N^2-pivaloyl-9-(2′-*O*-benzoyl-3′-*O*-dimethoxytrityl-α-L-threofuranosyl) guanine (12a): To a solution of **2a** (200 mg, 0.4541 mmol, 1 equiv) in DCM (5 mL), collidine (0.3 mL, 2.2705 mmol, 5 equiv) and 4,4-dimethoxytritylchloride (185 mg, 0.5449 mmol, 1.2 equiv) were added followed by the addition of $AgNO_3$ (66 mg, 0.3884 mmol, 0.85 equiv). The reaction mixture was stirred at 37°C for overnight, quenched with water and diluted with DCM. The organic layer was separated, dried and concentrated under reduced pressure to give crude. The crude was purified by column chromatography (on TEA-deactivated silica, eluted with 2:10 EtOAc:Hex) to give product (215 mg, 0.2894 mmol, 64%) as white solid.

R_f 0.6 (TLC EtOAc/Hex 1:1); ^{1}H NMR (400 MHz, $CDCl_3$) δ 11.74 (s, 1H), 7.97 – 7.90 (m, 3H), 7.63 – 7.57 (m, 1H), 7.48 – 7.42 (m, 4H), 7.39 – 7.34 (m, 4H), 7.32 – 7.25 (m, 3H), 7.20 (t, *J* = 7.2 Hz, 1H), 6.77 (t, *J* = 8.8 Hz, 4H), 6.70 (dd, *J* = 3.5, 0.8 Hz, 1H), 6.04 (d, *J* = 1.6 Hz, 1H), 5.30 (t, *J* = 1.6 Hz, 1H), 4.58 (ddd,

J = 6.2, 4.5, 1.7 Hz, 1H), 3.91 (dd, J = 10.1, 6.1 Hz, 1H), 3.85 (dd, J = 10.1, 4.6 Hz, 1H), 3.69 (dd, J = 5.9, 0.8 Hz, 6H), 1.25 (s, 9H); ^{13}C NMR (101 MHz, $CDCl_3$) δ 179.38, 164.96, 158.96, 158.92, 157.74, 147.57, 146.06, 144.69, 135.73, 135.52, 133.63, 130.16, 130.06, 129.89, 129.12, 128.55, 128.27, 127.92, 127.25, 120.55, 113.63, 113.59, 105.61, 104.03, 88.27, 87.69, 82.40, 77.52, 73.08, 55.22, 55.20, 40.11, 27.06; HRMS (ESI-TOF) calcd. for $C_{43}H_{42}N_4O_8Na$ $[M+Na]^+$ 765.2900, observed 765.2901.

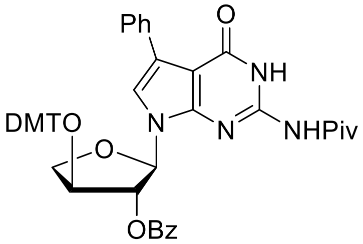

7-Deaza-7-Phenyl-N^2-pivaloyl-9-(2′-*O*-benzoyl-3′-*O*-dimethoxytrityl-α-L-threofuranosyl) guanine (12b): Prepared according to the procedure for **12a** with 500 mg **2b** (0.98 mmol, 1 equiv), 1.65 g 4,4-dimethoxytritylchloride (5 equiv), 0.65 mL collidine (5 equiv), 0.57 mL lutidine (5 equiv), 88 mg $AgNO_3$ (5 equiv). Column chromatography (EtOAc/Hex 1:9 to 1:1, 2% TEA added). Yield: 706 mg (0.8621 mmol, 88%). R_f 0.2 (TLC EtOAc/Hexane 3:7 (1% TEA)); ^{1}H NMR (400 MHz, Acetone-d_6) δ 11.91 (s, 1H), 9.67 (s, 1H), 7.98 – 7.93 (m, 4H), 7.77 (s, 1H), 7.69 – 7.63 (m, 1H), 7.61 – 7.56 (m, 2H), 7.48 (dt, J = 23.3, 8.1 Hz, 6H), 7.31 – 7.18 (m, 6H), 6.83 – 6.74 (m, 4H), 6.06 – 6.01 (m, 1H), 4.97 (d, J = 1.3 Hz, 1H), 4.73 (t, J = 5.1 Hz, 1H), 4.25 – 4.16 (m, 2H), 3.64 (d, J = 5.7 Hz, 6H), 1.28 (s, 9H); ^{13}C NMR (101 MHz, Acetone-d_6) δ 205.29, 181.01, 164.65, 159.16, 159.12, 157.12, 149.43, 147.52, 145.24, 135.90, 135.35, 133.78, 133.72, 130.28, 130.12, 129.75, 129.10, 128.70, 128.24, 128.13, 128.03, 127.83, 127.05, 126.25, 122.03, 118.15, 113.60, 113.52, 101.76, 88.42, 87.40, 82.55, 77.68, 73.11, 54.59, 54.57, 40.15, 26.08; HRMS (ESI-TOF) calcd. for $C_{49}H_{46}N_4O_8Na$ $[M+Na]^+$ 841.3214, observed 841.3192.

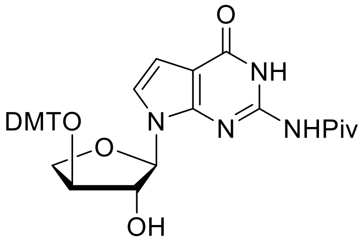

7-Deaza-N^2-pivaloyl-9-(3′-*O*-dimethoxytrityl-α-L-threofuranosyl) guanine (13a): To a solution of **12a** (200 mg, 0.2692 mmol, 1 equiv) in MeOH (15 mL), sodium methoxide (726 mg, 1.346 mmol, 5 equiv) was added at 0°C. After stirring the reaction mixture for 5 h at 0°C, saturated aqueous NH_4Cl solution (50 mL) was added and the reaction mixture was concentrated under reduced pressure to remove organics. The residue was extracted with EtOAc (2 x 100mL), and the combined organic layer was dried over Na_2SO_4 and concentrated under reduced pressure to give product (170 mg, 0.2661 mmol, 98%) as white solid.

R_f 0.3 (TLC EtOAc:Hex 1:1); ^{1}H NMR (400 MHz, $CDCl_3$) δ 11.80 (s, 1H), 8.65 (s, 1H), 7.48 (d, J = 7.7 Hz, 2H), 7.37 (t, J = 9.6 Hz, 4H), 7.23 (dt, J = 28.7, 7.5 Hz, 4H), 7.05 (d, J = 3.6 Hz, 1H), 6.81 (d, J = 8.4 Hz, 4H), 6.48 (d, J = 3.6 Hz, 1H), 5.87 (d, J = 3.0 Hz, 1H), 4.38 (t, J = 3.1 Hz, 1H), 4.29 (q, J = 4.1 Hz, 1H),

3.75 (d, J = 2.4 Hz, 6H), 3.50 (t, J = 4.0 Hz, 2H), 1.24 (s, 9H); ^{13}C NMR (101 MHz, $CDCl_3$) δ 179.98, 158.84, 158.82, 158.01, 147.36, 146.01, 145.04, 136.30, 136.14, 130.23, 129.63, 128.43, 128.25, 128.09, 127.14, 120.42, 113.44, 105.03, 103.13, 89.78, 87.54, 81.47, 78.92, 72.87, 55.29, 40.21, 26.97; HRMS (ESI-TOF) calcd. for $C_{36}H_{38}N_4O_7Na$ $[M+Na]^+$ 661.2638, observed 661.2631.

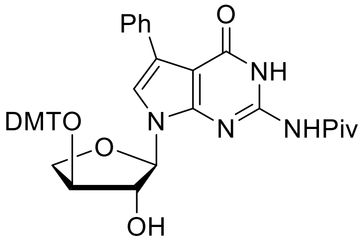

7-Deaza-7-Phenyl-N^2-pivaloyl-9-(3′-*O*-dimethoxytrityl-α-L-threofuranosyl) guanine (13b): Prepared according to the procedure for **13a** with 280 mg **12b** (0.34 mmol, 1 equiv), 1.7 mL 1N aq. NaOH (5 equiv), 20 mL of THF/MeOH/water (5:4:1) as a reaction solvent, 50 mL saturated aqueous NH_4Cl solution, 2 x 100 mL EtOAc for extraction. Column chromatography (EtOAc:Hexane from 1:9 to 7:3, 1% TEA added). Yield: 160 mg (0.2238 mmol, 66%).

R_f 0.2 (TLC EtOAc/Hex 6:4 (1% TEA)); ^{1}H NMR (400 MHz, CD_3OD) δ 7.74 – 7.69 (m, 2H), 7.52 (s, 1H), 7.39 – 7.35 (m, 2H), 7.26 – 7.21 (m, 6H), 7.20 – 7.12 (m, 4H), 6.71 (dq, J = 8.8, 3.3 Hz, 4H), 5.95 (d, J = 1.2 Hz, 1H), 4.21 – 4.17 (m, 1H), 3.90 (d, J = 3.1 Hz, 2H), 3.69 (s, 6H), 3.65 (t, J = 1.2 Hz, 1H), 1.28 (s, 9H); ^{13}C NMR (101 MHz, CD_3OD) δ 181.33, 158.93, 158.89, 158.55, 148.67, 146.66, 144.80, 136.10, 135.81, 133.70, 129.97, 129.82, 127.98, 127.94, 127.72, 127.70, 126.62, 125.91, 120.71, 118.98, 113.01, 112.99, 101.55, 90.80, 87.78, 79.97, 78.90, 74.27, 54.35, 46.99, 39.98, 25.71; HRMS (ESI-TOF) calcd. for $C_{42}H_{42}N_4O_7Na$ $[M+Na]^+$ 737.2951, observed 737.2936.

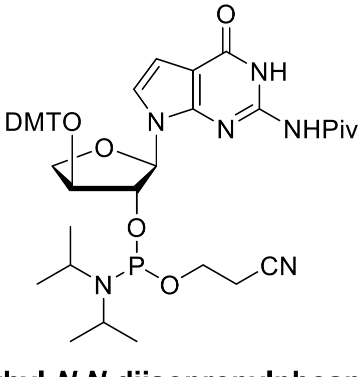

7-Deaza-N^2-pivaloyl-9-(2′-*O*-cyanoethyl-*N,N*-diisopropylphosphoramidite-3′-*O*-dimethoxytrityl-α-L-threofuranosyl) guanine (14a): To a suspension of **13a** (160 mg, 0.2505 mmol, 1 equiv) and DMAP (6 mg, 0.0501 mmol, 0.2 equiv) in DCM (2.5 mL) was added DIPEA (95 µL, 0.5455 mmol, 2.2 equiv), followed by the addition of 2-cyanoethyl-*N,N*-diisopropylchlorophosphoramidite (84 µL, 0.3762 mmol, 1.5 equiv). After stirring for 30 min at room temperature, the reaction mixture was diluted with DCM (20 mL) and washed with saturated aqueous $NaHCO_3$ (40 mL). The organic layer was washed with brine, dried and concentrated under reduced pressure to give crude. The crude was purified by column chromatography on silica gel (eluted with 1.5:10 EtOAc:Hex 1% TEA) to afford product **14a** (140 mg, 0.1668 mmol, 66%) as white solid.

R_f 0.8 (TLC EtOAc:Hexane 3:10); ^{31}P NMR (162 MHz, CD_3CN) δ 150.67, 150.35; HRMS (ESI-TOF) calcd. for $C_{45}H_{55}N_6O_8PNa$ $[M+Na]^+$ 861.3716, observed 861.3723.

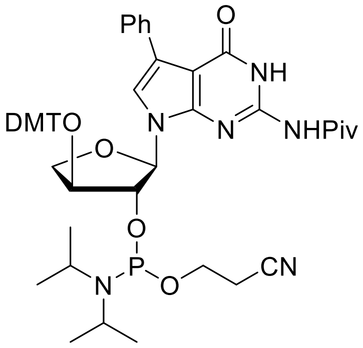

7-Deaza-N^2-pivaloyl-9-(2′-*O*-cyanoethyl-*N,N*-diisopropylphosphoramidite-3′-*O*-dimethoxytrityl-α-L-threofuranosyl) guanine (14b):

Prepared according to the procedure for **14a** with 230 mg **13b** (0.32 mmol, 1 equiv), 10 mg DMAP (0.2 equiv), 10 mL dry DCM, 0.11 mL DIPEA (2 equiv) and 0.13 mL 2-cyanoethyl-*N*,*N*-diisopropylchlorophosphoramidite (1.8 equiv). Column chromatography (EtOAc:Hex from 1:4 to 9:1, 1% TEA added). Yield: 120 mg (0.1311 mmol, 41%).

R_f 0.9 (TLC EtOAc/Hex 6:4 (1% TEA)). ^{31}P NMR (162 MHz, CD_3OD) δ 151.48, 150.13. HRMS (ESI-TOF) calcd. for $C_{51}H_{59}N_6O_8PNa$ $[M+Na]^+$ 915.4210, observed 915.4205.

References

1. J. Ludwig, A New Route to Nucleoside 5′-Triphosphates, *Acta. Biochim. et Biophys. Acad. Sci. Hung*. 1981, **16**, 131–133.
2. J. Ludwig and F. Eckstein, Rapid and Efficient Synthesis of Nucleoside 50-0-(1-Thiotriphosphates), 50-Triphosphates and 20,30-Cyclophosphorothioates using 2-Chloro-4H-1,3,2-benzodioxaphosphorin-4-one, *J. Org. Chem.*, 1989, **54**, 631–635.
3. K. Zou, A. Horhota, B. Yu, J. W. Szostak, L. W. McLaughlin, Synthesis of α-L-Threofuranosyl Nucleoside Triphosphates (tNTPs), *Org. Lett*. 2005, **7**, 1485–1487.
4. S. Zhang, H. Yu, J. C. Chaput, Synthesis of Threose Nucleic Acid (TNA)Triphosphates and Oligonucleotides by Polymerase-Mediated Primer Extension, *Curr. Protoc. Nucleic Acid Chem.*, 2013 4:5.54:4.54.1–4.54.17.
5. M. Yoshikawa, T. Kato, T. KTakenishi, A Novel Method for Phosphorylation of Nucleosides to 5′-Nucleotides, Tetrahedron Lett., 1967, 50, 5065–5068. Nucleotides, Tetrahedron Lett., 1967, 50, 5065–5068.
6. S. P. Sau, J. C. Chaput, A One-pot Synthesis of α-L-Threofuranosyl Nucleoside Triphosphates (tNTPs), *Bioorg. Med. Chem. Lett*. 2016, **26**, 3271–3273.
7. D. E. Hoard and D. G. Ott, Conversion of Mono- and Oligodeoxyribonucleotides to 5'-Triphosphates, *J. Am. Chem. Soc.*, 1964, **87**, 8, 1785–1788.

8. S. Bala, J. Y. Liao, H. Mei, J. C. Chaput, Synthesis of α-lThreofuranosyl Nucleoside 3′-Monophosphates, 3′-Phosphoro(2-Methyl)imidazolides, and 3′-Triphosphates, *J. Org. Chem.* 2017, **82**, 5910−5916.
9. H. Mei, J. C. Chaput, Expanding the Chemical Diversity of TNA with tUTP Derivatives that are Substrates for a TNA Polymerase, *Chem. Commun.*, 2017, **54**, 1237–1240.
10. J.-Y. Liao, S. Bala, A. K. Ngor, E. J. Yik, J. C. Chaput, P(V) Reagents for the Scalable Synthesis of Natural and Modified Nucleoside Triphosphates, *J. Am. Chem. Soc.*, 2019, **141**, 13286−13289.
11. G. S. Cremosnik, A. Hofer, H. J. Jessen, Iterative Synthesis of Nucleoside Oligophosphates with Phosphoramidites, *Angew. Chem. Int. Ed.*, 2014, **53**, 286−289.
12. S. P. Sau, J. C. Chaput, A Gram-cale HPLC-Free Synthesis of TNA Triphosphates Using an Iterative Phosphorylation Strategy, *Org. Lett.* 2017, 19, 16, 4379–4382.

NMR Spectra

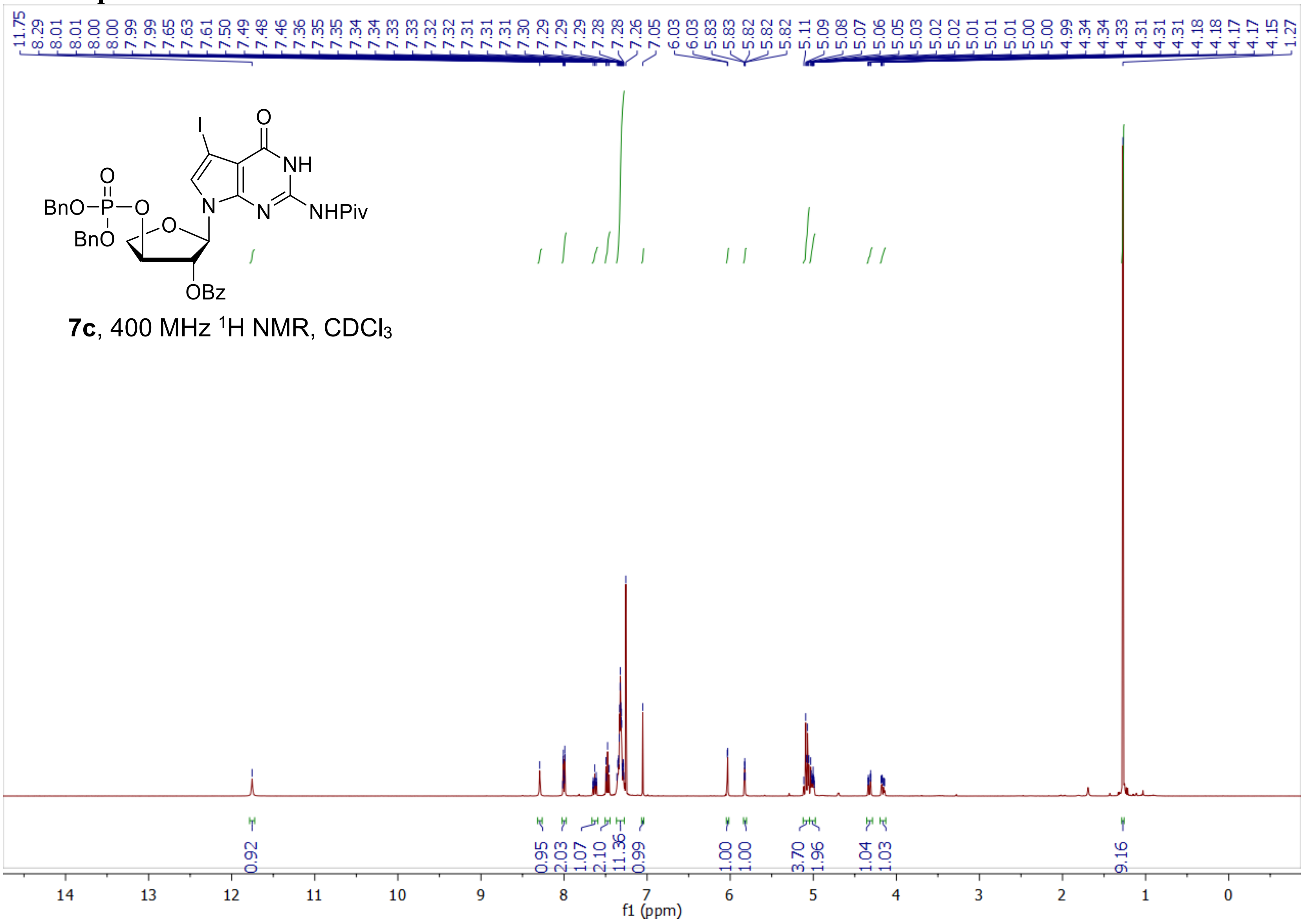

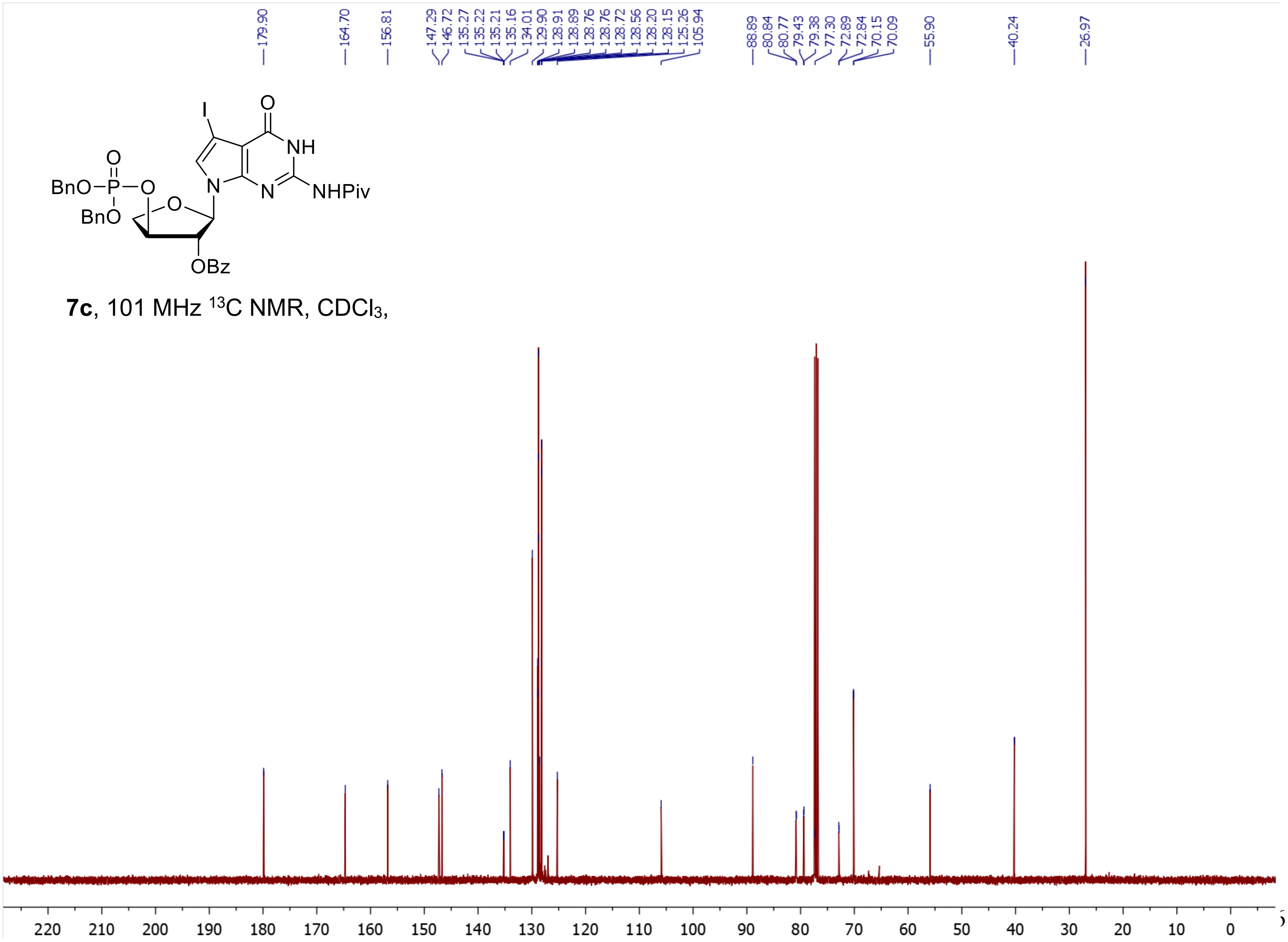
179.90
164.70
156.81
147.29
146.72
135.27
135.22
135.21
135.16
134.01
129.90
128.91
128.89
128.76
128.76
128.72
128.56
128.20
128.15
125.26
105.94
88.89
80.84
80.77
79.43
79.38
77.30
72.89
72.84
70.15
70.09
55.90
40.24
26.97
I
O
NH
N
N
NHPiv
O
BnO
P
O
BnO
O
OBz
7c, 101 MHz ^{13}C NMR, $CDCl_3$,
220
210
200
190
180
170
160
150
140
130
120
110
100
90
80
70
60
50
40
30
20
10
0

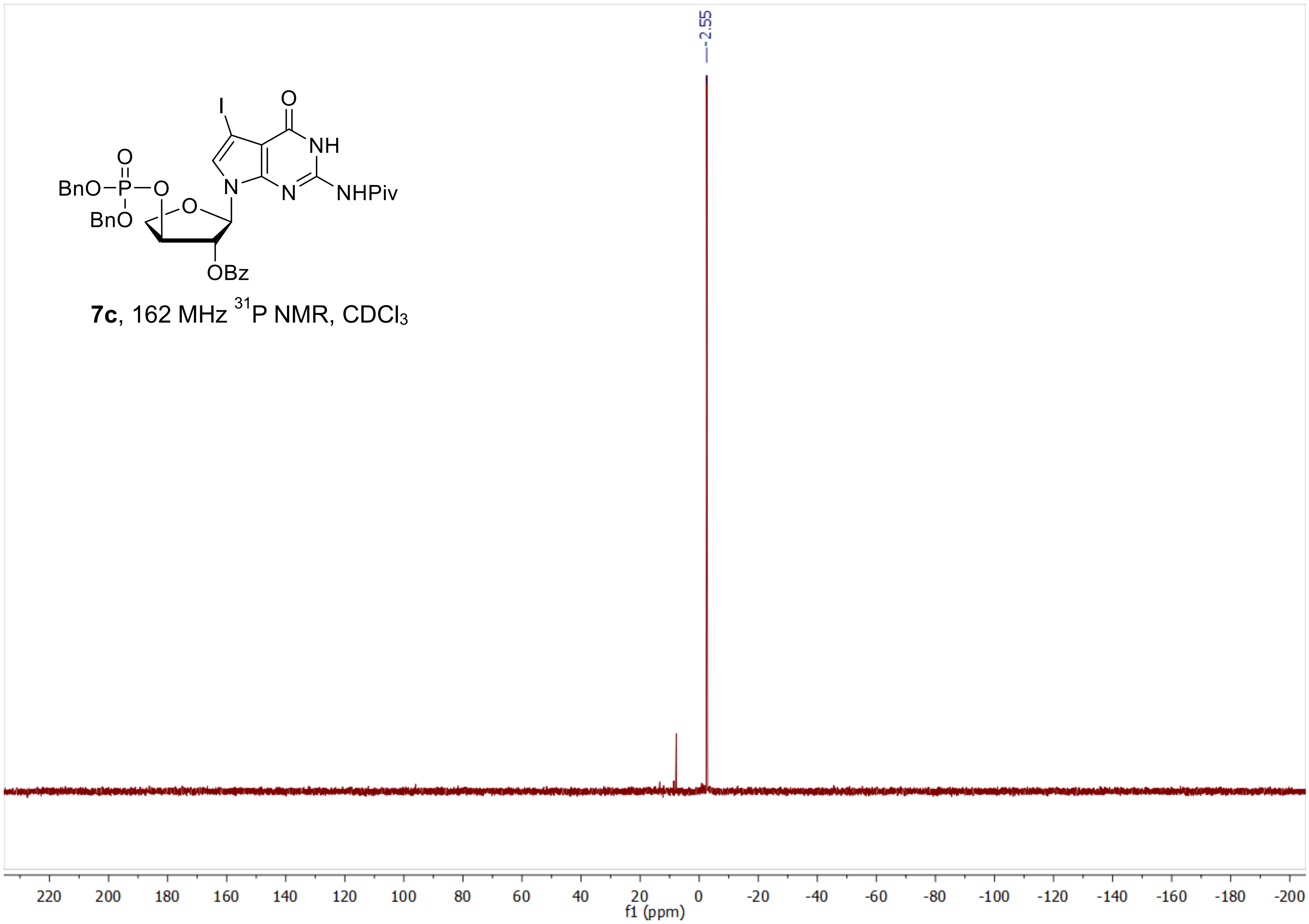
-2.55
7c, 162 MHz ^{31}P NMR, $CDCl_3$
220 200 180 160 140 120 100 80 60 40 20 0 -20 -40 -60 -80 -100 -120 -140 -160 -180 -200
f1 (ppm)

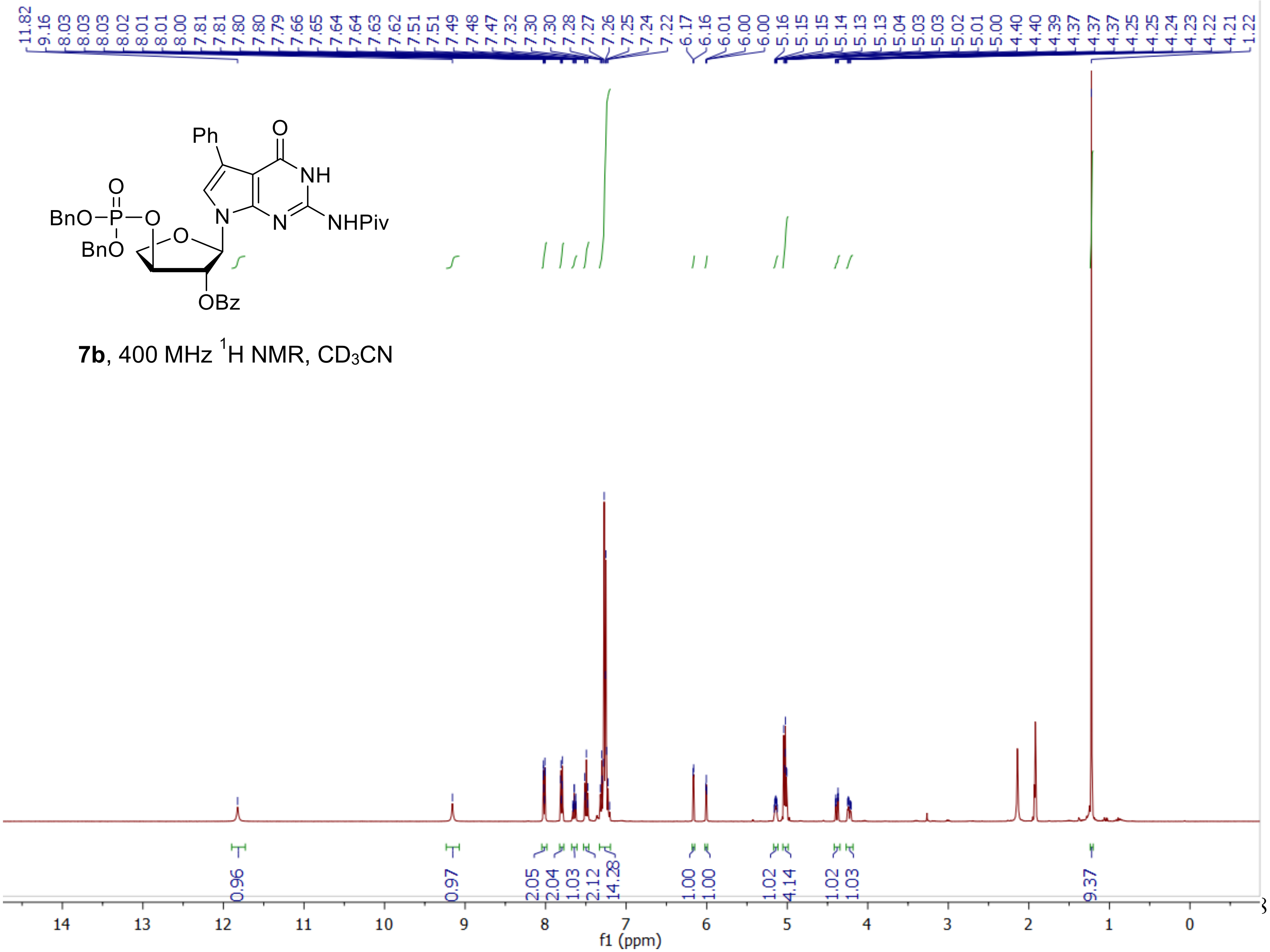

7b, 400 MHz ^{1}H NMR, CD_3CN

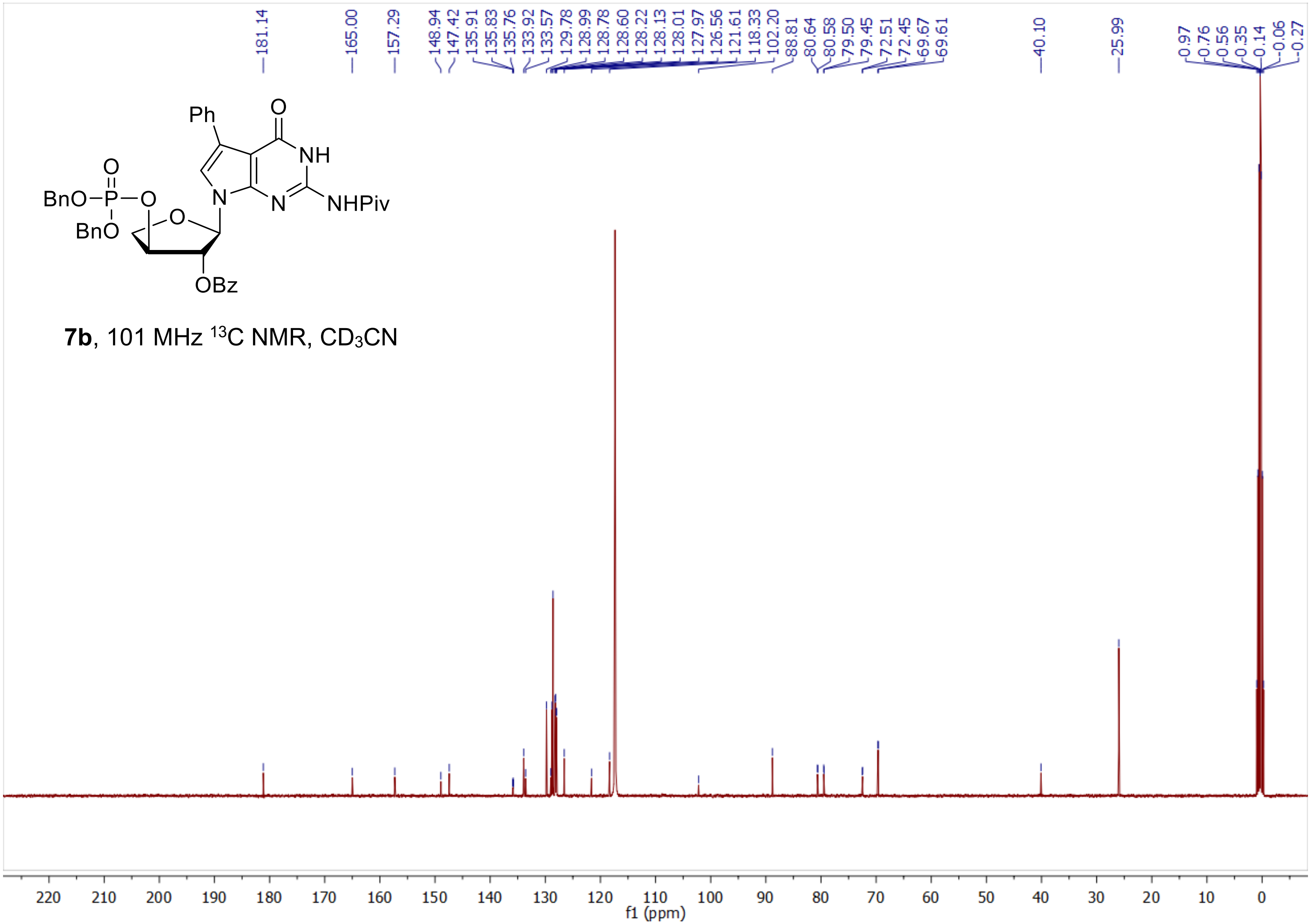

7b, 101 MHz ^{13}C NMR, CD_3CN

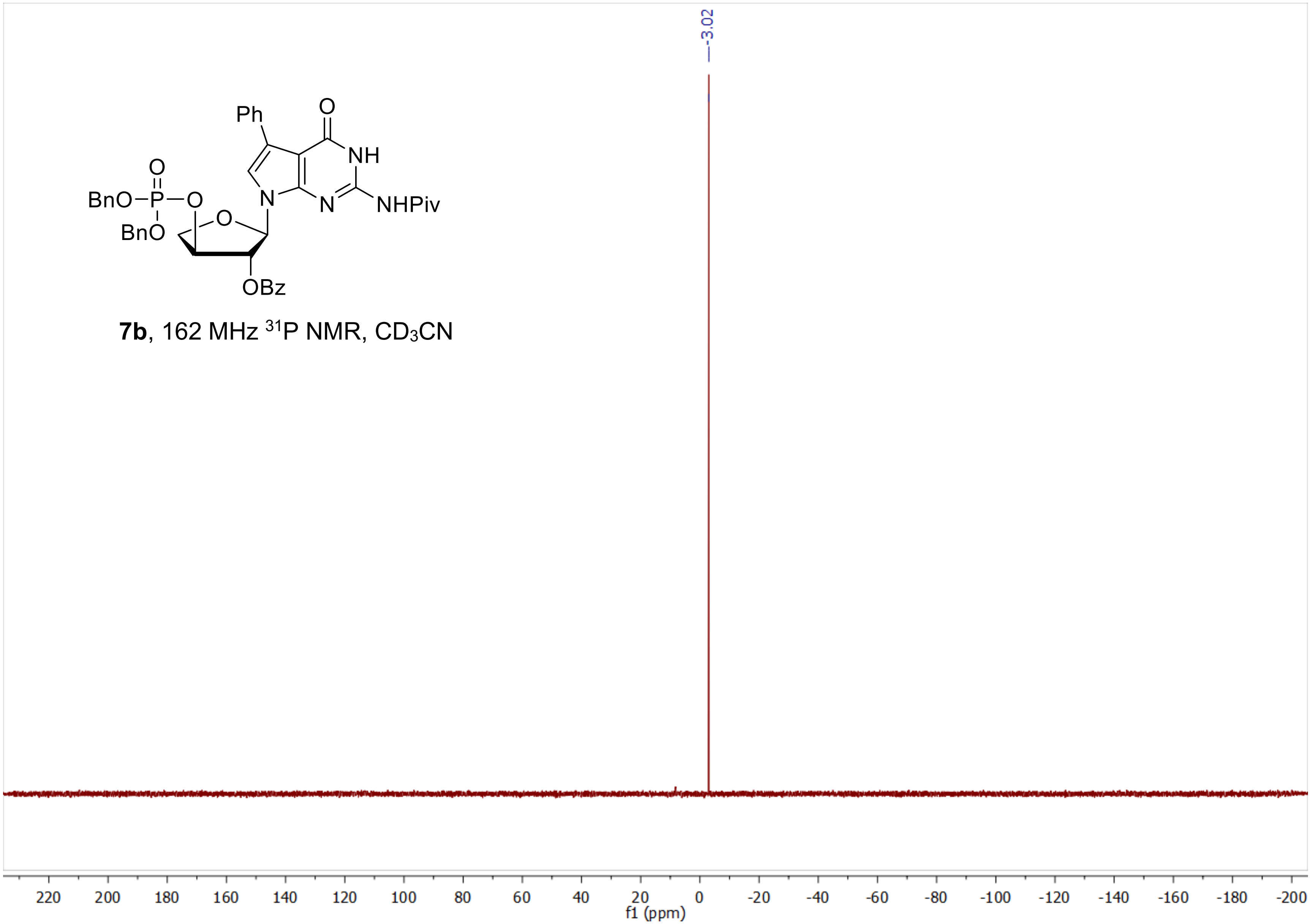

7b, 162 MHz ^{31}P NMR, CD_3CN

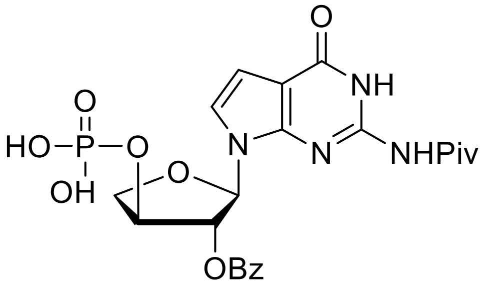

8a, 400 MHz ^{1}H NMR, CD_3OD

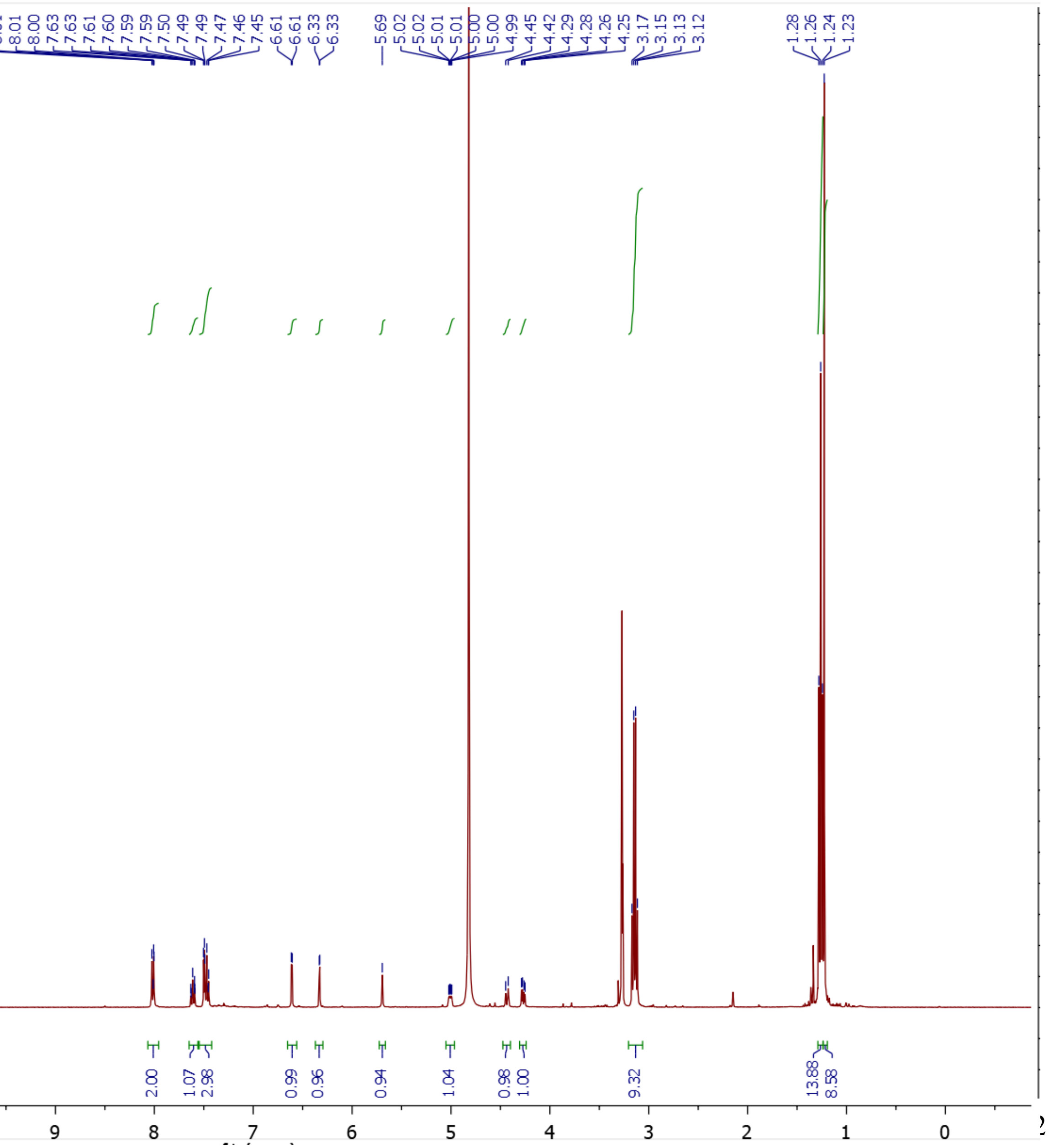

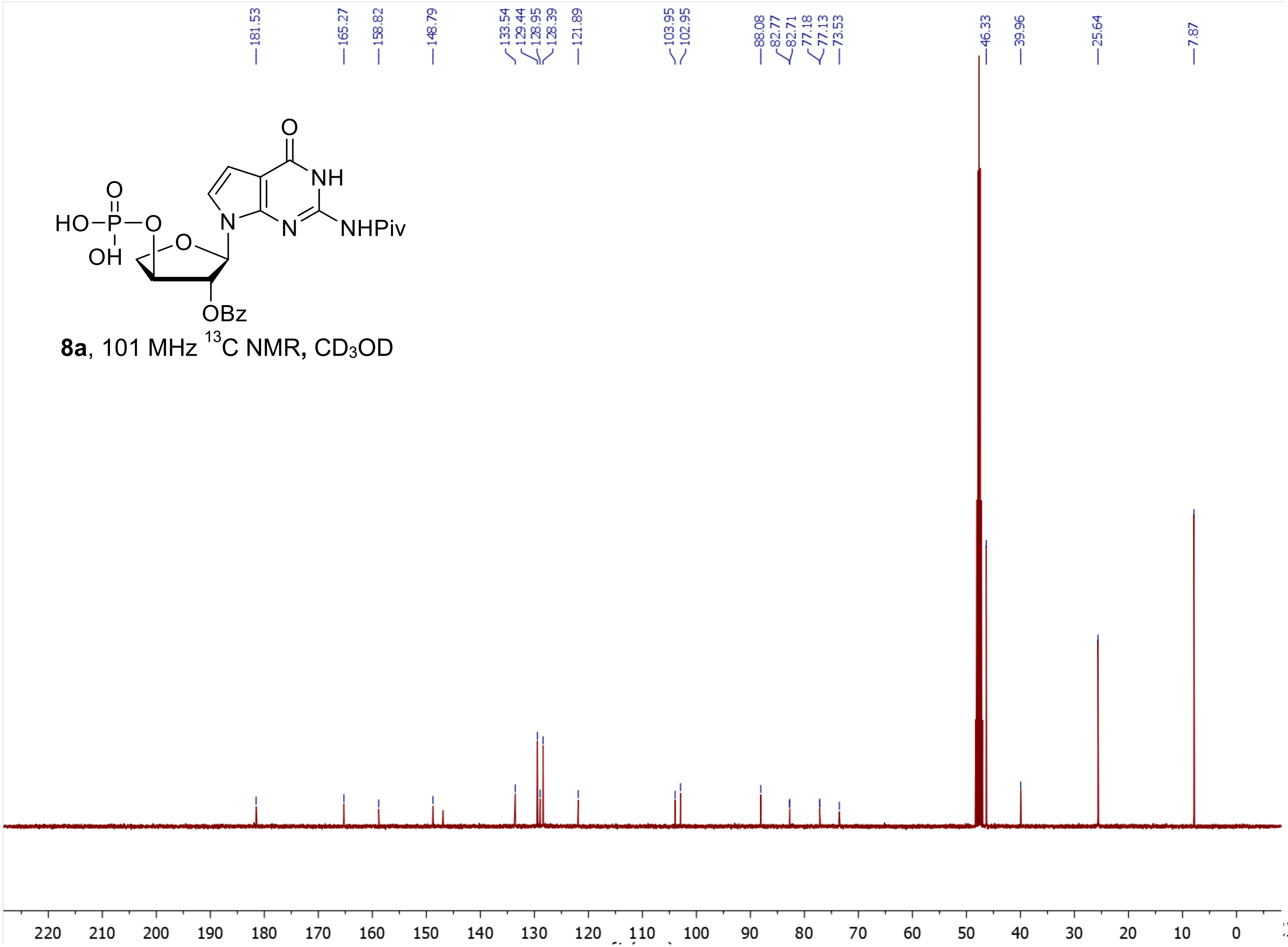

8a, 101 MHz ^{13}C NMR, CD_3OD

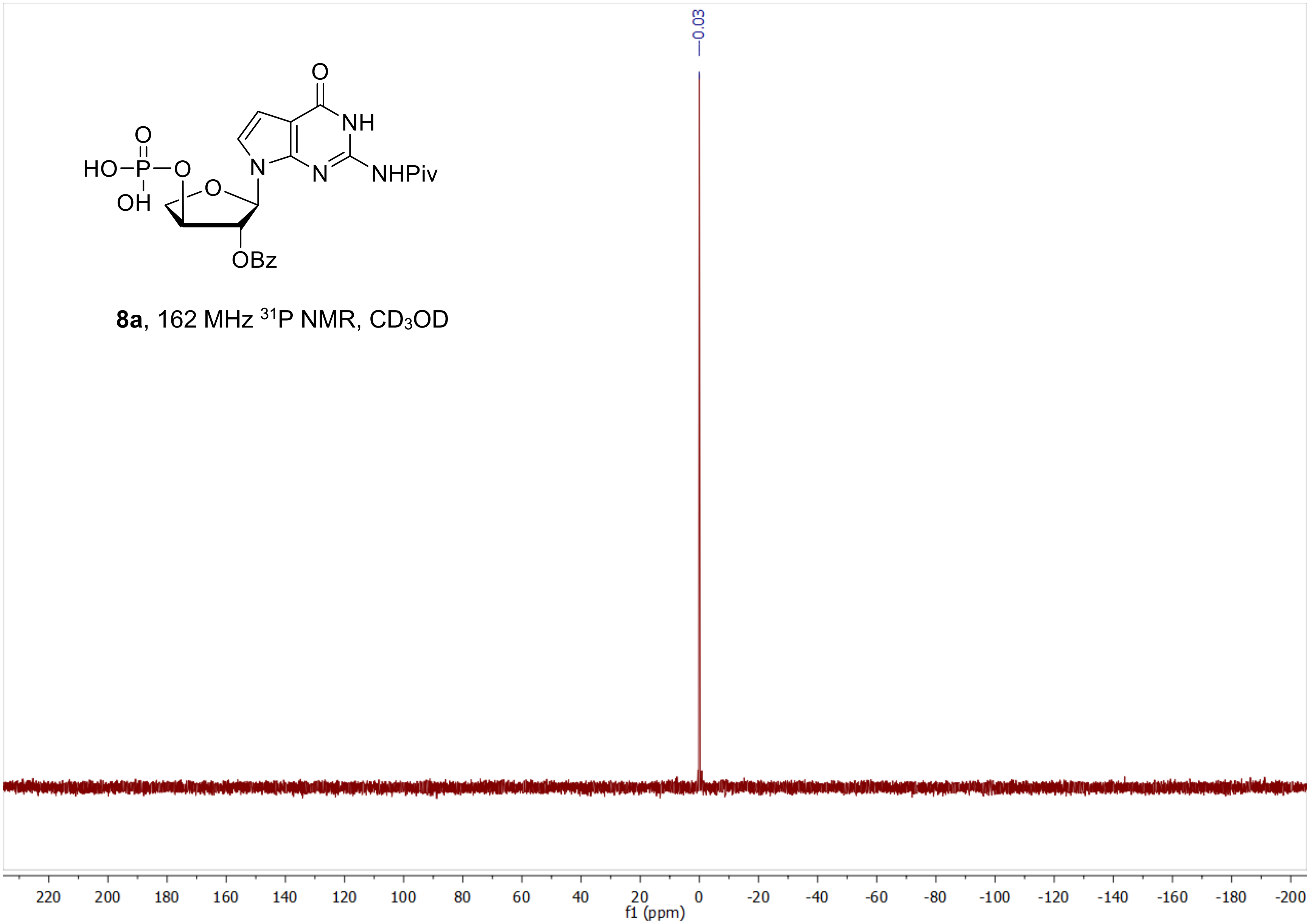

8a, 162 MHz ^{31}P NMR, CD_3OD

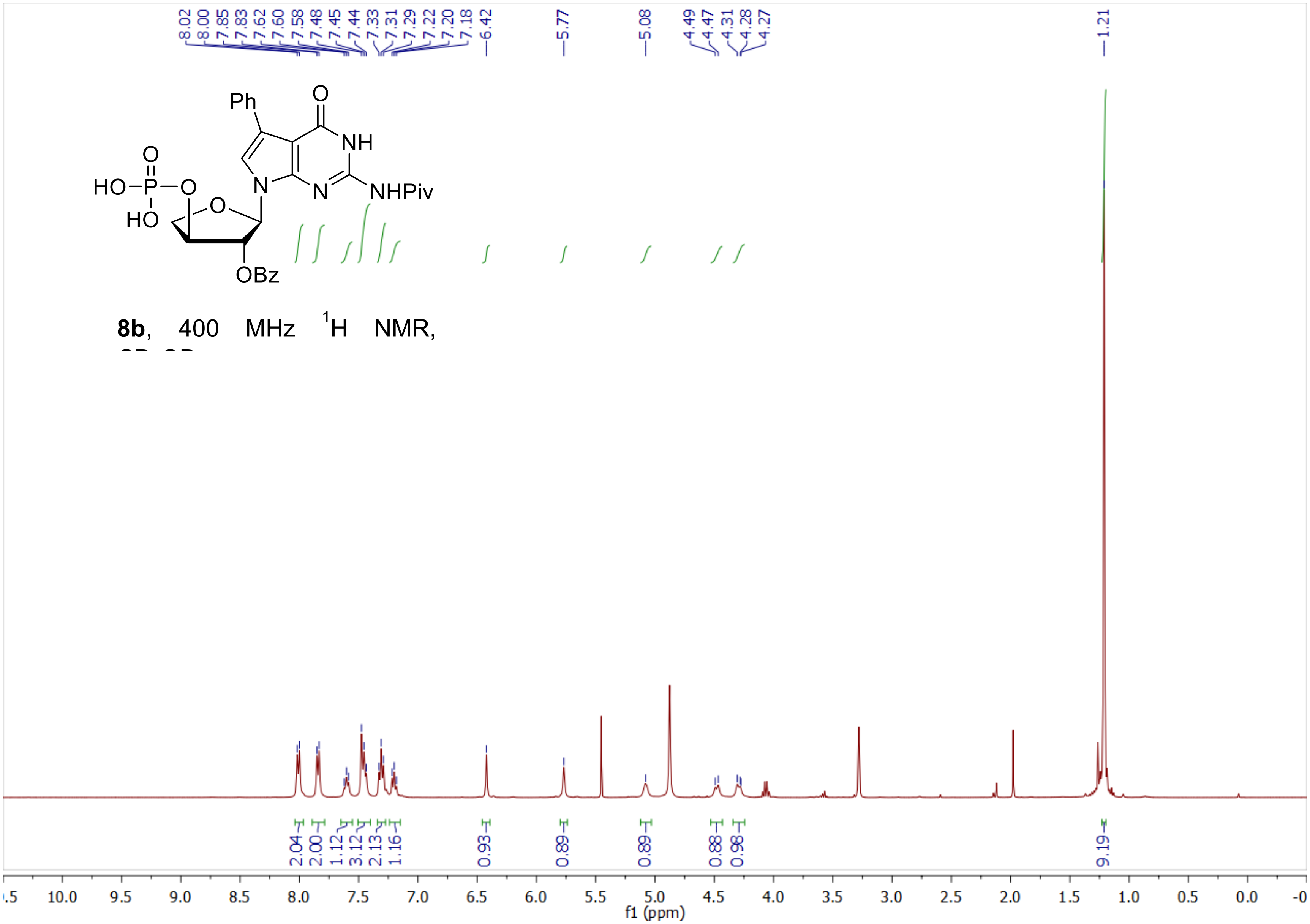

8b, 400 MHz ^{1}H NMR,

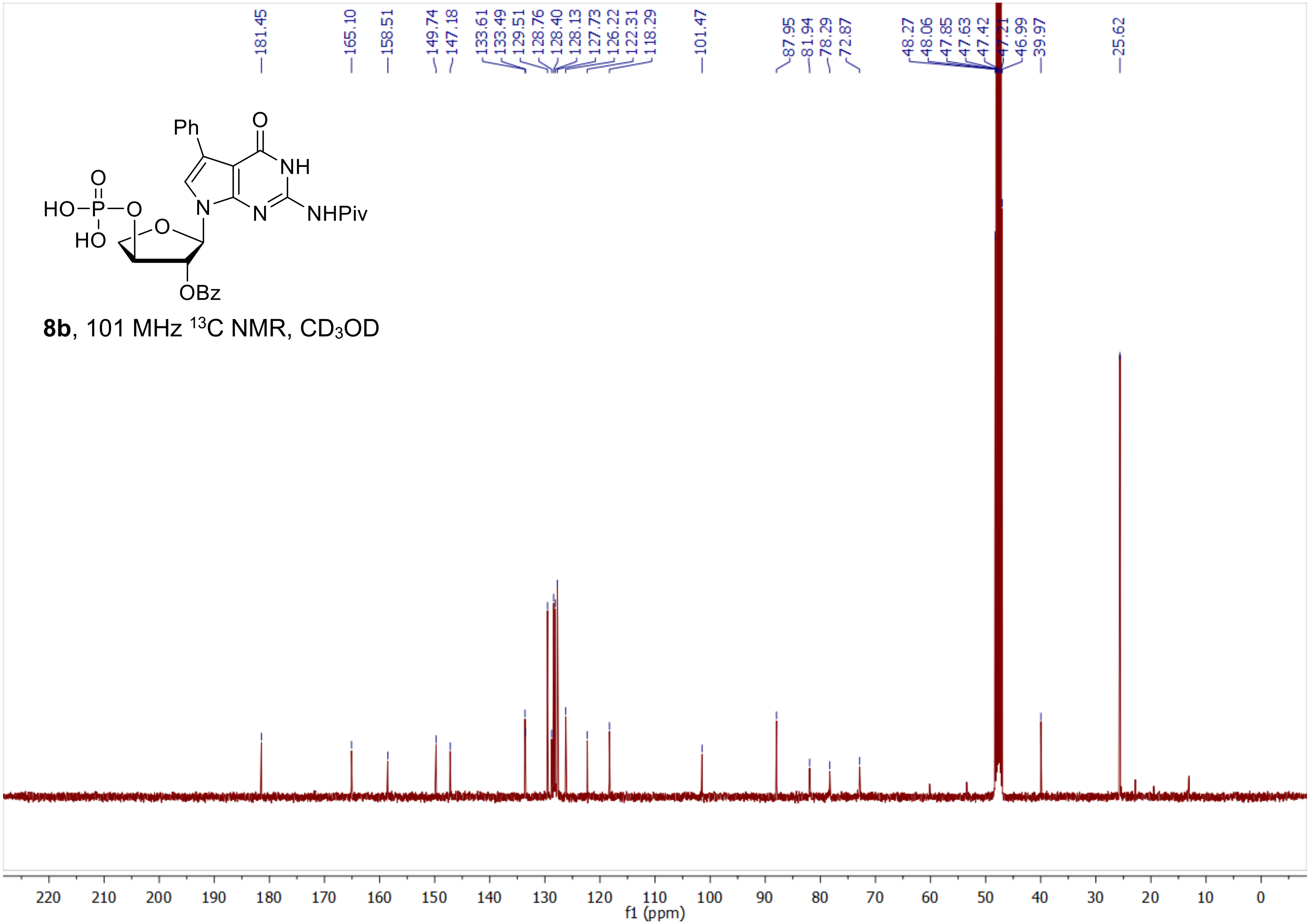

8b, 101 MHz ^{13}C NMR, CD_3OD

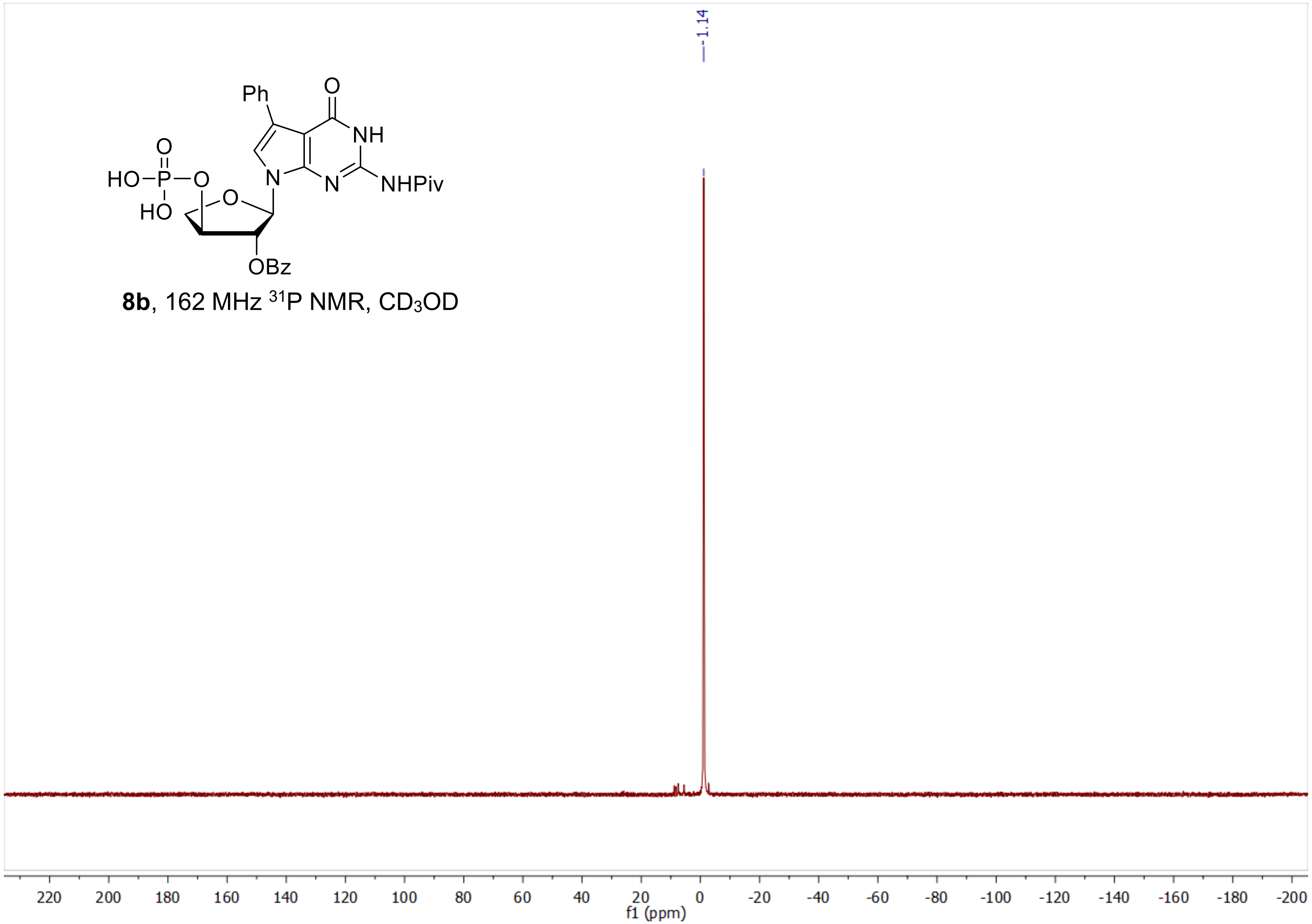

8b, 162 MHz ^{31}P NMR, CD_3OD

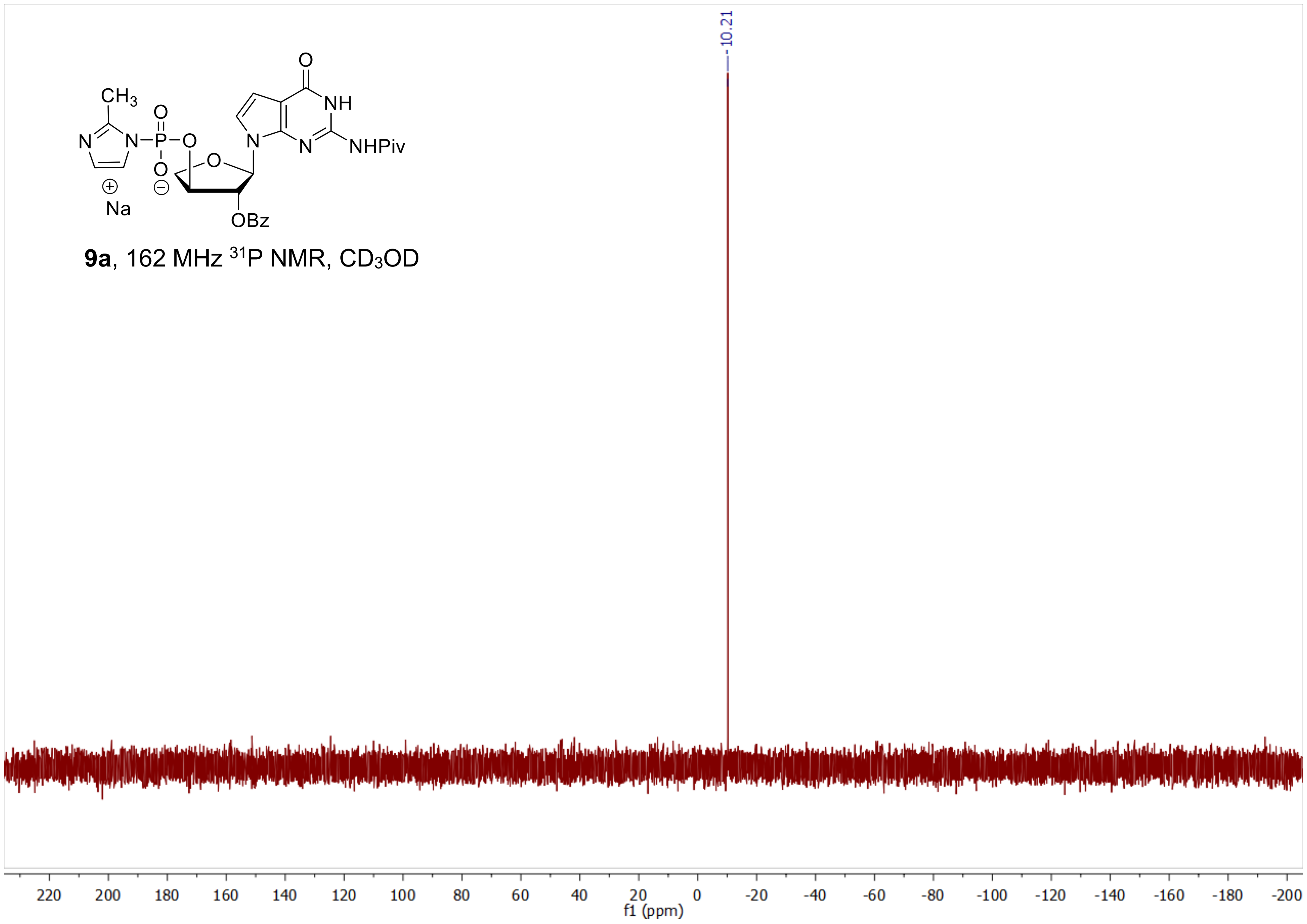

9a, 162 MHz ^{31}P NMR, CD_3OD

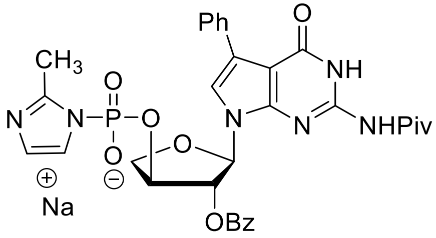

9b, 162 MHz ^{31}P NMR, $CDCl_3$

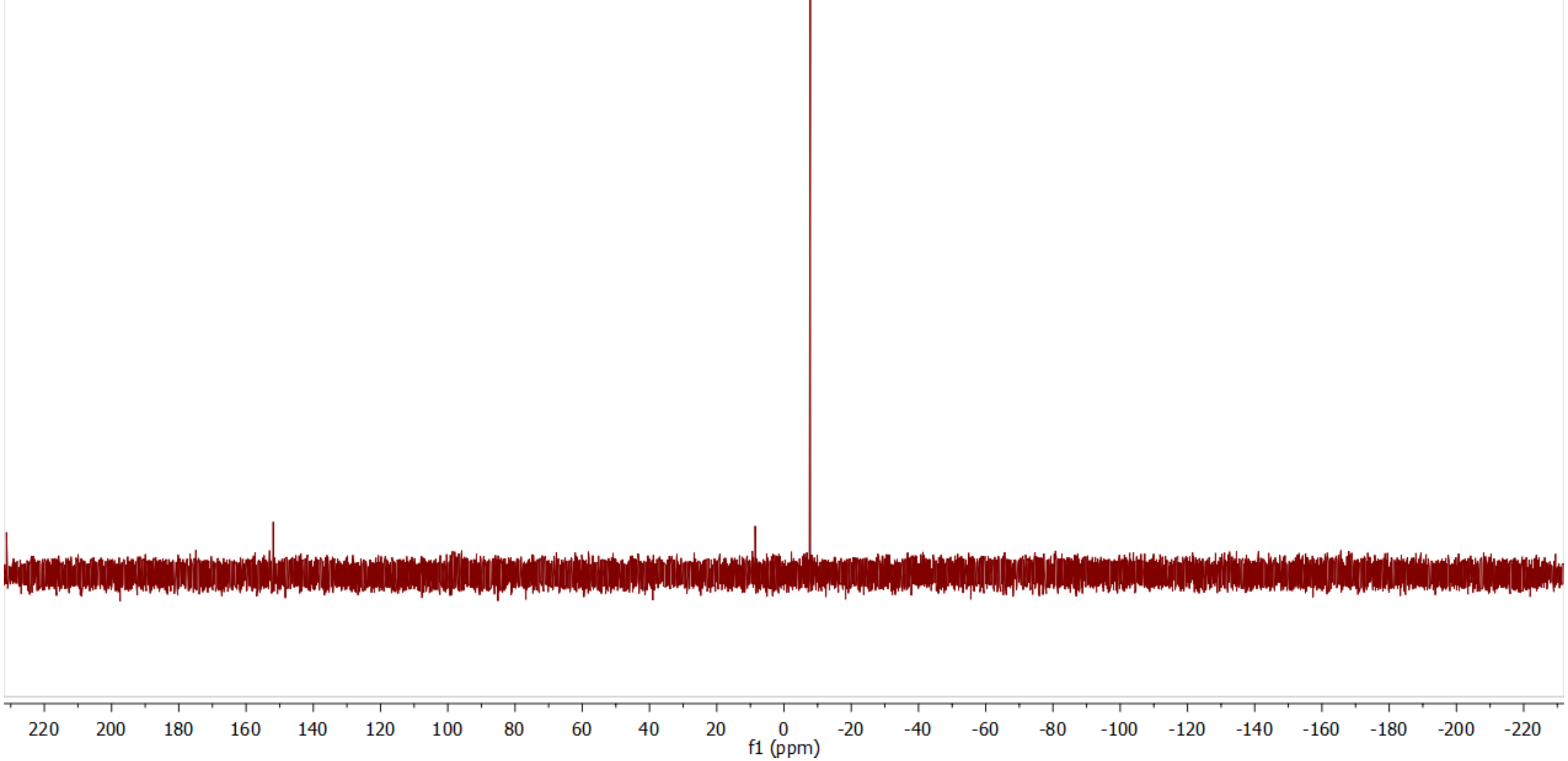

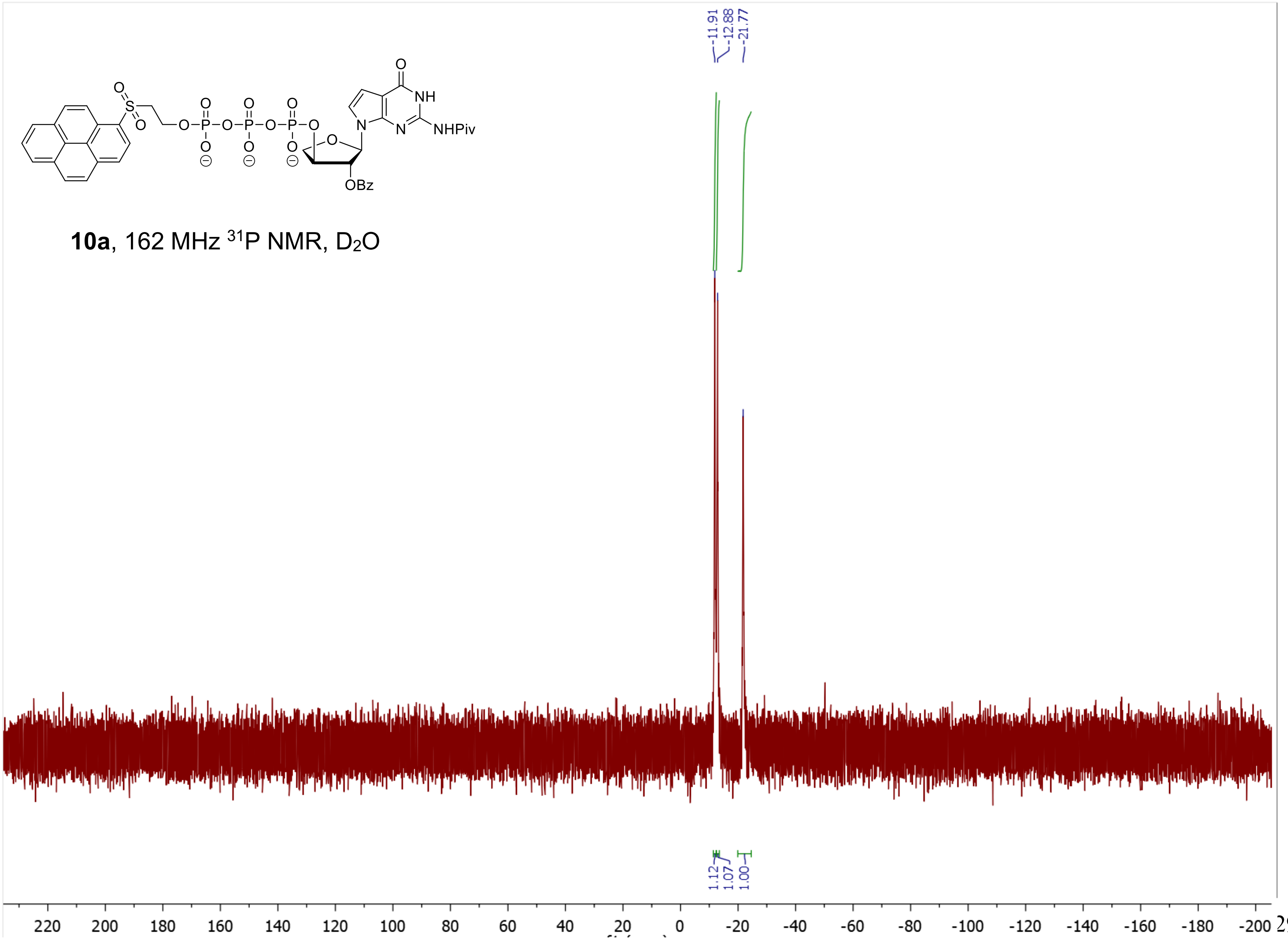

10a, 162 MHz ^{31}P NMR, D_2O

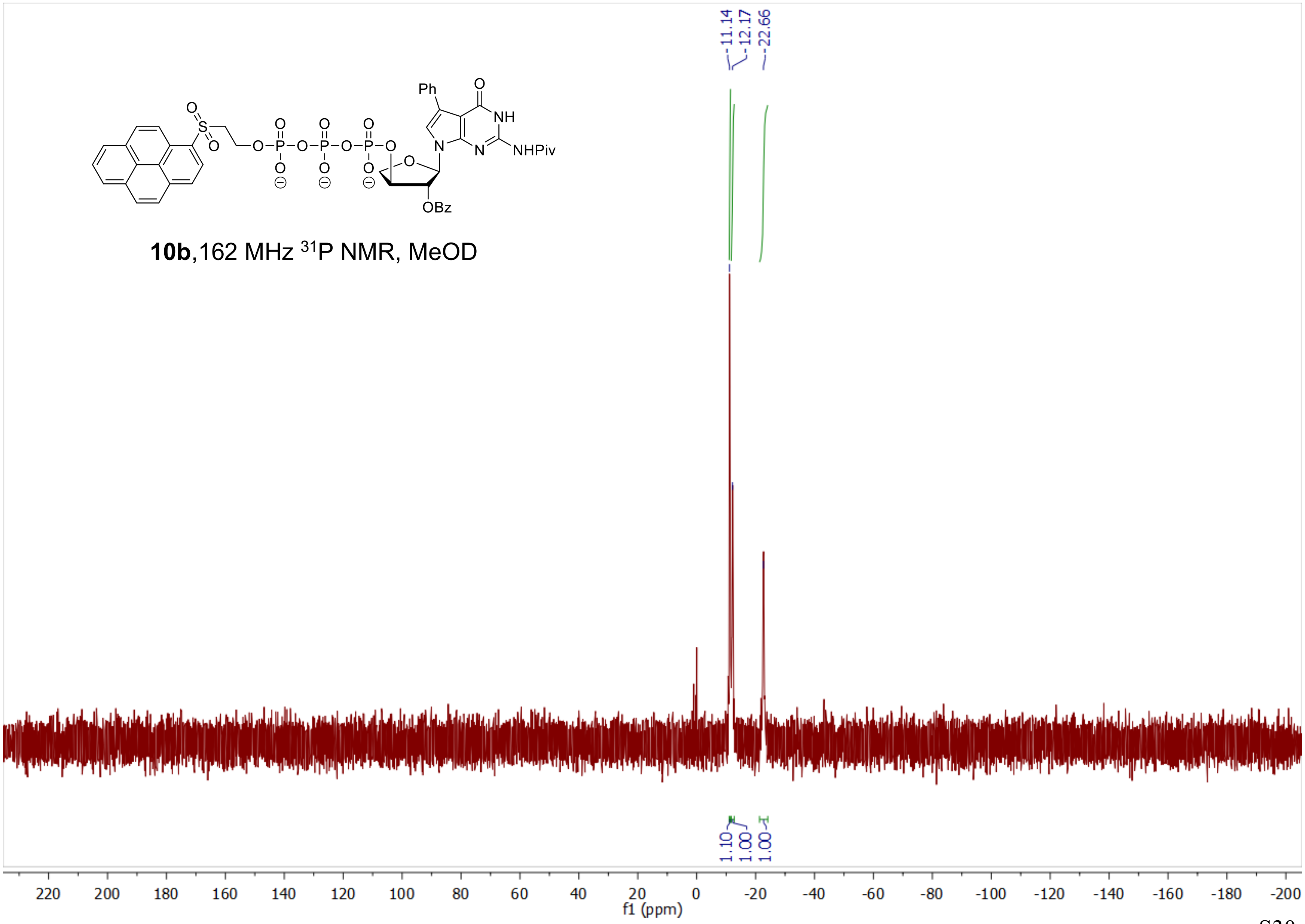

Ph
O
NH
NHPiv
N
N
O
O
S
O
O-P-O-P-O-P-O
OBz
10b,162 MHz ³¹P NMR, MeOD
-11.14
-12.17
-22.66
1.10
1.00
1.00
220 200 180 160 140 120 100 80 60 40 20 0 -20 -40 -60 -80 -100 -120 -140 -160 -180 -200
f1 (ppm)

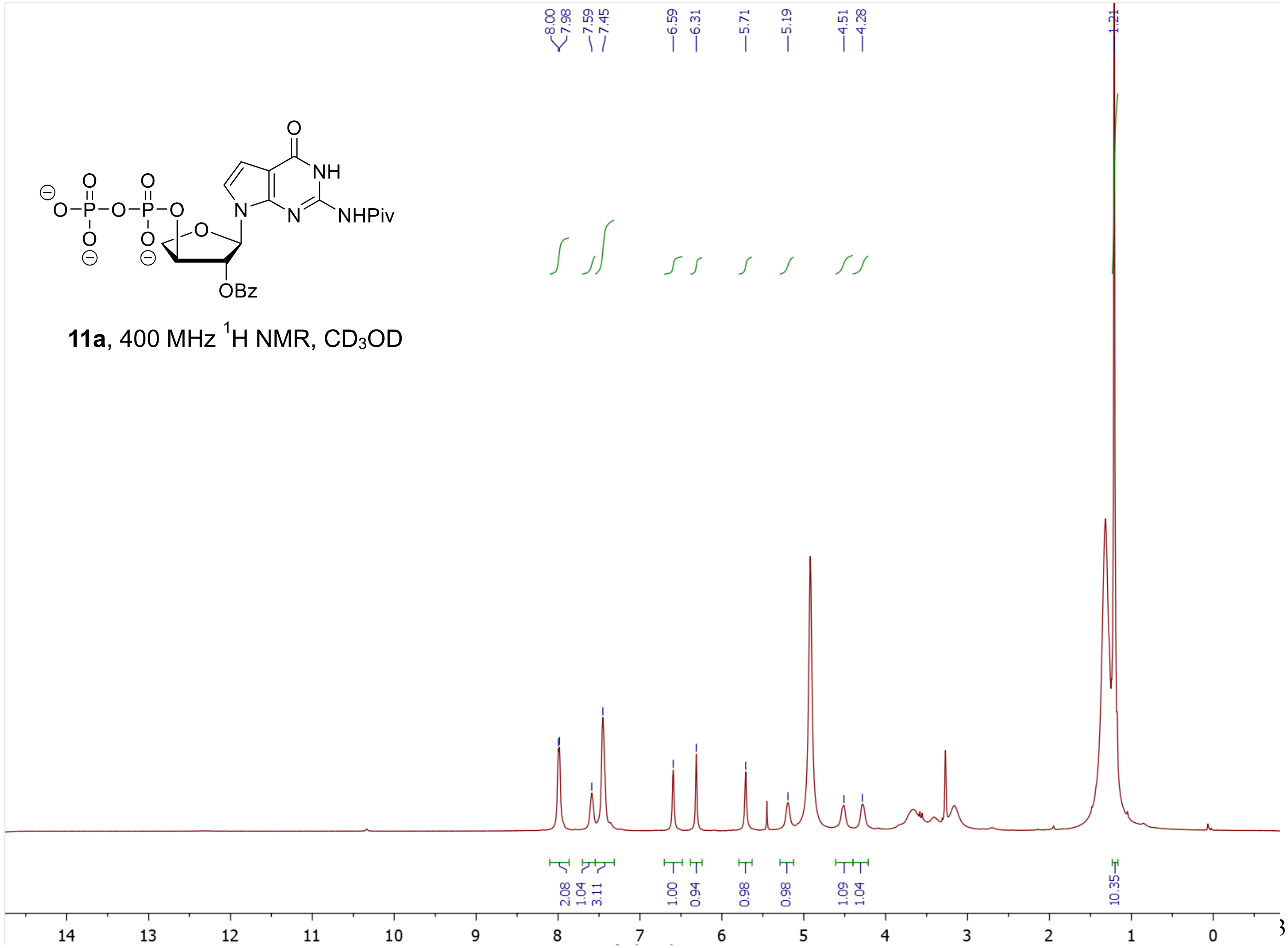

11a, 400 MHz ^{1}H NMR, CD_3OD

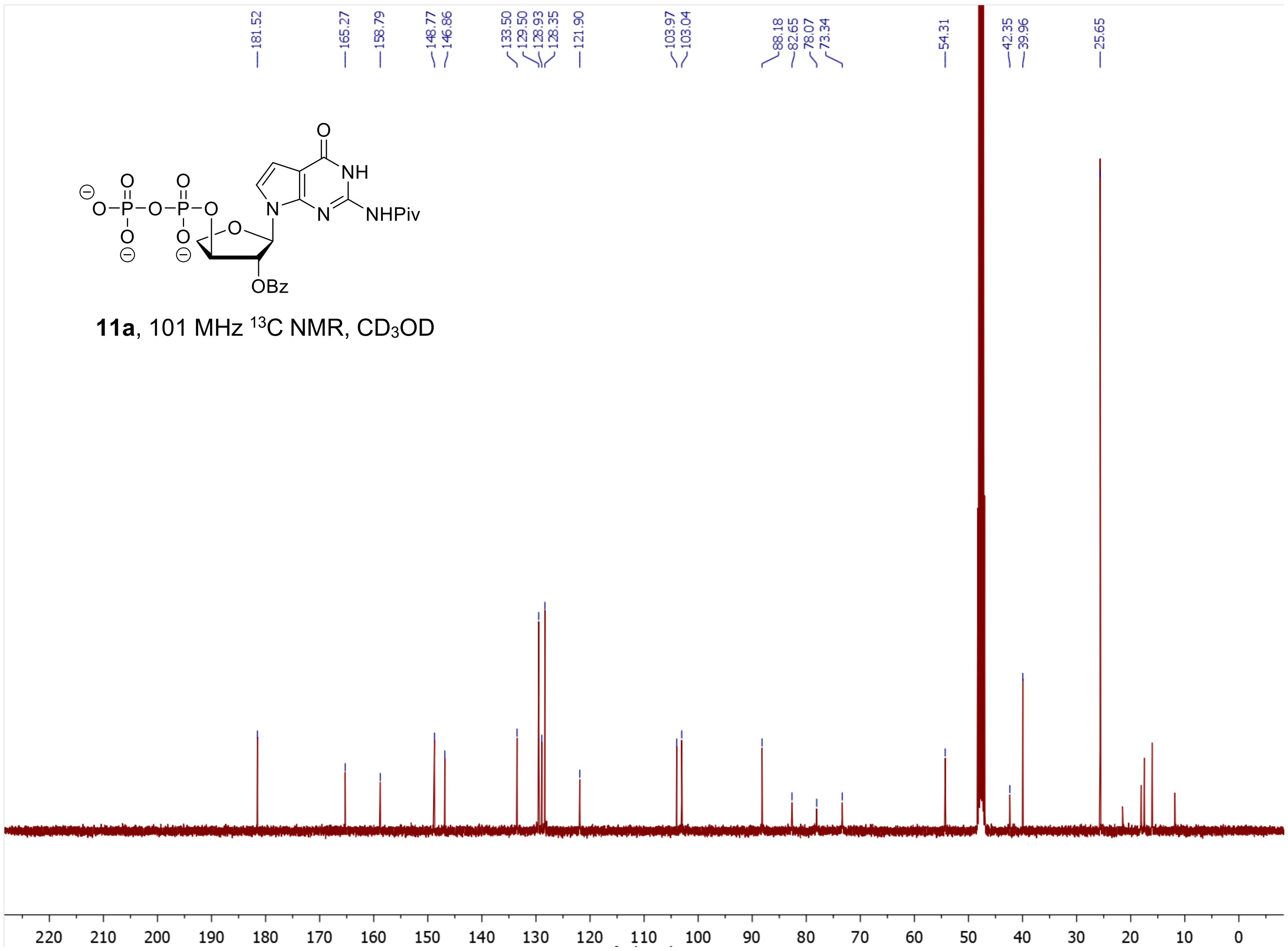

11a, 101 MHz ^{13}C NMR, CD_3OD
O
NH
NHPiv
N
N
O
O
O
P
P
O
O
O
O
O
OBz
181.52
165.27
158.79
148.77
146.86
133.50
129.50
128.93
128.35
121.90
103.97
103.04
88.18
82.65
78.07
73.34
54.31
42.35
39.96
25.65
220
210
200
190
180
170
160
150
140
130
120
110
100
90
80
70
60
50
40
30
20
10
0

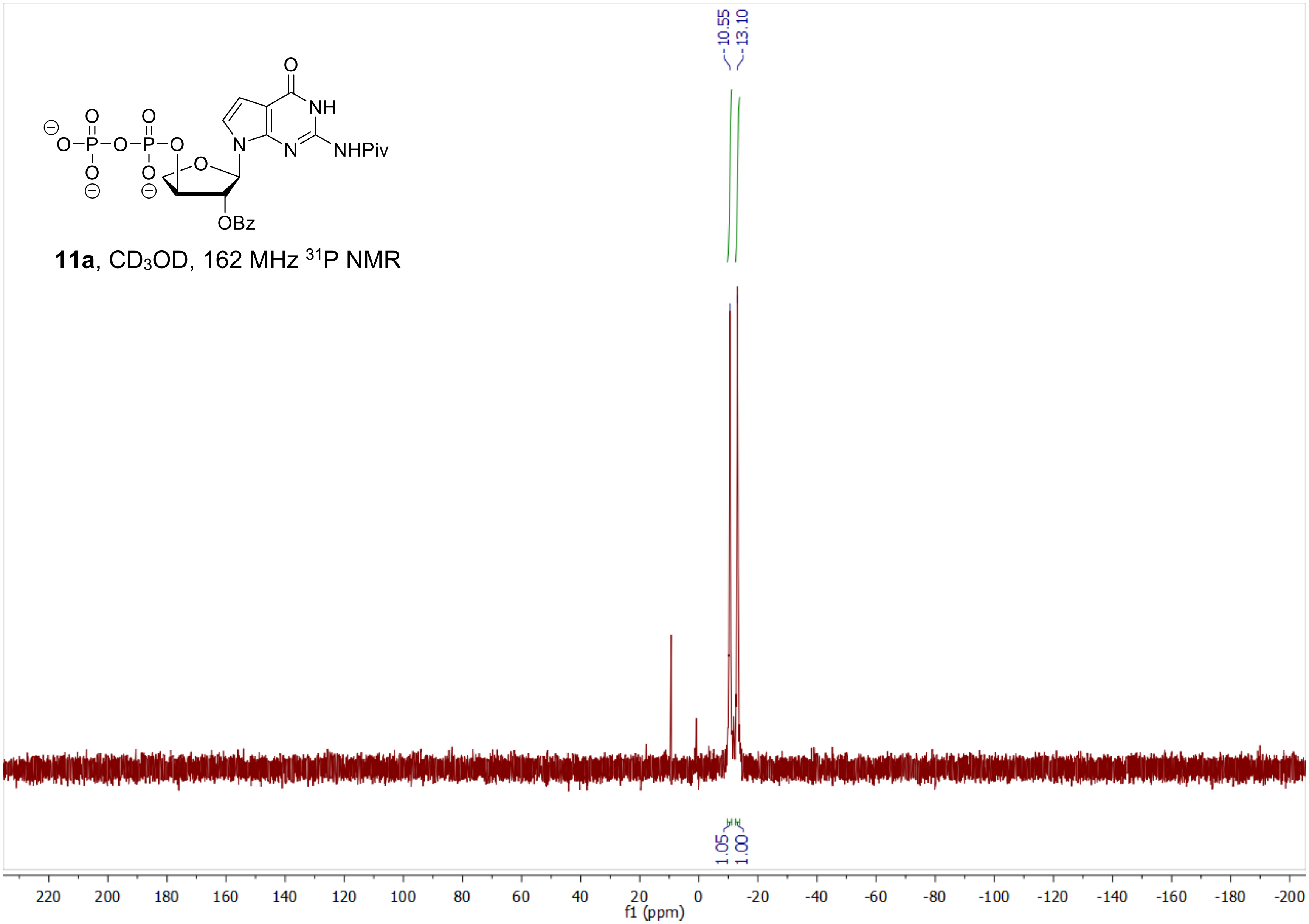

11a, CD_3OD, 162 MHz ^{31}P NMR

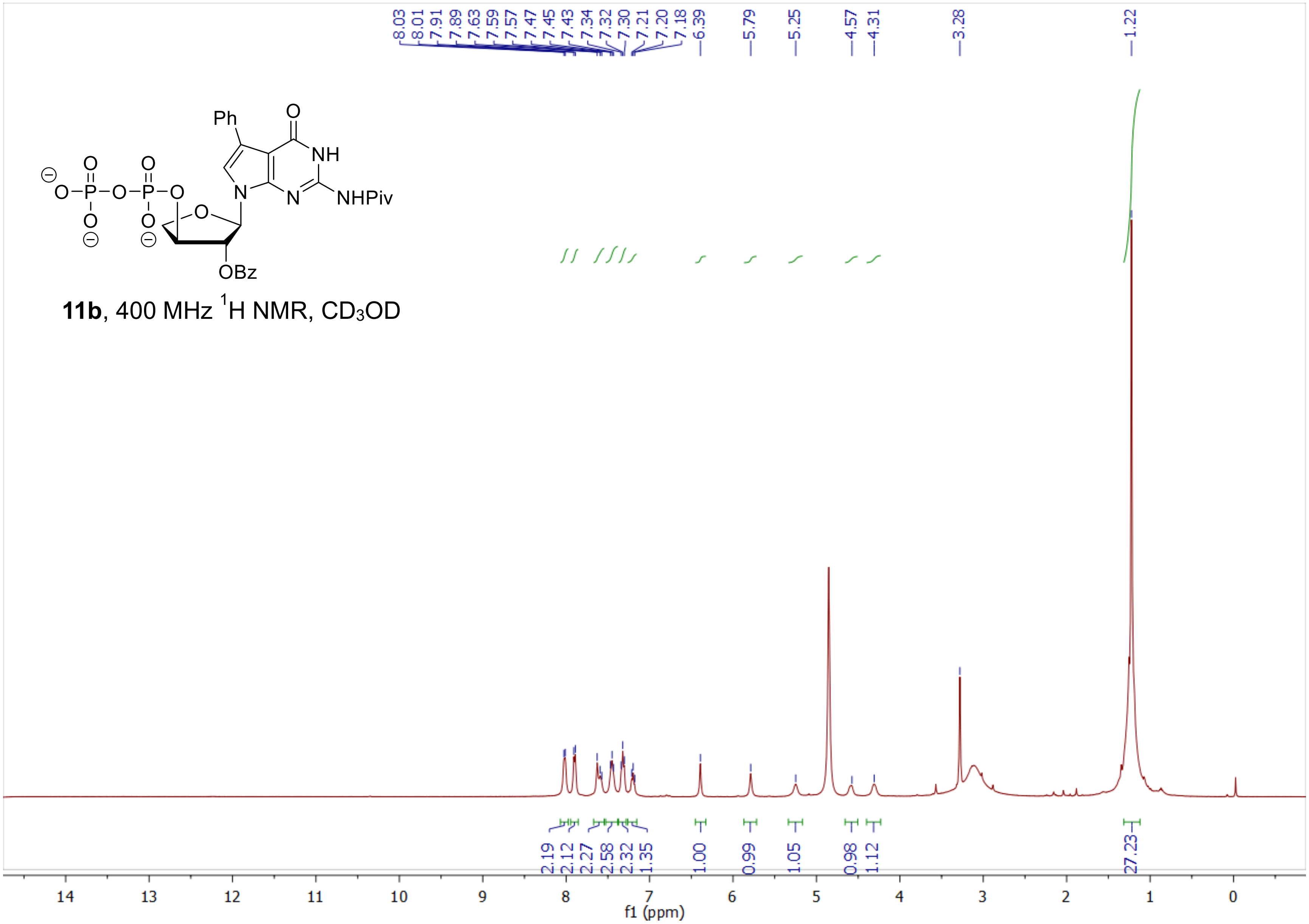
8.03 8.01 7.91 7.89 7.63 7.59 7.57 7.47 7.45 7.43 7.34 7.32 7.30 7.21 7.20 7.18 6.39 5.79 5.25 4.57 4.31 3.28 1.22
Ph
O
NH
NHPiv
N
N
O
O
O
O
P
P
OBz
11b, 400 MHz ^{1}H NMR, CD$_3$OD
2.19 2.12 2.27 2.58 2.32 1.35 1.00 0.99 1.05 0.98 1.12 27.23
14 13 12 11 10 9 8 7 6 5 4 3 2 1 0
f1 (ppm)

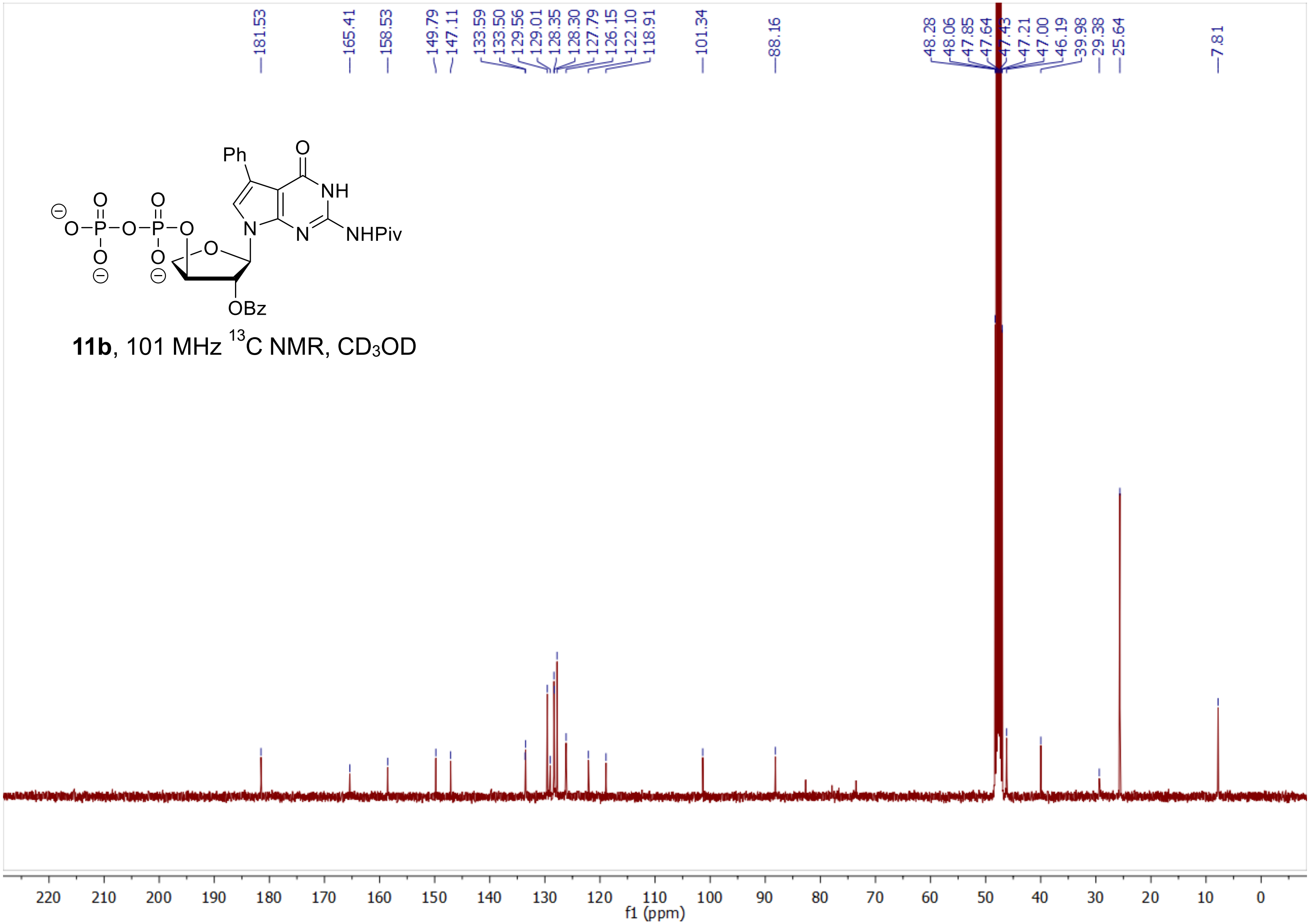

11b, 101 MHz ^{13}C NMR, CD_3OD

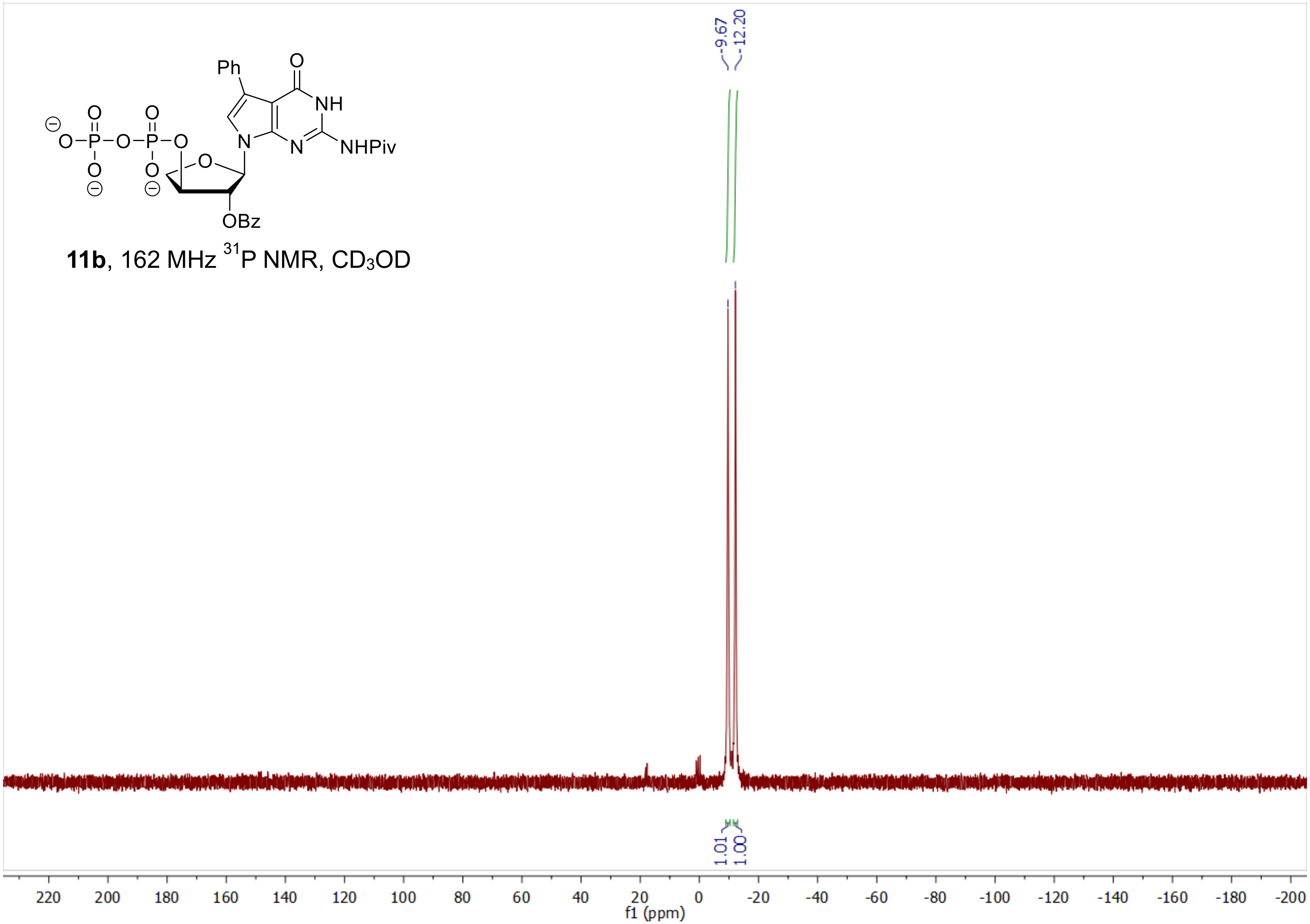
Ph
O
NH
NHPiv
N
N
O
OBz
11b, 162 MHz ^{31}P NMR, CD_3OD
-9.67
-12.20
1.01
1.00
220
200
180
160
140
120
100
80
60
40
20
0
-20
-40
-60
-80
-100
-120
-140
-160
-180
-200
f1 (ppm)

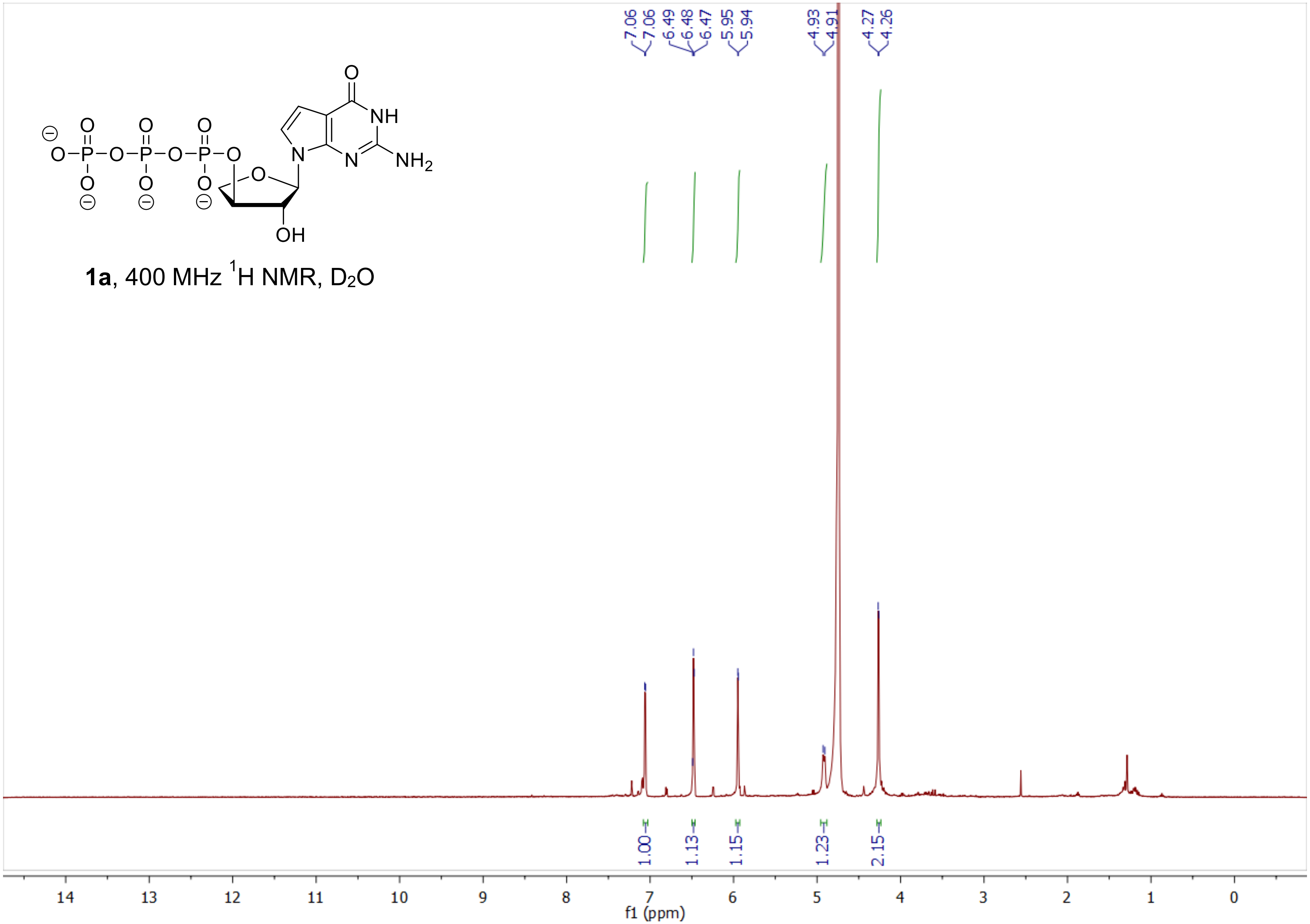

1a, 400 MHz ^{1}H NMR, D$_2$O
7.06
7.06
6.49
6.48
6.47
5.95
5.94
4.93
4.91
4.27
4.26
1.00
1.13
1.15
1.23
2.15
14 13 12 11 10 9 8 7 6 5 4 3 2 1 0
f1 (ppm)

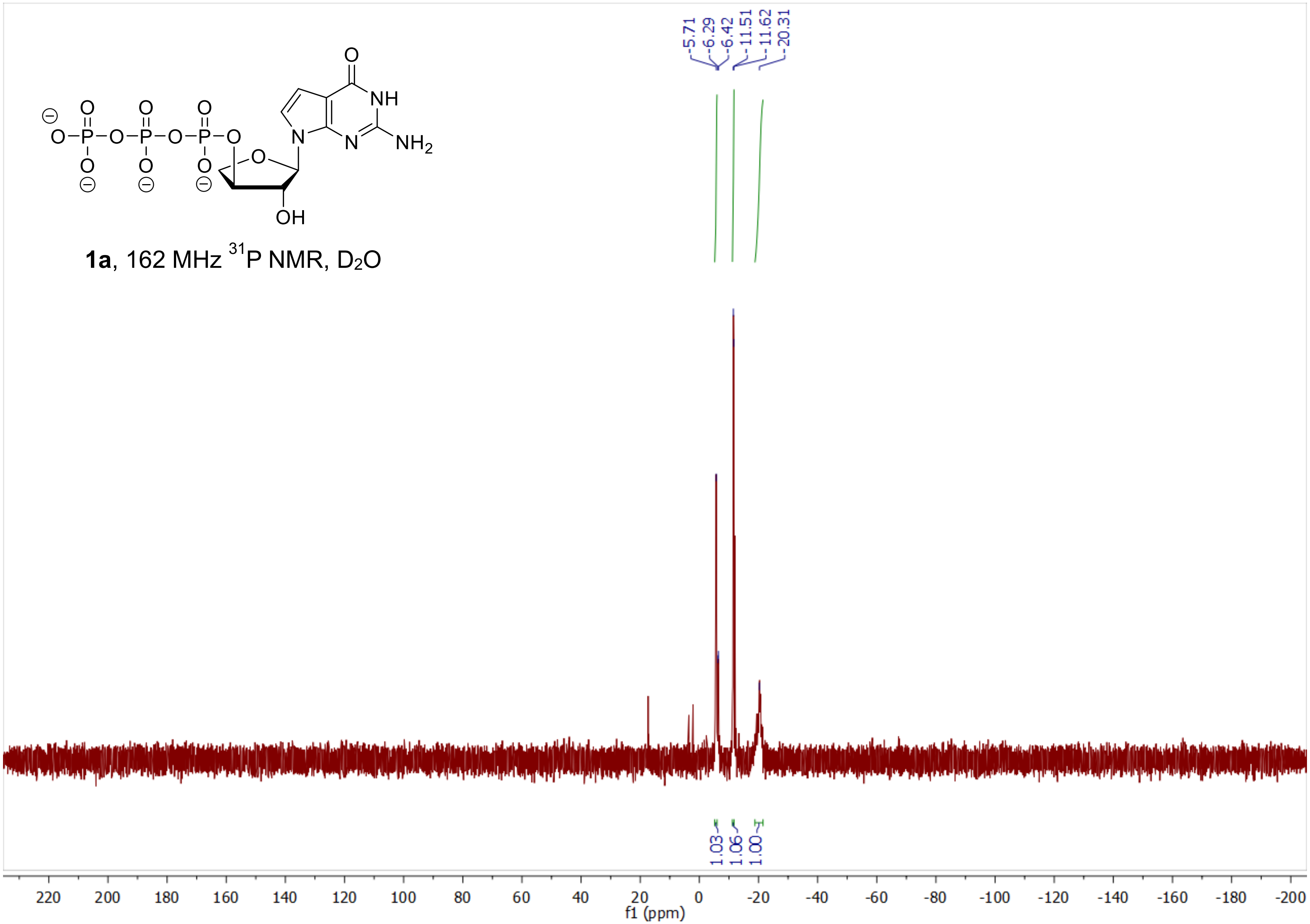

1a, 162 MHz ^{31}P NMR, D_2O

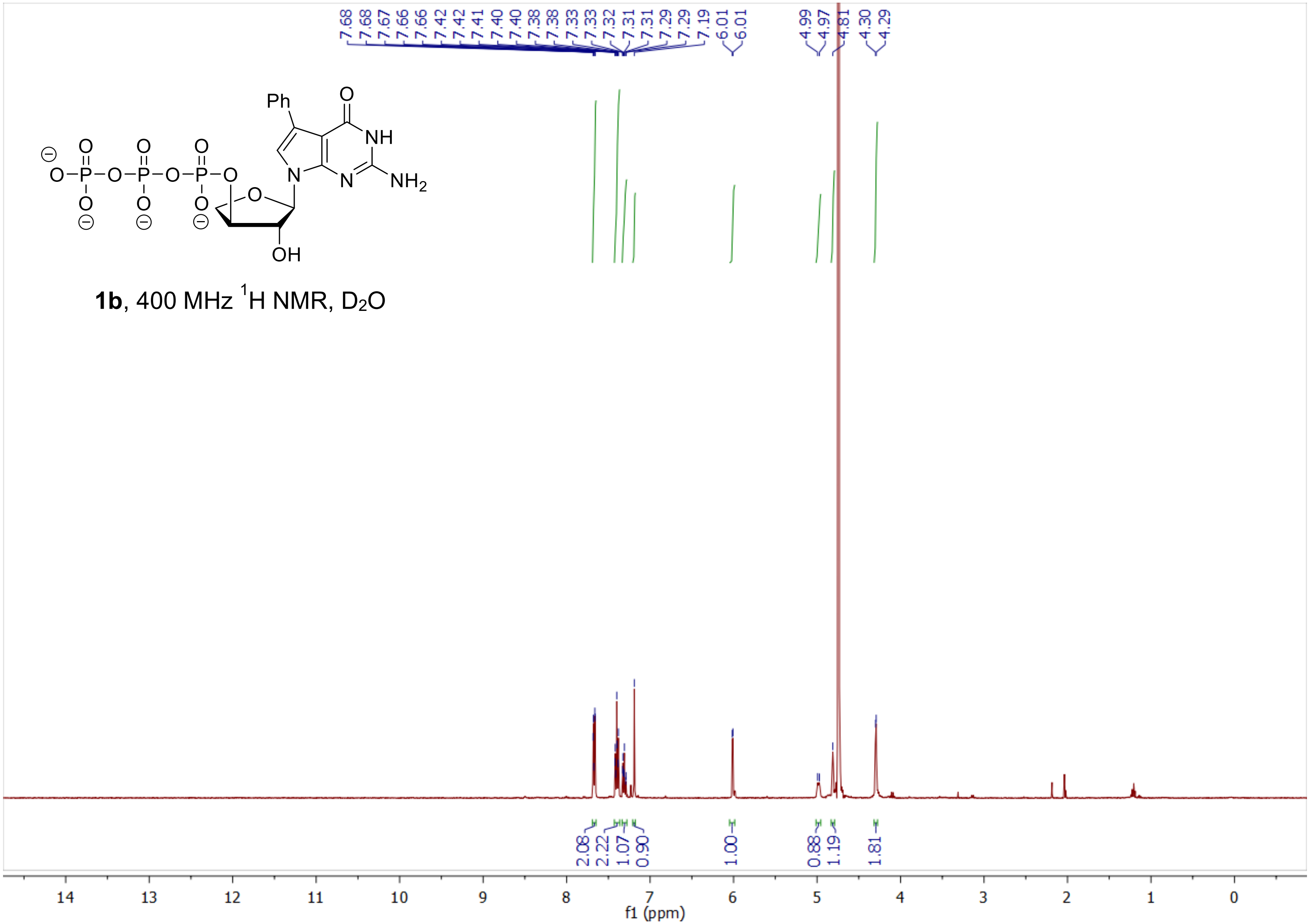

1b, 400 MHz ^{1}H NMR, D_2O

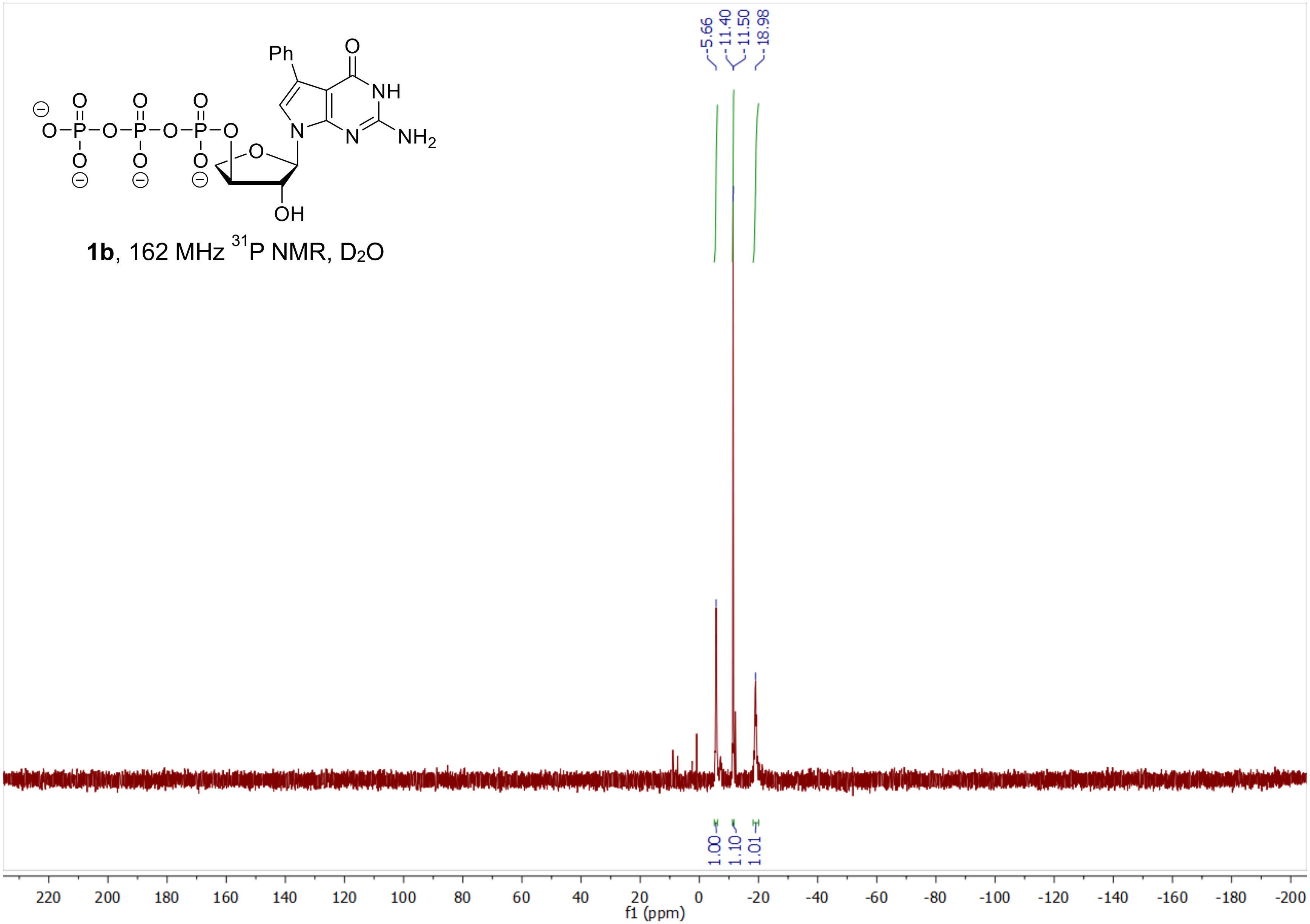

1b, 162 MHz ^{31}P NMR, D_2O

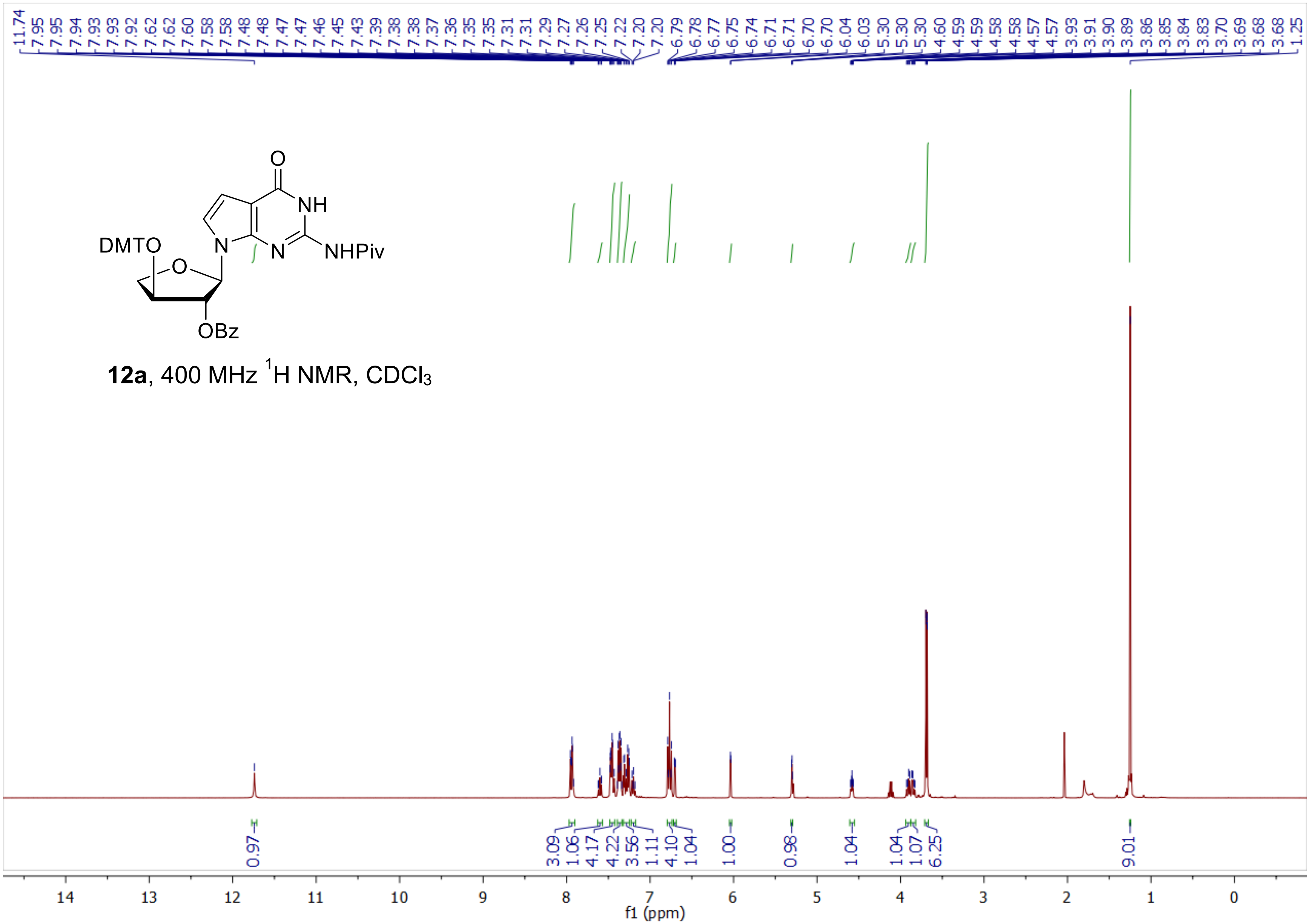
11.74 7.95 7.94 7.93 7.93 7.92 7.62 7.62 7.60 7.58 7.58 7.48 7.48 7.47 7.47 7.46 7.45 7.43 7.39 7.38 7.38 7.37 7.36 7.35 7.35 7.31 7.29 7.27 7.26 7.25 7.22 7.20 7.20 6.79 6.78 6.77 6.75 6.74 6.71 6.71 6.70 6.70 6.04 6.03 5.30 5.30 5.30 4.60 4.59 4.59 4.58 4.58 4.57 4.57 3.93 3.91 3.90 3.89 3.86 3.85 3.84 3.83 3.70 3.69 3.68 3.68 1.25
O
NH
N
N
NHPiv
DMTO
O
OBz
12a, 400 MHz 1H NMR, CDCl3
0.97
3.09
1.06
4.17
4.22
3.56
1.11
4.10
1.04
1.00
0.98
1.04
1.04
1.07
6.25
9.01
14 13 12 11 10 9 8 7 6 5 4 3 2 1 0
f1 (ppm)

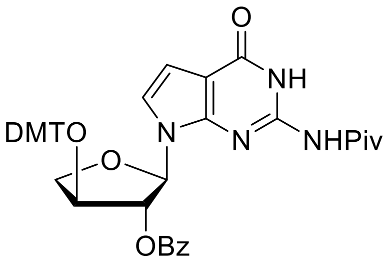

12a, 101 MHz ^{13}C NMR, $CDCl_3$

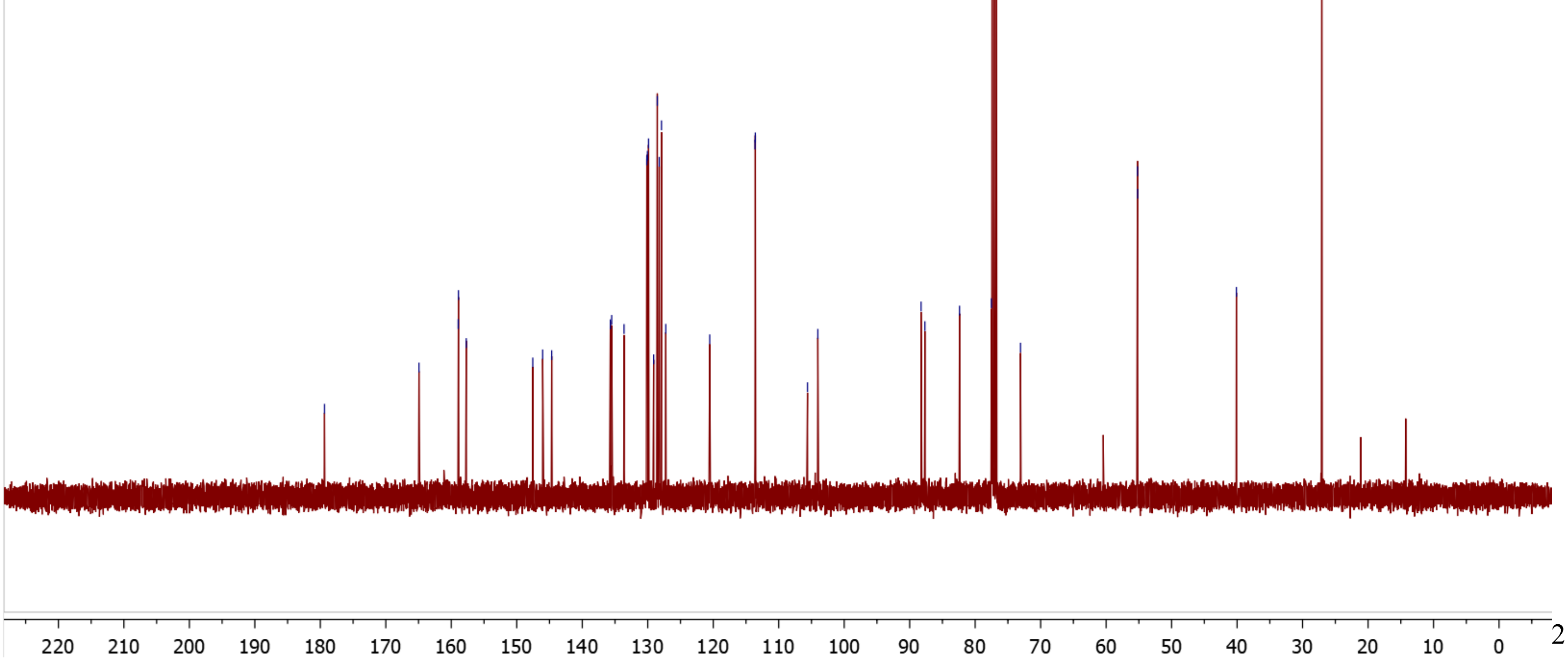

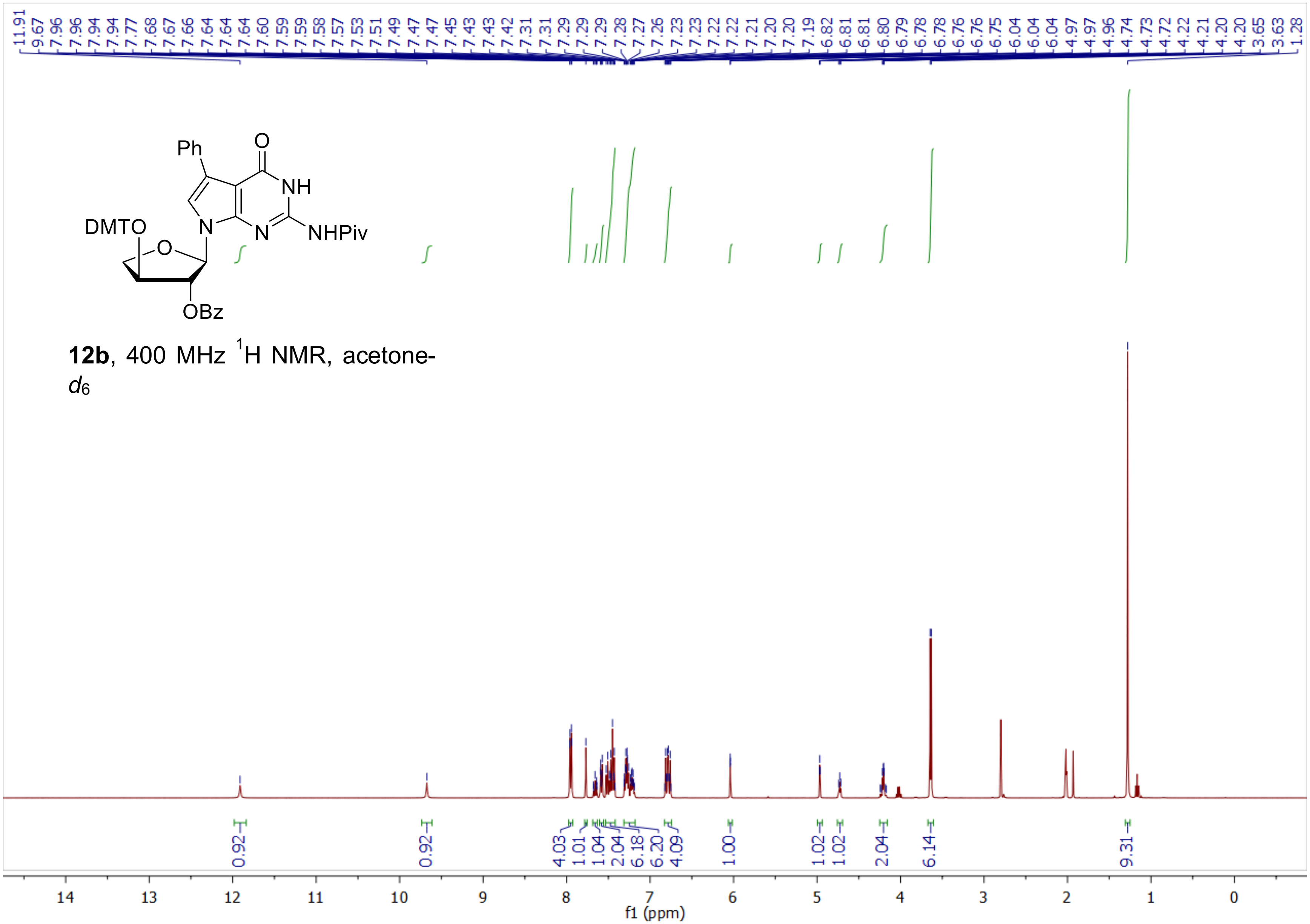

12b, 400 MHz ^{1}H NMR, acetone-d_6

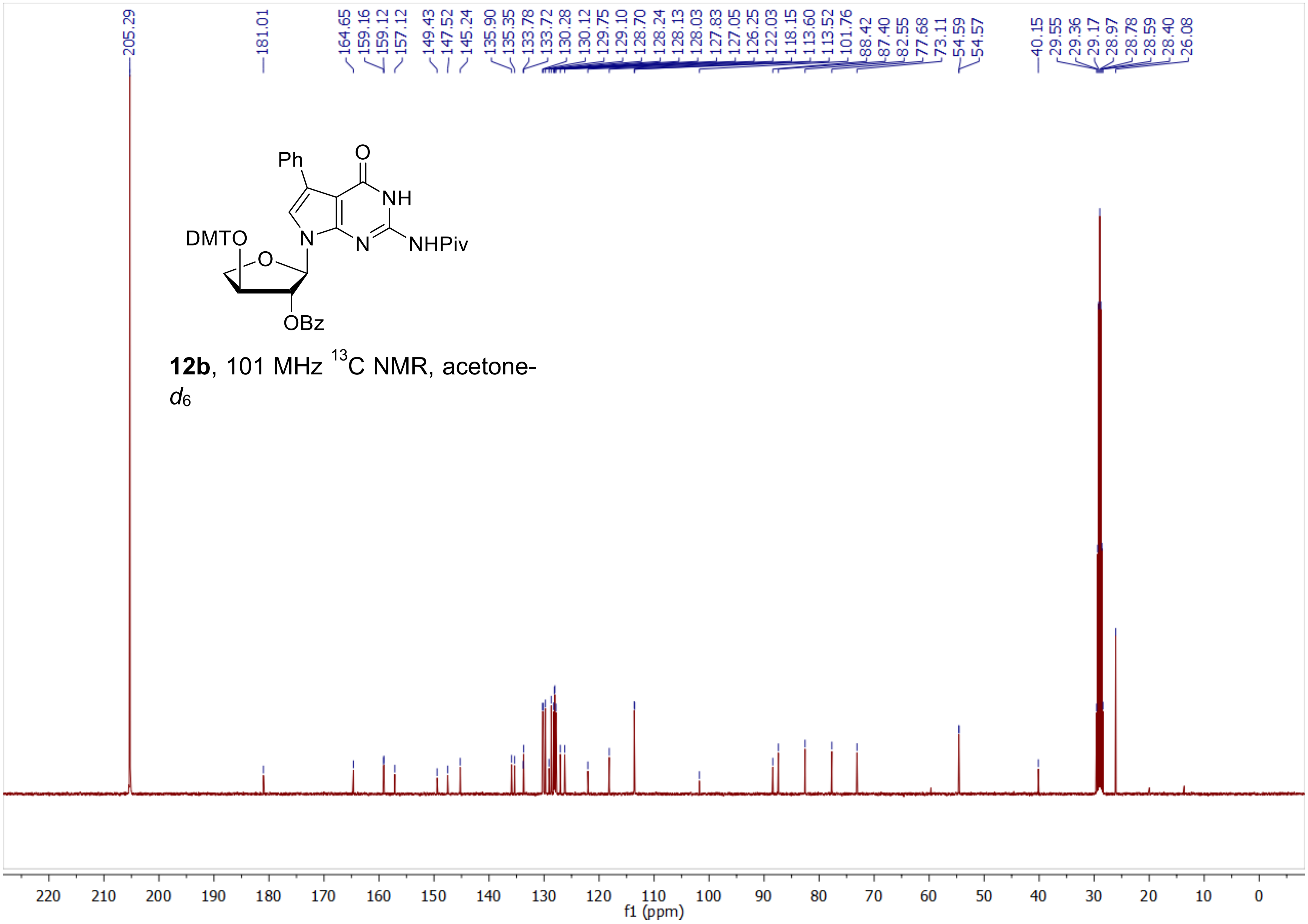

12b, 101 MHz ^{13}C NMR, acetone-*d*$_6$

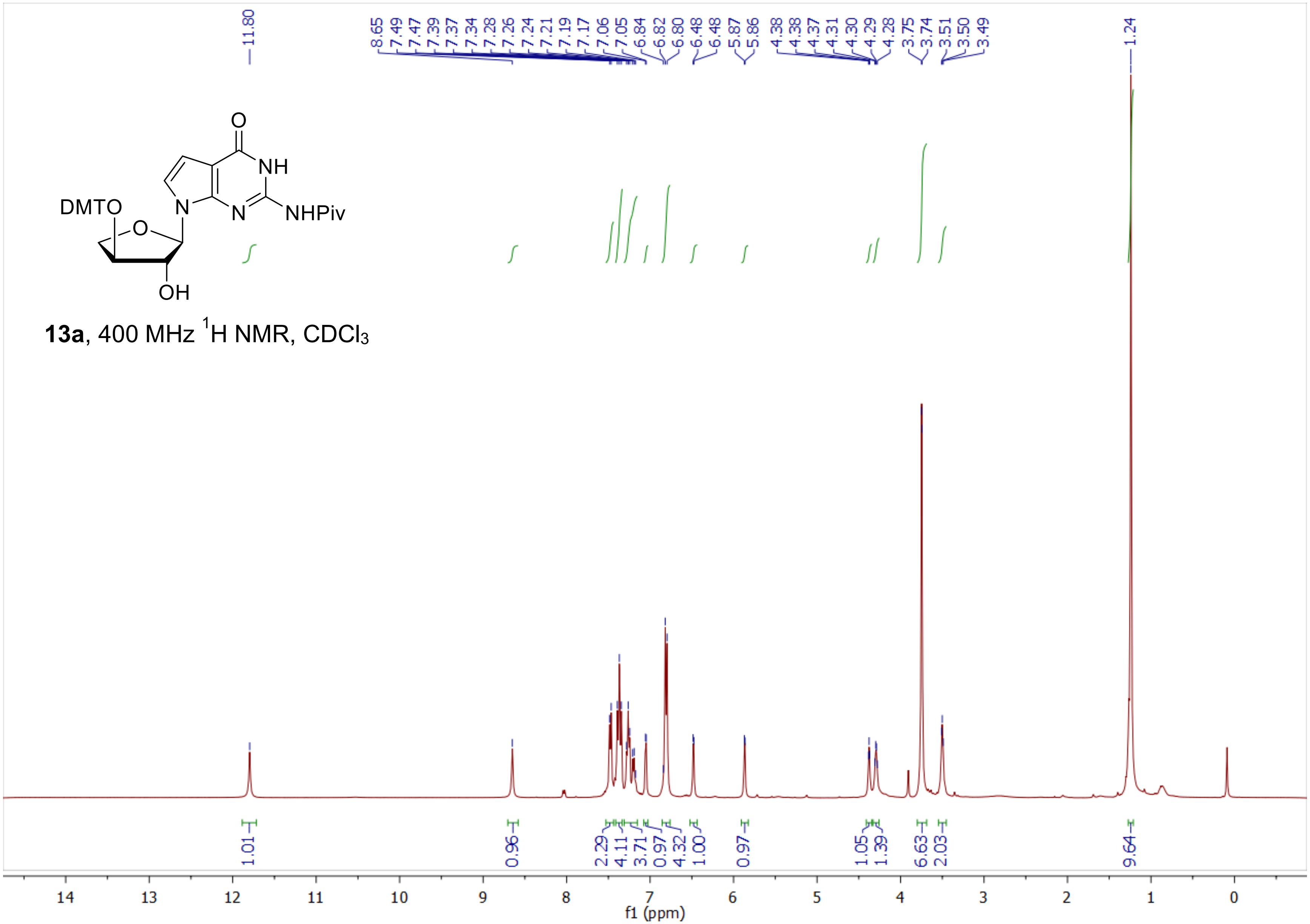
DMTO
O
NH
N
N
NHPiv
OH
13a, 400 MHz 1H NMR, CDCl3
f1 (ppm)

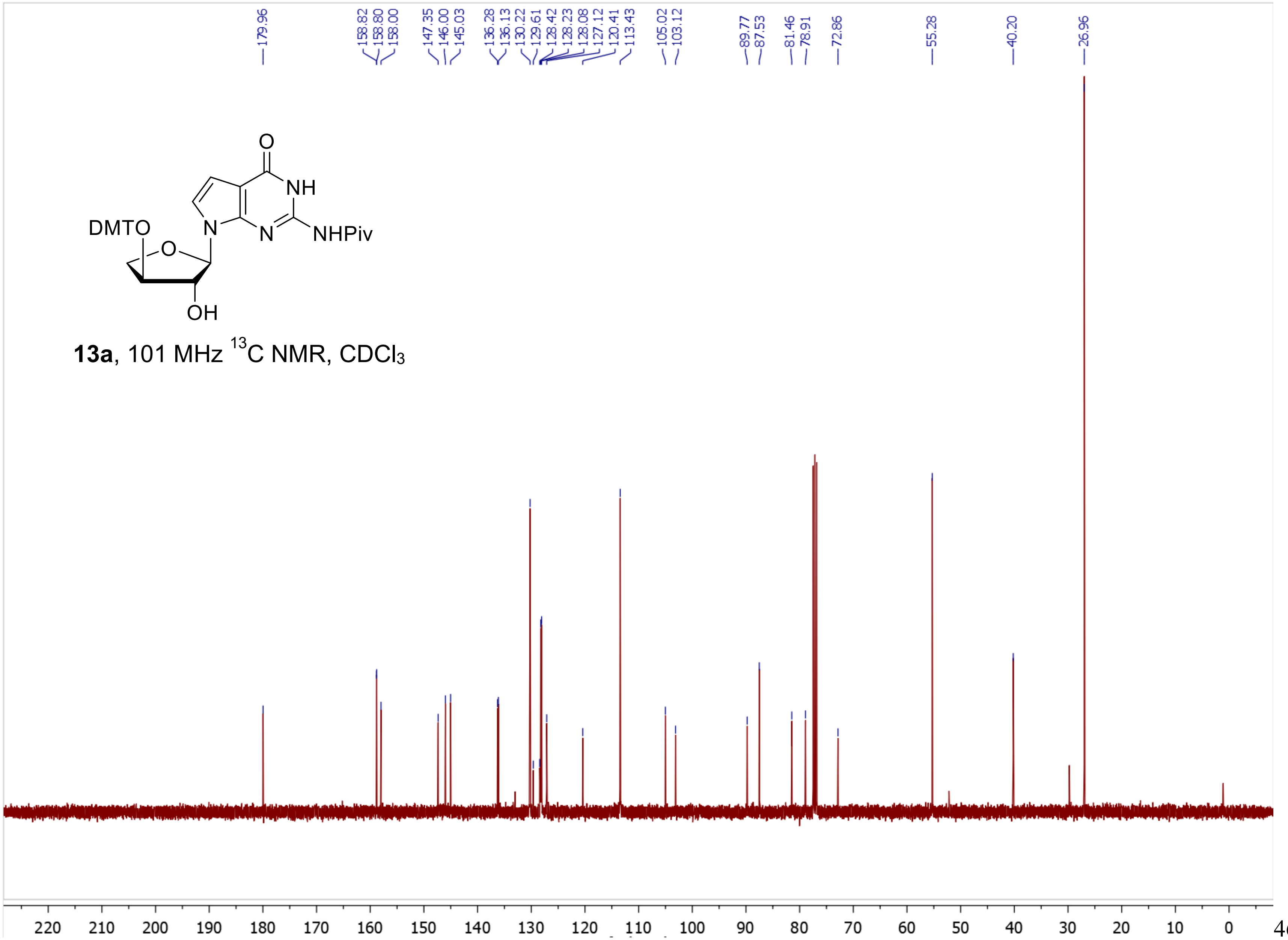
179.96
158.82
158.80
158.00
147.35
146.00
145.03
136.28
136.13
130.22
129.61
128.42
128.23
128.08
127.12
120.41
113.43
105.02
103.12
89.77
87.53
81.46
78.91
72.86
55.28
40.20
26.96
DMTO
O
N
NH
N
NHPiv
OH
13a, 101 MHz 13C NMR, CDCl3
220 210 200 190 180 170 160 150 140 130 120 110 100 90 80 70 60 50 40 30 20 10 0

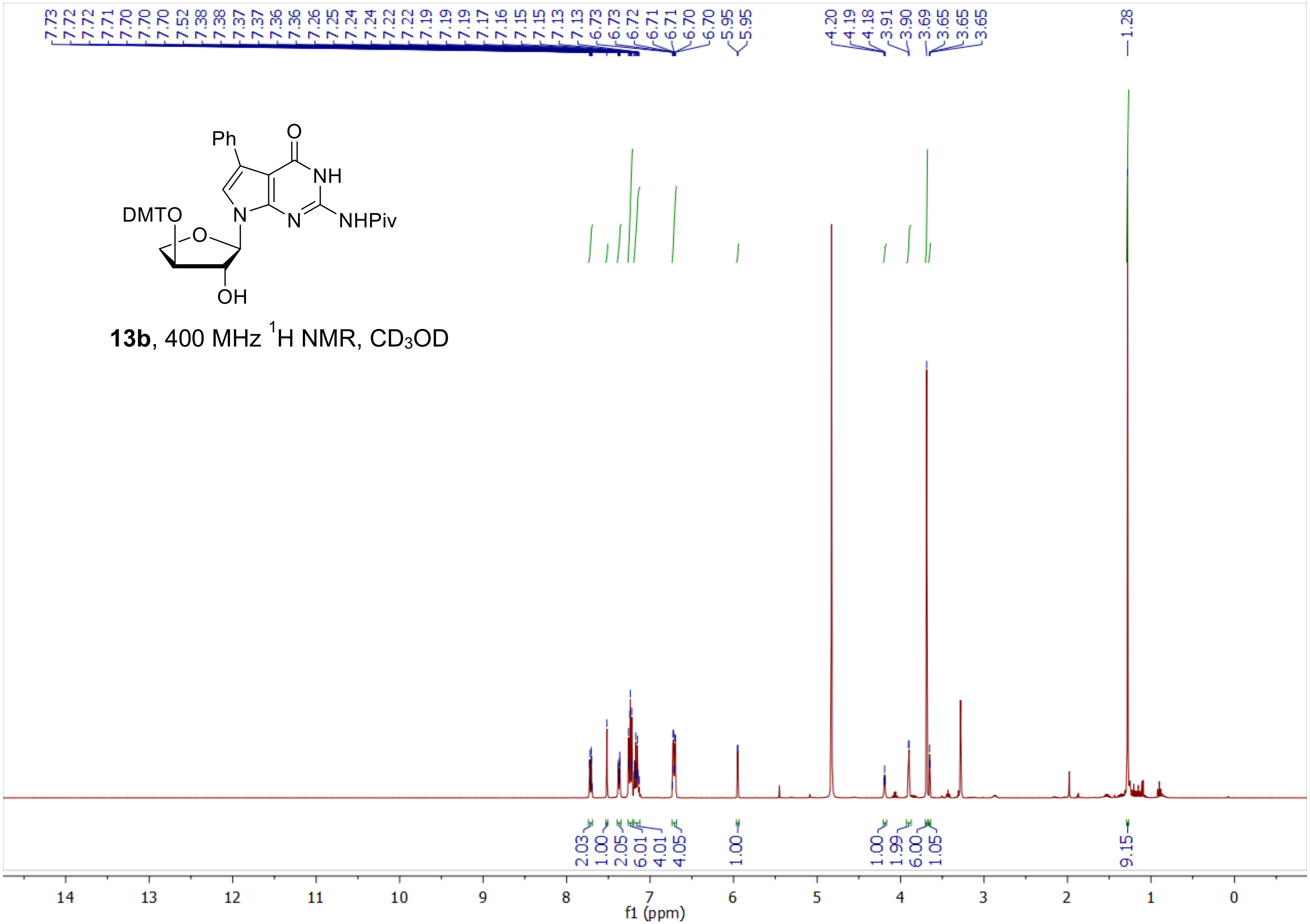

13b, 400 MHz ^{1}H NMR, CD$_3$OD

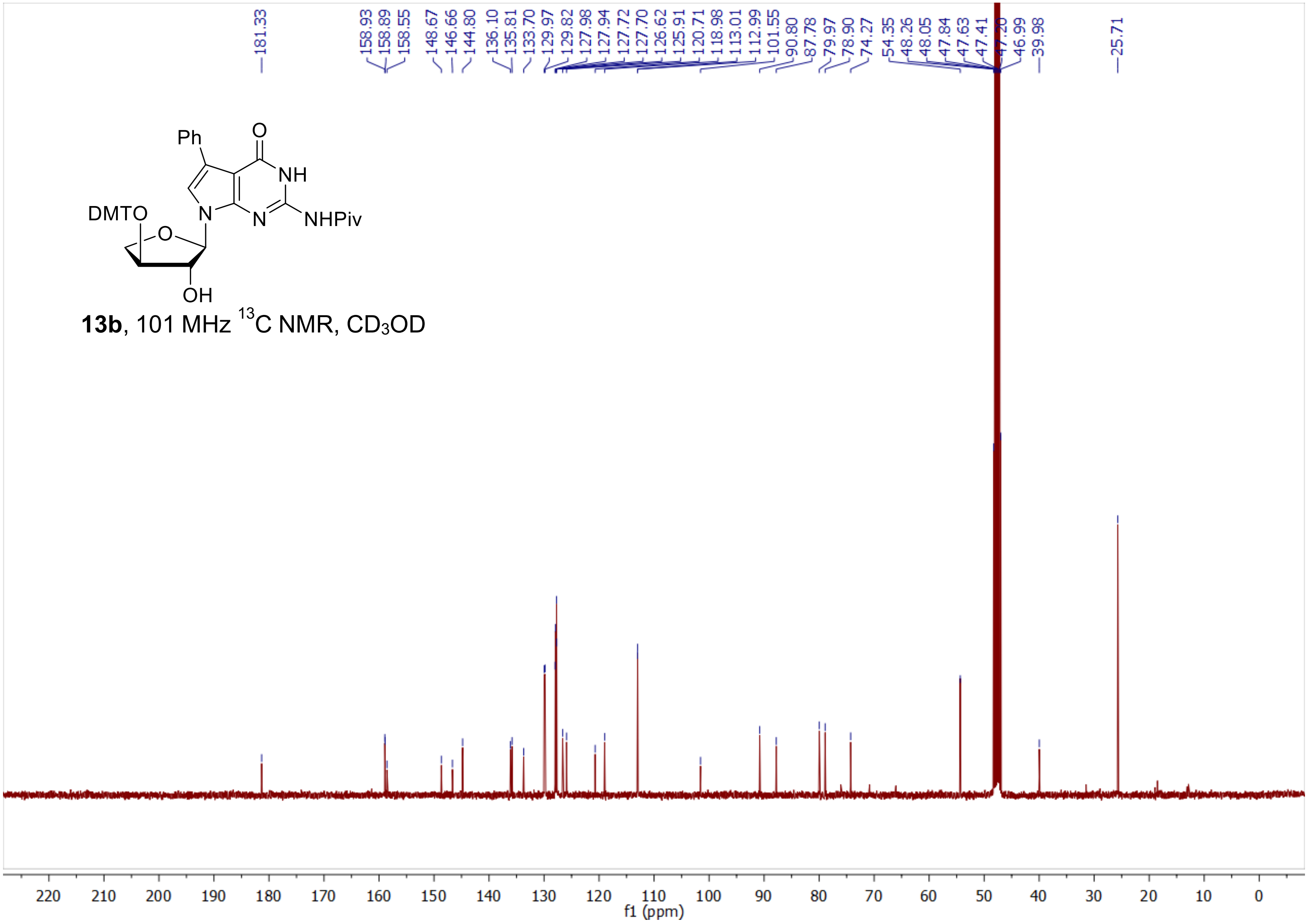

13b, 101 MHz ^{13}C NMR, CD_3OD

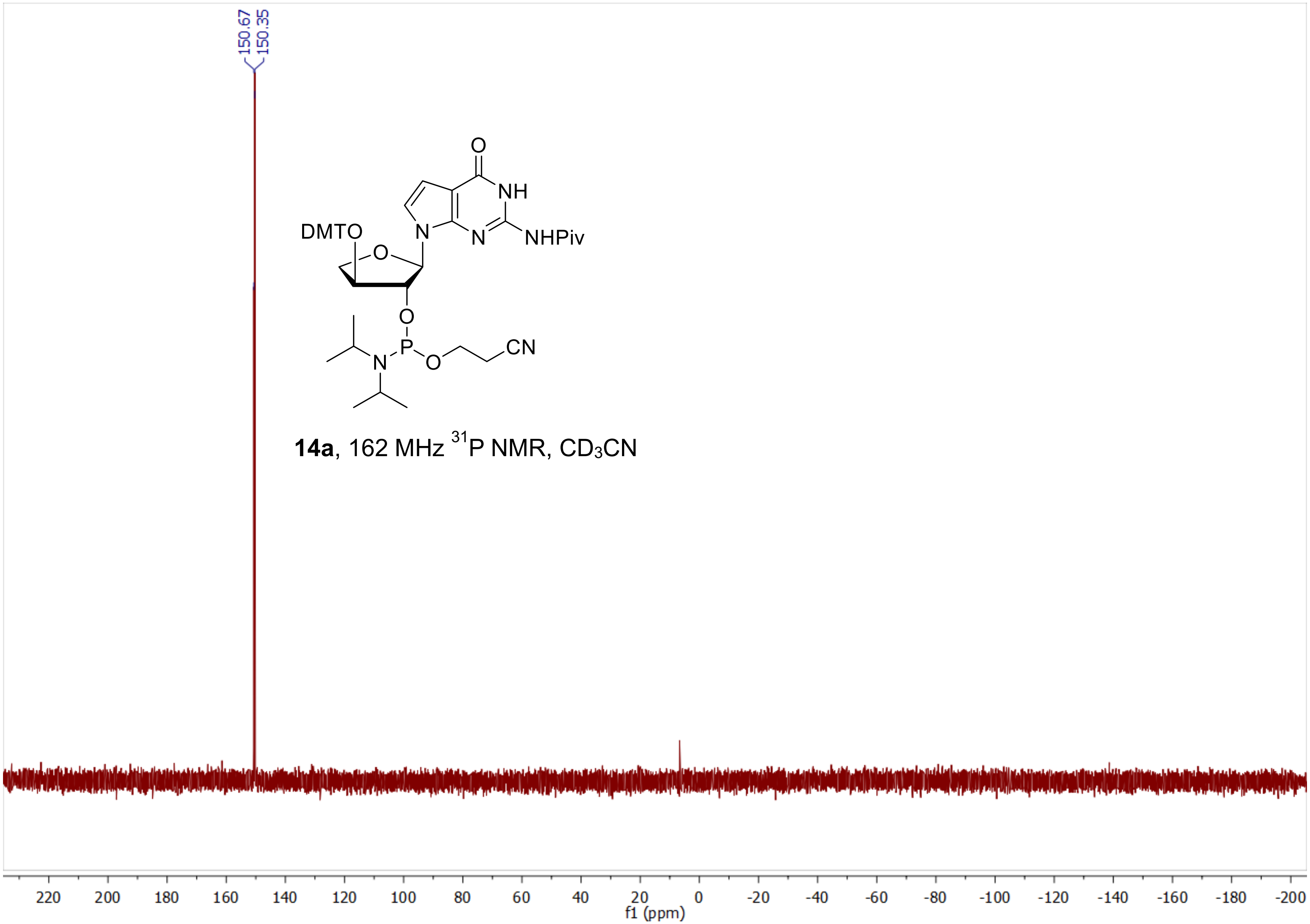

14a, 162 MHz ^{31}P NMR, CD_3CN

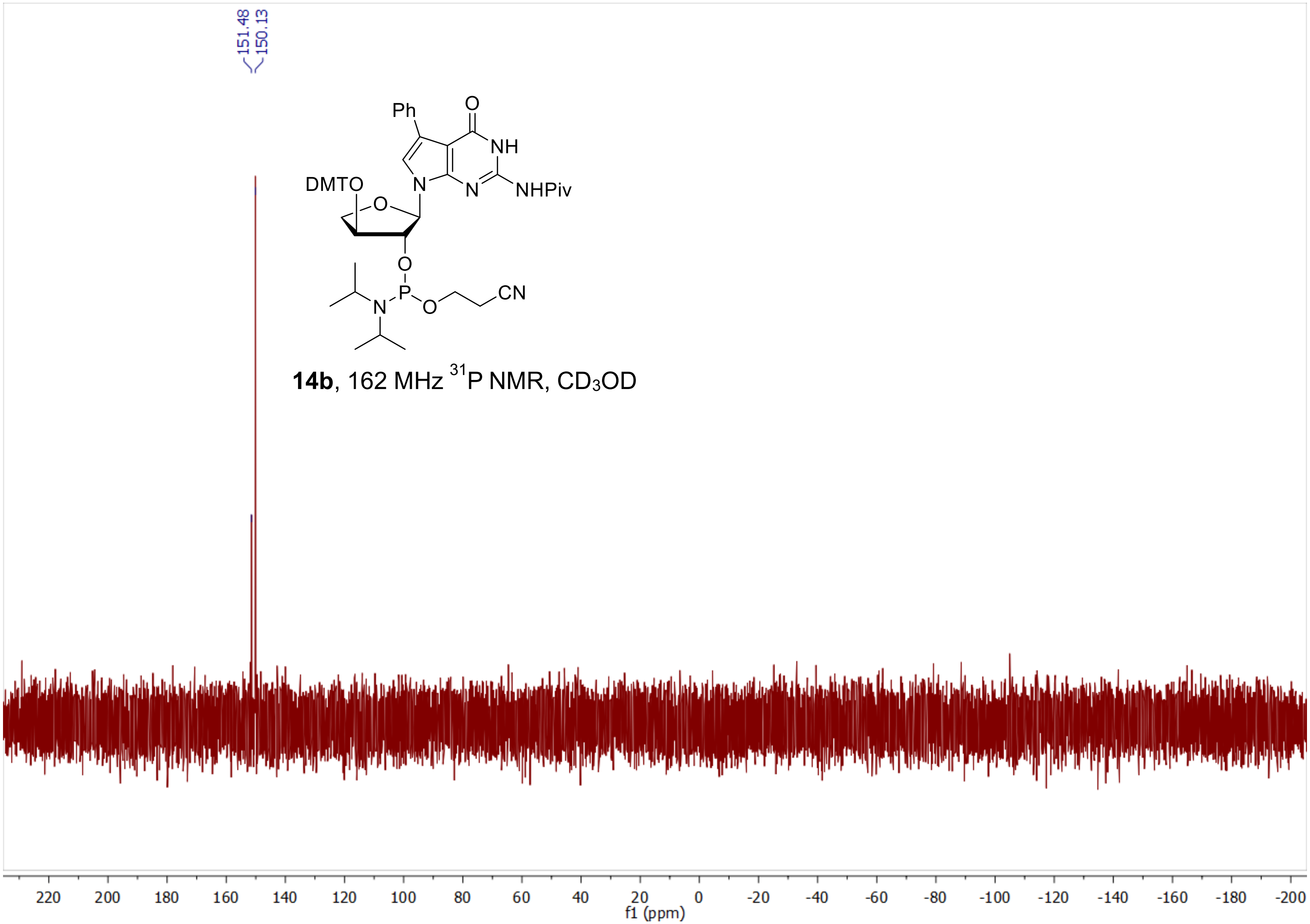
151.48
150.13
Ph
O
NH
DMTO
N
N
NHPiv
O
O
P
CN
N
O
14b, 162 MHz ^{31}P NMR, CD_3OD
220 200 180 160 140 120 100 80 60 40 20 0 -20 -40 -60 -80 -100 -120 -140 -160 -180 -200
f1 (ppm)
